# Exosomal transfer of pro-pyroptotic miR-216a-5p exacerbates anthracycline cardiotoxicity through breast cancer-heart pathological crosstalk

**DOI:** 10.1038/s41392-025-02245-4

**Published:** 2025-05-14

**Authors:** Yan Ma, Yongjun Wang, Renzheng Chen, Yabin Wang, Yan Fang, Cheng Qin, Tianhu Wang, Xiaoying Shen, Tingwen Zhou, Lei Tian, Ting Sun, Li Fan, Xiaoning Wang, Dong Han, Feng Cao

**Affiliations:** 1 Chinese Military Medical School, National Clinical Research Center for Geriatric Diseases, The Second Medical Center, Chinese PLA General Hospital, 100853 Beijing, China; 2 Department of Cardiovascular Surgery, Union Hospital, Tongji Medical College, Huazhong University of Science and Technology, Wuhan, Hubei 430022 China; 3 School of Life Sciences, Fudan University, Shanghai, China. Room 1910, West Guanghua Tower, 220 Handan Road, Shanghai, 200433 China

**Keywords:** Cardiology, Cardiovascular diseases

## Abstract

Doxorubicin (DOX) is the most effective chemotherapeutic for breast cancer, but it is usually associated with severe cardiotoxicity. Further investigation to alleviate its side effects is essential. The present study investigated the mechanism of the cross-organ communication between tumors and the heart and potential intervention targets. Morphological bubble-like protrusions were observed in both adult murine ventricular cardiomyocytes (AMVCs) and human induced pluripotent stem cell-derived cardiomyocytes (hiPSC-CMs) cocultured with breast cancer cells (BCCs), along with elevated expression of pyroptosis-related proteins. Exosomes (EXOs) from DOX-treated BCCs aggravated DOX-induced cardiotoxicity (DOXIC) in an orthotopic mouse model of breast cancer. Blocking miRNAs by knocking down Rab27a or inhibiting the release of EXOs in cancer tissue by Dicer enzyme knockout attenuated this additional injury effect. Exosomal miRNA sequencing revealed that miR-216a-5p is especially upregulated in EXOs from DOX-induced BCCs. Mechanistically, miR-216a-5p was upregulated by enhanced transcription mediated by DOX-induced AMP-dependent transcription factor 3 (ATF3) and packaged into EXOs by splicing factor 3b subunit 4 (SF3B4) in BCCs. Itchy E3 ubiquitin-protein ligase (ITCH) was identified as a novel downstream target mRNA of miR-216a-5p. ITCH negatively mediated thioredoxin-interacting protein (TXNIP) ubiquitination to activate the NOD-, LRR- and pyrin domain-containing protein 3 (NLRP3) inflammasome pathway, ultimately leading to cardiomyocyte pyroptosis. Our findings revealed novel cross-organ pathogenic communication between breast cancer and the heart through the exosomal miR-216a-5p-mediated ITCH/TXNIP/NLRP3 pathway, which drives cardiomyocyte pyroptosis. These findings suggest that targeting myocardial miR-216a-5p or blocking harmful EXOs from breast cancer is a potential therapeutic strategy for alleviating DOXIC.

## Introduction

Cardiovascular disease (CVD) and breast cancer represent the two most significant threats to women’s health worldwide.^1,2^ The remarkable advances in breast cancer detection and treatment modalities have resulted in a growing population of breast cancer survivors who now face an increased risk of long-term cardiovascular complications stemming from cancer chemotherapy.^3^ This emerging clinical challenge has raised substantial concerns that these therapy-induced cardiovascular complications may lead to premature morbidity and mortality among cancer survivors.^4,5^ Chemotherapeutic agents can induce various forms of cardiotoxicity, both acute and delayed, manifesting as heart failure, hypertension, arrhythmias, pericarditis, and other cardiac conditions.^6^ Among these, doxorubicin-induced cardiotoxicity (DOXIC) stands as the most representative and clinically significant. Doxorubicin (DOX), a widely used anthracycline antibiotic employed as an antineoplastic agent in clinical settings, has demonstrated remarkable efficacy against various malignancies including breast cancer, leukemia, and lymphoma. However, its therapeutic value is substantially limited by its potential to cause life-threatening cardiotoxicity.^7^ Clinical evidence from three Phase III studies demonstrates that patients receiving DOX treatment experienced a 26% higher incidence of congestive heart failure compared to those in the placebo group.^8^ The clinical manifestations of DOXIC include deteriorated cardiac function, dilated cardiomyopathy, and in severe cases, congestive heart failure.^9^ Therefore, elucidating the underlying mechanisms of DOXIC is crucial for developing more effective therapeutic strategies aimed at minimizing cardiotoxicity and reducing chemotherapy-related complications.

A growing body of evidence suggests that organ-to-organ communication may establish critical links between tumors and the heart in the pathological processes underlying onco-cardiac comorbidities.^1,7,10,11^ This bidirectional relationship has been demonstrated in several experimental models. For instance, in mice with orthotopic breast cancer concurrent with myocardial infarction (MI), the cardiac injury promotes immune cell reprogramming and significantly increases mammary cancer growth and metastatic potential.^10^ Conversely, in a mouse model of spontaneous breast cancer, the expression of SERCA2a is notably reduced in the left ventricle, resulting in defective calcium handling in cardiomyocytes and consequent left ventricular dysfunction.^11^ Further emphasizing this bidirectional relationship, our previous research revealed that the tumor-suppressive RNA circITCH functions by sponging miR-330-5p, thereby protecting the heart from DOX-induced damage through increased expression of several cardioprotective factors, including SIRT6, survivin, and SERCA2a.^7^ While DOX has been shown to directly damage cardiomyocytes through multiple mechanisms, including mitochondrial dysregulation, lipid peroxidation, DNA damage, and calcium overload,^12^ the possibility that pathological communication between the heart and tumor contributes to DOXIC progression remains inadequately explored and warrants comprehensive investigation. Understanding these complex interactions between cancer and the cardiovascular system is critical for developing integrated therapeutic approaches that can simultaneously address both malignancy and cardioprotection, particularly in the context of anthracycline-based treatment regimens.

Exosomes (EXOs) represent membrane-structured extracellular vesicles secreted by eukaryotic cells. These nanovesicles, typically ranging from 30 to 150 nm in diameter, are increasingly recognized as crucial mediators of cell-to-cell and interorgan communication through their capacity to transfer bioactive molecules, including proteins, various RNA species, and metabolites.^13,14^ Among the diverse molecular cargo transmitted *via* EXOs, microRNAs (miRNAs) have emerged as the most extensively studied molecular class, partly due to their ubiquitous regulatory roles in gene expression across tissues and disease states.^15^ EXOs can be modulated by donor cells and exchange or transmit information to recipient cells in healthy tissue, yet under pathological conditions, exosomes can influence disease progression.^16^ Tumor cells frequently release EXOs into both the tumor microenvironment and patient body fluids, facilitating communication between cancer cells and other cellular populations.^17–19^ Recent investigations have demonstrated that breast cancer-derived EXOs can actively promote cancer progression or induce the production of proinflammatory cytokines by macrophages, highlighting their role in shaping the immune response within the tumor microenvironment.^20,21^ The pathological communication between tumors and the heart may similarly be facilitated by exosomal transfer. Indeed, recent studies have indicated that EXOs secreted by the myocardium following myocardial infarction can stimulate tumor growth in distant sites.^22^ However, it remains unknown whether and through what mechanisms breast cancer-derived EXOs participate in DOXIC development and progression, particularly in the context of anthracycline-based chemotherapy. The identification of specific exosomal cargoes that mediate this tumor-heart communication could potentially reveal novel therapeutic targets for mitigating cardiotoxicity in cancer patients undergoing chemotherapy.

In this study, we investigated EXO-mediated pathological crosstalk between breast cancer and cardiac tissue in doxorubicin-induced cardiotoxicity (DOXIC) through both in vitro molecular mechanistic investigations and in vivo proof-of-concept animal models. Our findings demonstrated that EXO-transferred miR-216a-5p functions as a pyroptosis sensitizer in cardiomyocytes via the ITCH/TXNIP/NLRP3 inflammasome signaling cascade. Our research identified the exosomal transfer of pro-pyroptotic microRNAs from doxorubicin-treated breast cancer cells (BCCs) to cardiomyocytes as a previously unrecognized molecular mechanism that exacerbates anthracycline-induced cardiotoxicity. These translational findings provide new insights into the complex pathophysiological mechanisms connecting malignant tumors and cardiovascular comorbidities, potentially opening promising avenues for targeted therapeutic interventions that could simultaneously address both cancer treatment efficacy and cardioprotection strategies in patients receiving anthracycline-based chemotherapy. The identification of this intercellular communication pathway may lead to the development of novel biomarkers for early detection of cardiotoxicity risk in cancer patients.

## Results

### Coculture with BCCs aggravates DOX-induced pyroptosis in human and mouse cardiomyocytes

**Fig. 1:**
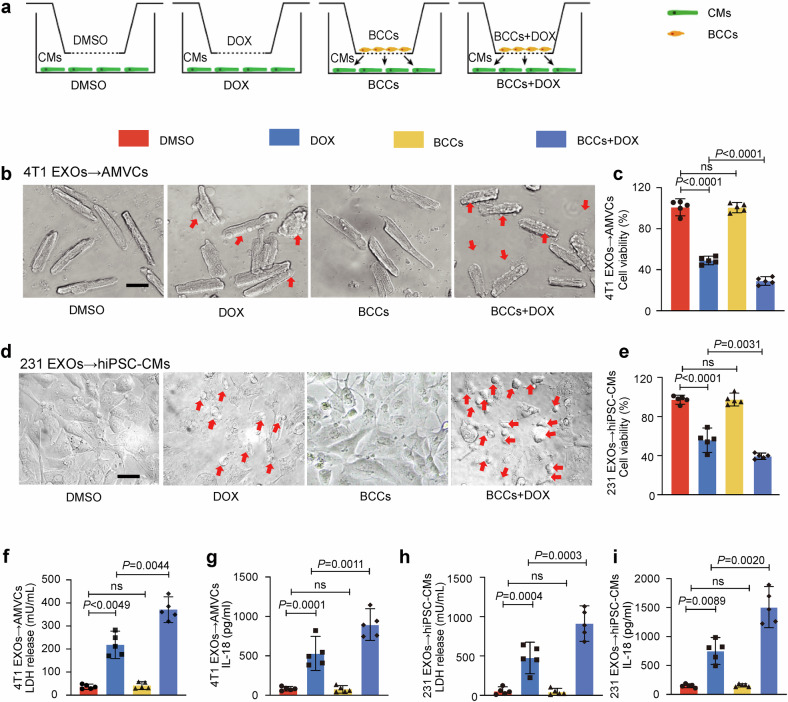
Coincubation with breast cancer cells aggravates DOX-induced pyroptosis in cardiomyocytes. Adult murine ventricular cardiomyocytes (AMVCs) and human induced pluripotent stem cell-derived cardiomyocytes (hiPSC-CMs) were cocultured with breast cancer cells (BCCs) for 24 hours with 1 μM DOX treatment. **a** Schematic diagram of the coculture of BCCs with cardiomyocytes using transwell chambers. **b**–**e** Representative images of and statistics for cell viability after DOX treatment. Scale bar, 50 μm.; (**f**, **g**) lactate dehydrogenase (LDH) release and IL-18 levels were assessed using colorimetric methods in AMVCs (*n* = 5) and in (**h** and **i**) hiPSC-CMs (*n* = 5) (one-way ANOVA was used to compare all experimental groups to the DMSO group). “BCCs” indicates breast cancer cells, Directional arrows indicate exosome transfer between donor cells (4T1 or MDA-MB-231) and recipient cells (hiPSC-CMs or AMVCs). “N-BCC-EXOs” denotes normal breast cancer cell EXOs, and “D-BCC-EXOs” denotes DOX-induced breast cancer cell EXOs. “ns” indicates non-significant, and “DOX” indicates doxorubicin. Data are presented as Mean ± SD

To investigate whether the presence of BCCs can affect DOXIC, coculture experiments with transwell systems were performed as depicted in Fig. 1a. Adult murine ventricular cardiomyocytes (AMVCs) or human induced pluripotent stem cell-derived cardiomyocytes (hiPSC-CMs) were cocultured with the murine BCC cell line 4T1 or human BCC cell line MDA-MB-231, respectively, and treated with 1 μM DOX for 24 hours (Fig. 1a). The results revealed that both AMVCs and hiPSC-CMs experienced more severe cell injury in the presence of BCCs, as evidenced by a more pronounced decrease in cell viability, LDH release and IL-18 in response to DOX treatment (Fig. 1b–e, f–i), suggesting the possibility that BCC–cardiomyocyte communication aggravated DOXIC. Notably, cardiomyocytes cocultured with DOX presented typical morphological characteristics of pyroptosis, including the formation of bubble-like protrusions and cell swelling^23^ (Fig. 1b, d). Immunofluorescence-based visualization of ASC speckling, a hallmark of inflammasome activation in pyroptosis, validated the overactivation of pyroptosis in DOX-treated hiPSC-CMs cocultured with MDA-MB-231 cells (Supplementary Fig. 1a, b). Furthermore, to determine whether EXO-mediated cell crosstalk is involved in this process, we used the EXO release inhibitor GW4869 and the stimulant monensin^24^ to control EXO release. Nanoparticle tracking analysis (NTA) revealed that GW4869 (10 μM, 24 hours) effectively reduced EXOs release, allowing the further application of GW4869 in functional studies (Supplementary Fig. 2). To establish the baseline effects of these modulators, we first verified that neither GW4869 nor monensin affected cell viability, LDH release, or pyroptosis-related protein expression under DMSO treatment (Supplementary Fig. 3a). The results revealed that the coculture-induced upregulation of pyroptosis-related protein (GSDMD-N-terminal, cleaved CASP1, and cleaved IL-1β) expression in DOX-treated AMVCs was significantly attenuated by GW4869 but aggravated by monensin (Supplementary Fig. 3a). In addition, the release of mature IL-18, a hallmark of pyroptosis induction, was examined by ELISA. The IL-18 levels and the level of cardiomyocyte damage exhibited trends similar to those shown in the Western blot results (Supplementary Fig. 3b–d). Taken together, these results suggest that coculture with BCCs aggravates DOX-induced cardiomyocyte pyroptosis, possibly through the secretion and transfer of EXOs.

### EXOs derived from DOX-induced BCCs (D-BCC-EXOs) aggravate cardiomyocyte pyroptosis

**Fig. 2:**
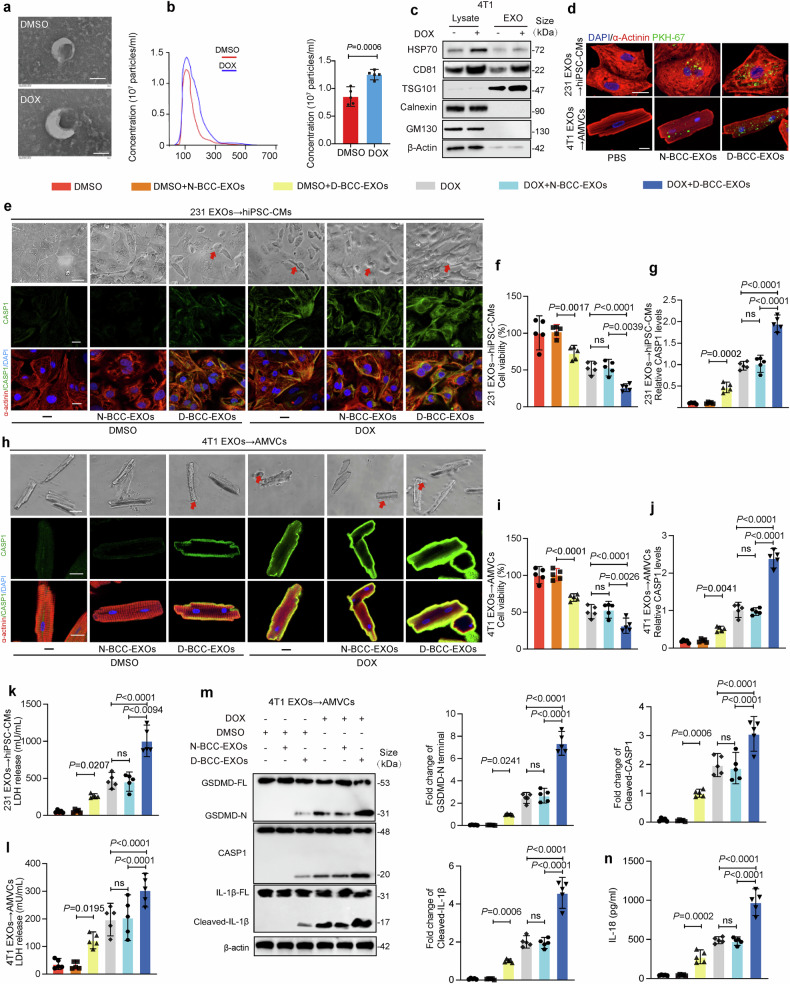
Exosomes from breast cancer cells exacerbate DOX-induced cardiomyocyte pyroptosis in vitro. **a** EXOs from BCCs were characterized using transmission electron microscopy (TEM). Scale bar: 100 nm. **b** Particle size distribution was determined using NanoSight tracking analysis. (NTA). EXO particle concentration in the cell culture supernatant with or without DOX. **c** Western blot analysis of HSP70, CD81, TSG101, GM130, and Calnexin in cell lysate and 4T1-derived EXOs with or without DOX. **d** In vitro and ex vivo breast cancer EXO uptake analysis showing fluorescence-labeled EXOs internalized by human induced pluripotent stem cell-derived cardiomyocytes (hiPSC-CMs) or adult murine ventricular cardiomyocytes (AMVCs) observed under a confocal microscope. For in vitro analysis, PKH67-labeled EXOs were harvested and subsequently incubated with hiPSC-CMs. For ex vivo analysis, AMVCs were isolated from mouse hearts 24 hours after tail vein injection of PKH67-labeled EXOs. Scale bar: 20 μm. **e**, **f** Representative images of AMVCs and hiPSC-CMs after DOX + N-BCC-EXO or DOX + D-BCC-EXO treatment for 24 hours and (**g**, **h**) assessments of cell viability, (**i**, **j**) CASP1 expression levels, and (**k**, **l**) LDH release levels (*n* = 5) are shown. Top row: bright-field images, scale bar: 50μm; middle row: CASP1 immunofluorescence (green), scale bar: 20 μm; bottom row: merged images of α-actinin (red), CASP1 (green), and DAPI (blue), scale bar: 20 μm. **m** Pyroptosis-related protein levels in AMVCs were assessed via Western blotting. All the experimental groups were compared with the DOX group via one-way ANOVA. **n** IL-18 levels in AMVCs were determined using a colorimetric method (*n* = 5). “N-BCC-EXOs” denotes normal breast cancer cell EXOs, and “D-BCC-EXOs” denotes DOX-induced breast cancer cell EXOs. Red represents α-actinin, green represents CASP1, and blue represents DAPI. “DOX” indicates doxorubicin, and “ns” indicates non-significant. Data are presented as Mean ± SD

To further investigate the role of D-BCC-EXOs in DOXIC, EXOs were isolated from the culture media of DOX-treated MDA-MB-231 cells and 4T1 cells and purified via several different methods involving centrifugation and ultracentrifugation. The morphology and phenotypes of the isolated particles were subsequently assessed to characterize the EXOs. Transmission electron microscopy (TEM) revealed that the isolated EXOs had a cup-shaped canonical EXO morphology with a double-layered membrane (Fig. 2a). NTA of the EXOs revealed that the diameters of the particles were within the range of 100–200 nm and that the concentration of EXOs increased after DOX stimulation (Fig. 2b). Moreover, Western blot analysis confirmed the expression of EXO marker proteins, including CD81, TSG101, and HSP70, and the absence of the cell-associated proteins GM130 and Calnexin (Fig. 2c). Next, we evaluated the uptake of D-BCC-EXOs. EXOs derived from DOX-treated MDA-MB-231 cells were fluorescently labeled with the membrane dye PKH67 and then incubated with hiPCS-CMs for 12 hours. To assess uptake in vivo, PKH67-labeled EXOs from DOX-treated 4T1 cells were injected *via* the tail vein. Twenty-four hours later, AMVCs were isolated to analyze their ability to take up EXOs via fluorescence microscopy. PKH67 signals were localized in the cytoplasm of both hiPSC-CMs and AMVCs, demonstrating that cardiomyocytes could internalize D-BCC-EXOs both in vitro and ex vivo (Fig. 2d). Notably, the exosome uptake assay indicated that D-BCC-EXOs were more prone to be taken up by both hiPSC-CMs and AMVCs than were N-BCC-EXOs (Fig. 2d). Additionally, DOX-induced breast cancer cell-conditioned medium (D-CM) exhibits similar toxic effects as D-BCC-EXOs, as evidenced by comparable reductions in cell viability and increases in LDH, IL-1β, and IL-18 levels (Fig. S4a–d). We then evaluated the effects of D-BCC-EXOs on DOX-treated cardiomyocytes. Morphological observations revealed that D-BCC-EXOs significantly increased DOX-induced pyroptotic cell death, as indicated by cellular swelling and bubble formation (Fig. 2e). This observation was validated by immunofluorescence staining for Caspase 1 (CASP1), a crucial player in triggering pyroptosis, in both AMVCs and hiPSC-CMs (Fig. 2e, f). The results revealed that D-BCC-EXOs but not EXOs derived from normal control BCCs (N-BCC-EXOs) aggravated DOXIC, as evidenced by a greater decrease in cell viability and more pronounced LDH release (Fig. 2g, h, k and l). Consistent with the results of the coculture experiments, the results of immunofluorescence staining and Western blotting revealed that the levels of pyroptosis-related markers, such as cleaved caspase-1, N-terminal GSDMD, and cleaved IL-1β, were significantly increased in D-BCC-EXOs but not in N-BCC-EXOs (Fig. 2i, j and m). IL-18 secretion exhibited a similar trend (Fig. 2n). These data suggested that D-BCC-EXOs were taken up by cardiomyocytes, thereby increasing DOX-induced cardiomyocyte pyroptosis.

### D-BCC-EXOs aggravate DOX-induced myocardial injury and pyroptosis in vivo

**Fig. 3:**
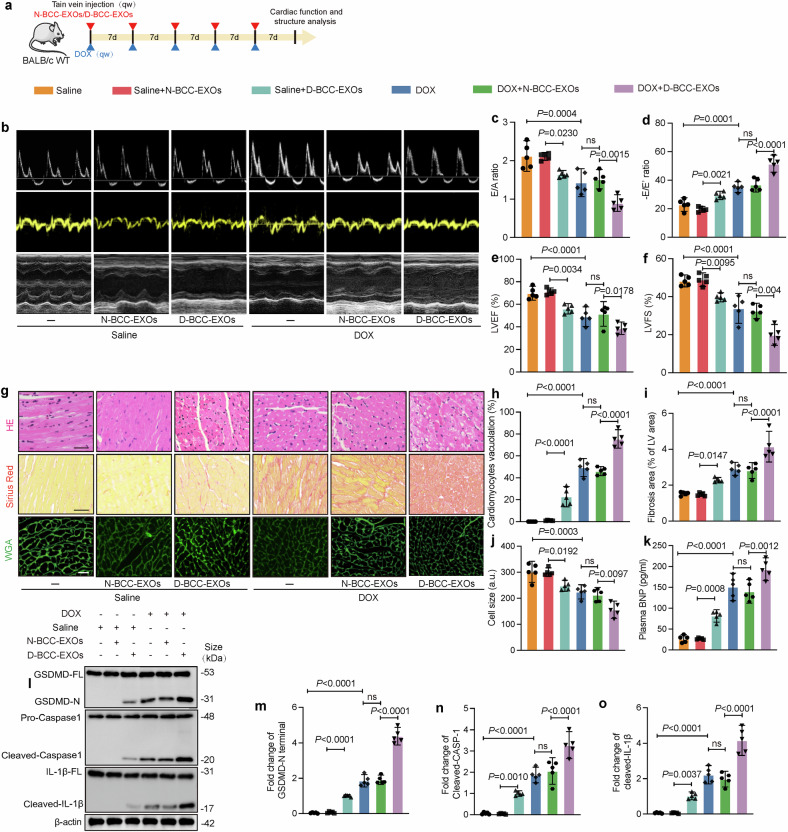
Exosomes from breast cancer cells exacerbate DOX-induced cardiomyocyte pyroptosis in vivo. **a** Schematic illustration of the subchronic DOX exposure model in mice to test the injury efficacy of D-BCC-EXOs (*n* = 5). **b** Representative images of M-mode echocardiography and blood flow velocity (obtained using a Doppler system) in mice. **c**, **d** The E/A and the E/E’ ratios were quantified using the Doppler echocardiography. **e**, **f** Quantification of the left ventricular (LV) ejection fraction and LV fractional shortening via M-mode echocardiography (*n* = 5). **g** Representative images of hematoxylin-eosin (H&E) staining (upper), Sirius red staining indicating myocardial fibrosis (middle), and wheat germ agglutinin (WGA) immunofluorescence staining (lower). Scale bar: 50 μm. **h** Statistics of vacuolization in ventricular tissues. **i** The fibrotic area per left ventricle was quantified via Sirius red staining (*n* = 5). **j** Cell size was quantified via WGA immunofluorescence staining (*n* = 5). **k** Mouse plasma brain natriuretic peptide (BNP) was measured using a colorimetric method (*n* = 5). **l**–**o** Pyroptosis-related protein levels in mouse ventricular tissue were assessed via western blotting (*n* = 5) (one-way ANOVA was used to compare all experimental groups to the DOX group). “E” indicates early diastolic transmitral flow velocity, “E′” indicates early diastolic mitral annular velocity, and “A” indicates late (atrial) diastolic transmitral flow velocity. D-BCC-EXOs, denotes doxorubicin-treated breast cancer EXOs, “N-BCC-EXOs” denotes normal breast cancer cell EXOs, “D-BCC-EXOs” denotes DOX-induced breast cancer cell EXOs, “ns” indicates non-significant, and “DOX” indicates doxorubicin. Data are presented as means ± SD

To determine whether D-BCC-EXOs play a role in DOX-induced myocardial injury in vivo, a subchronic DOXIC mouse model was constructed, and EXOs derived from DOX-treated 4T1 cells (D-BCC-EXOs) or EXOs derived from normal control 4T1 cells (N-BCC-EXOs) were delivered to the mouse heart *via* tail vein injection (Fig. 3a). Echocardiographic studies demonstrated that D-BCC-EXOs but not N-BCC-EXOs significantly aggravated the DOX-induced reductions in the left ventricular ejection fraction (LVEF), left ventricular fractional shortening (LVES), early diastolic-to-atrial (E/A) ratio, and early diastolic-to-early diastolic (E/Eʹ) ratio (Fig. 3b–f). Hematoxylin and eosin (H&E) staining revealed that D-BCC-EXO treatment increased DOX-induced vacuolization of ventricular tissue (Fig. 3g, h). Sirius red staining demonstrated that D-BCC-EXOs increased DOX-induced cardiac fibrosis (Fig. 3g, i). Wheat germ agglutinin (WGA) staining revealed that D-BCC-EXOs aggravated DOX-induced myocardial atrophy (Fig. 3g, j). The level of plasma brain natriuretic peptide (BNP), an indicator of heart failure, was also significantly increased by D-BCC-EXOs. Notably, while N-BCC-EXOs did not affect cardiac function or cause histologic changes in saline-treated mice, D-BCC-EXOs induced cardiac functional impairment and injury in these mice, as revealed by echocardiography and histological analysis. Additionally, the expression of the pyroptosis-related markers N-terminal GSDMD, cleaved caspase-1, and cleaved IL-1β was significantly increased by D-BCC-EXOs (Fig. 3l–o).

To further explore whether breast cancer tissues (BCTs) participate in the aggravation of DOX-induced cardiac injury by the secretion of EXOs into the circulation, we constructed tumor cells and tissues in which EXO release was blocked. EXO secretion is regulated at different stages by Rab27a and Rab27b.^25^ To determine which molecules are responsible for EXO release from BCCs, we first analyzed data from public databases, including the GEPIA (http://gepia.cancer-pku.cn/index.html) and Proteomic Data Commons (https://proteomic.datacommons.cancer.gov/pdc/) databases. We found that the transcript level of Rab27b but not Rab27a was increased in tumor tissues compared with adjacent normal tissues, whereas the protein levels of both Rab27a and Rab27b were increased in tumor tissues (Supplementary Fig. 5a–d). Next, we performed qPCR to measure Rab27a and Rab27b transcript levels in DOX-treated murine BCTs and found that the expression of Rab27a mRNA was greater than that of Rab27b mRNA in BCTs (Supplementary Fig. 5e). Additionally, we found that the mRNA expression of Rab27a mRNA was greater than that of Rab27b in BCTs, suggesting that Rab27a may play an important role in exosome release in BCTs (Supplementary Fig. 5f). EXOs were subsequently isolated from the culture media of 4T1 cells after transfection of a Rab27a knockdown plasmid. Rab27a mRNA expression was then measured via qRT‒PCR (Supplementary Fig. 5g), and the generated EXOs were analyzed via electron microscopy (Supplementary Fig. 5h). The levels of the EXO marker proteins HSP70, CD81, and TSG101 in Rab27a 4T1 cells with or without Rab27a knockdown were measured by Western blotting (Supplementary Fig. 5j). Measurement of the EXO concentration revealed that EXO release was significantly reduced in 4T1 cells in which Rab27a was silenced compared with 4T1 cells transfected with the control vector (Supplementary Fig. 5i, k).

These findings suggest that EXO secretion by 4T1 cells may be effectively suppressed by Rab27a silencing. Subsequently, in situ breast cancer mouse models were employed, and a lentivirus expressing an shRNA targeting Rab27a (shRab27a) was intratumorally injected (Supplementary Fig. 6a). shRab27a did not affect tumor growth, as indicated by tumor volume measurements (Supplementary Fig. 6b). The DOX-induced reductions in the E/A ratio, E/E’ ratio, LVEF, and LVFS were reversed (Supplementary Fig. 6c–f), myocardial vacuolization and atrophy were inhibited (Supplementary Fig. 6g–j), and the plasma level of BNP after DOX treatment (Supplementary Fig. 6k) in shRab27a-injected mice compared with negative control shRNA (shNC)-injected mice. Rab27a knockdown did not significantly alter cardiac functional parameters or myocardial morphology under saline treatment. These data suggested that circulating EXOs from DOX-treated BCTs aggravated DOX-induced myocardial injury.

### miR-216a-5p is the candidate effector of D-BCC-EXOs to aggravate DOXIC

**Fig. 4:**
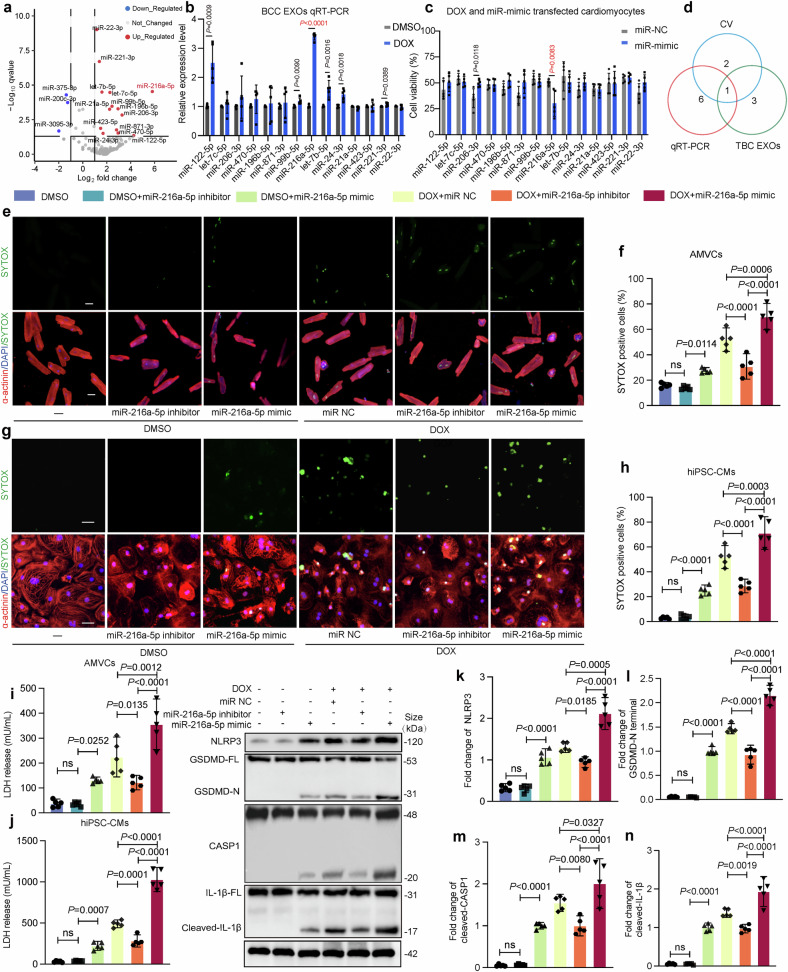
Exosomal miR-216a-5p upregulation induced by DOX aggravates DOX-mediated cardiomyocyte pyroptosis. **a** Sequencing data of mouse plasma EXO miRNAs were used to identify miRNAs that were differentially expressed. The differentially expressed miRNAs are depicted in a volcano plot. **b** 4T1 cells were treated with DOX or DMSO, and upregulated exosomal miRNAs were identified by qRT‒PCR (*n* = 5). **c** Adult murine ventricular cardiomyocytes (AMVCs) were transfected with a miR-216a-5p mimic/scramble before being treated with DOX. Cell viability was determined by CCK-8 assays (*n* = 5). **d** Venn analysis revealed that miR-216a-5p satisfied two conditions. **e**–**h** miR-216a-5p knockdown and overexpression in cardiomyocytes were achieved through transfection with miR-216a-5p inhibitors and mimics, respectively. Multiple immunofluorescence stains and statistics were used to quantify AMVC and human induced pluripotent stem cell-derived cardiomyocytes (hiPSC-CM) death. DAPI (blue), SYTOX (green), and α-actinin (red) (*n* = 5). Scale bar: 50 μm. **i**, **j** LDH release levels and cell viability were measured (*n* = 5). **k**–**n** The protein expression levels of NLRP3, GSDMD-N, cleaved CASP1, and cleaved IL-1β were detected via Western blotting (*n* = 5) (one-way ANOVA was used to compare all experimental groups with the DOX+miR NC group). “BCCs” indicates breast cancer cells, “ns” indicates non-significant, and “DOX” indicates doxorubicin. Data are presented as means ± SD

Studies have shown that EXOs mediate cellular communication primarily through the exchange of miRNAs or proteins between cells.^26^ Therefore, we investigated whether the miRNAs carried by D-BCC-EXOs contributed to their ability to aggravate DOXIC. To this end, we first investigated the effects of EXOs from Dicer-knockout (CRISPR deletion) 4T1 cells in which all miRNAs were depleted. To validate the Dicer knockout efficiency, we examined Dicer mRNA expression level in 4T1 cells and found that it was significantly decreased in knockout cells compared to control cells (Supplementary Fig. 7a). D-BCC-EXOs induced cardiomyocyte injury both under saline and DOX treatment, while these damaging effects were blocked by Dicer deficiency (Supplementary Fig. 7b–e). These results suggest that the key molecules mediating the effects of D-BCC-EXOs could be miRNAs. To explore and identify differentially expressed miRNAs in plasma EXOs from DOX-treated and saline-treated breast cancer-bearing mice, exosomal miRNA-seq was conducted. The results revealed that 14 miRNAs were significantly upregulated, and 3 were downregulated (fold change ≥ 2, q < 0.05) (Fig. 4a). The detailed sequencing results are presented in Supplementary Table 1 and 2. To identify functional miRNAs among the 14 upregulated miRNAs that aggravated DOXIC, we followed three steps. First, after treating 4T1 cells with DOX and extracting EXOs, we assessed the expression levels of the 14 miRNAs in EXOs via qRT‒PCR. We identified five miRNAs that were significantly upregulated, namely, miR-216a-5p, miR-99b-5p, miR-122-5p, miR-206-3p, and miR-423-5p (Fig. 4b). Second, miRNA mimics of the 14 candidate miRNAs were transfected into AMVCs, and cell viability after DOX treatment was assessed. Two of the identified miRNAs—miR-216a-5p and miR-221-3p mimics—significantly reduced cell viability (Fig. 4c). Third, the 14 identified miRNAs were verified in EXOs isolated from BCTs in mice via qRT‒PCR (Supplementary Fig. 7a). Only miR-216a-5p met the three requirements according to a Venn analysis (Fig. 4d) and was confirmed by qRT-PCR in mouse plasma EXOs (Supplementary Fig. 7a, b). Furthermore, following D-BCC-EXO treatment, the expression level of miR-216a-5p was elevated in mouse cardiac tissue and AMVCs. Moreover, N-BCC-EXOs had no effect on miR-216a-5p expression in either the DOX or DMSO group. The level of endogenous primary miR-216a-5p (pri-miR-216a), the precursor of miR-216a-5p, remained almost unchanged (Supplementary Fig. 9a–d). Cardiomyocytes were transfected with an miR-216a-5p mimic, inhibitor, or control. To further establish the causal relationship between miR-216a-5p and DOXIC, we performed both loss- and gain-of-function studies in cardiomyocytes. Specifically, we achieved miR-216a-5p knockdown and overexpression through transfection with miR-216a-5p inhibitors and mimics, respectively. Our results demonstrated that miR-216a-5p overexpression significantly aggravated the DOX-induced death of both hiPSC-CMs and AMVCs, whereas miR-216a-5p knockdown had the opposite effect (Fig. 4e–j). Consistently, Western blot analysis revealed similar changes in the levels of pyroptosis-related proteins in AMVCs (Fig. 4k–n). These results indicate that miR-216a-5p is a key component of D-BCC-EXOs that exacerbates DOXIC.

To clarify whether the increase in cardiac miR-216a-5p levels is a result of miR-216a-5p of breast cancer origin and whether cardiac miR-216a-5p is transmitted to recipient heart/cardiomyocytes in an EXO-dependent manner, we performed several experiments. First, we performed miRNA sequencing to examine alterations in miRNA profiles in the blood and myocardium of DOX-treated breast cancer-bearing mice intratumorally injected with a lentivirus expressing shRNA targeting Rab27a (ShRab27a) or negative control shRNA (shNC). The results revealed that 34 miRNAs were upregulated, 54 miRNAs were downregulated in the murine blood, and 9 miRNAs were upregulated, and 20 miRNAs were downregulated in the murine myocardium (Supplementary Fig. 10a, b, Supplementary Tables 3-6). Notably, miR-216a-5p was among the top 10 downregulated miRNAs in shRab27a-injected mice according to sequencing data from both blood and myocardial tissues (Supplementary Fig. 10a, b). We intersected the 54 miRNAs downregulated in the blood of shRab27a-injected mice, 20 miRNAs downregulated in the myocardium of shRab27a-injected mice with 14 miRNAs upregulated in plasmic EXOs from DOX-treated breast cancer-bearing mice and found that miR-216a-5p was the only dysregulated miRNA in all three groups (Supplementary Fig. 10c). Second, we used qPCR to examine the levels of miR-216a-5p in breast cancer-bearing mice and mice without tumors. The results revealed that miR-216a-5p was consistently upregulated by DOX in the plasma, plasma EXOs, and myocardial tissues of tumor-bearing mice but not in those of mice without tumors or tumor-bearing mice subjected to intratumoral injection of the lentivirus expressing shRab27a (Supplementary Fig. 10d-f). Third, we also examined the levels of miR-216a-5p in DOX-treated mice without tumors that had been intravenously injected with N-BCC-EXOs, D-BCC-EXOs, or D-BCC-EXOs but not DOX-treated mice without tumors that had been N-BCC-EXOs, and the results revealed obviously increased levels of miR-216a-5p in plasma, plasma EXOs, and cardiac tissues (Supplementary Fig. 10g-i). Notably, D-BCC-EXOs failed to increase pri-miR-216a levels in the myocardium, ruling out the possibility of endogenous pri-miR-216a transcription in the heart (Supplementary Fig. 10j). Finally, we intersected the 54 miRNAs downregulated in the blood of shRab27a-injected mice with the 14 miRNAs upregulated in the plasma EXOs of DOX-treated breast tumor-bearing mice and found that 2 common miRNAs, including miR-216a-5p, were dysregulated under both conditions. We next performed qPCR to validate the changes in the expression of human orthologous miRNAs in breast cancer patients treated with DOX. The results revealed that miR-216a-5p was the only miRNA whose expression was increased in the blood of DOX-treated patients with breast cancer (Supplementary Fig 11a, b). These results collectively demonstrated that donor BCC/BCT-derived miR-216a-5p could be transferred to recipient cardiomyocytes/hearts via an EXO-dependent pathway for remote intercellular/interorgan communication.

To determine the concentration threshold at which miR-216a-5p affects the function of heart muscle, we measured the plasma level of miR-216a-5p and the plasma cTnI concentration in 36 patients who had been diagnosed with breast cancer and suffered from DOXIC. In addition, cardiac function was assessed by echocardiography, and the LVEF was recorded before beginning DOX treatment and re-evaluated at 2 weeks, 1 month, 3 months, and 6 months after completion of DOX treatment. The maximal reduction in the LVEF before and after DOX treatment was calculated as the ΔLVEF%. We performed a correlation analysis and identified significant correlations between the plasma level of miR-216a-5p and the cTnI concentration (*r* = 0.6341, *p* < 0.0001), as well as between the plasma level of miR-216a-5p and the ΔLVEF% (*r* = −0.7068, *p* < 0.0001). From the correlation graphs, we can infer that patients with a 2-fold increase in plasma miR-216a-5p levels experienced an ~8% reduction in the LVEF and a reduction in the cTnI concentration of approximately 0.4 ng/ml (Supplementary Fig 12a, b).

### DOX upregulates ATF3 to promote miR-216a-5p transcription

**Fig. 5:**
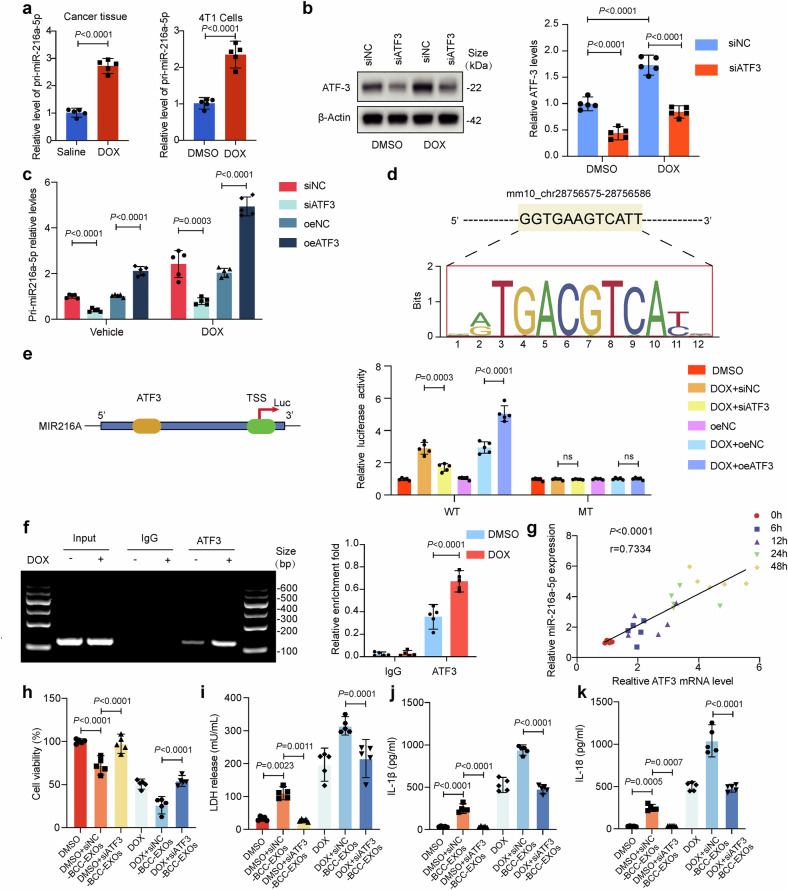
DOX upregulates ATF3 to promote miR-216a-5p transcription in BCCs. **a** Relative pri-miR-216a expression in mouse cancer tissue and 4T1 cells from DMSO- and DOX-induced mice was analyzed via qRT-PCR. **b** Protein levels were assessed by Western blotting using DOX/DMSO-treated 4T1 cells with or without siATF3. **c** The relative pri-miR-216a expression in DOX/DMSO-treated 4T1 cells with ATF3 overexpression or knockdown was analyzed via qRT-PCR. **d**, **e** The direct effects of miR-216a-5p on mouse ATF3 were identified using a luciferase assay. 4T1 cells were cotransfected with siATF3/oeATF3 and reporter plasmids for 48 hours. Firefly/Renilla luciferase activity was used to evaluate regulatory effects. In each group, the fold change was calculated by dividing the firefly/Renilla luciferase activity by the value obtained for the DMSO group. **f** ChIP-PCR was performed using an anti-ATF3 antibody or IgG in 4T1 cells, and PCR of the miR-216a promoter was performed. The values correspond to the ratio of the anti-ATF3 immunoprecipitated DNA relative to the IgG immunoprecipitated DNA. **g** ATF3 and miR-216a-5p expression levels gradually increased in a time-dependent manner after DOX treatment, and the two indicators were positively correlated. **h** Adult murine ventricular cardiomyocytes (AMVCs) cell viability was determined using CCK-8 assays (*n* = 5). **i**–**k** LDH release and IL-18 and IL-1β levels in AMVCs were assessed using a colorimetric method (*n* = 5). “siNC-BCC-EXOs” denotes exosomes from control siRNA-transfected DOX-induced 4T1 cells, “siATF3-BCC-EXOs” denotes exosomes from ATF3-knockdown DOX-induced 4T1 cells, and “DOX” indicates doxorubicin. Data are presented as means ± SD

To determine the mechanism underlying the upregulation of miR-216a-5p expression in DOX-treated BCCs, we focused on identifying the transcriptional regulators that initiate the transcription of miR-216a since pri-miR-216a expression was also upregulated in DOX-treated 4T1 cells and mouse BCTs (Fig. 5a). Bioinformatics prediction revealed that the promoter region of pri-miR-216a contains binding sites for ATF3 (Supplementary Fig. 13), which has been revealed to be a master regulator of the response to DOX in breast cancer.^27,28^ Consistent with previous reports, ATF3 expression was significantly increased in DOX-treated 4T1 cells, and an siRNA construct targeting ATF3 successfully knocked down ATF3 (Fig. 5b). Next, we silenced or overexpressed (oe) ATF3 to determine whether ATF3 affects pri-miR-216a expression. As shown in Fig. 5c, the level of pri-miR-216a was increased by ATF3. Bioinformatics prediction (using the JASPAR database) revealed that the promoter region of pri-miR-216 contains a site (mm10_chr11:28,756,575-28,756,586+) with a high binding score for the transcription factor ATF3 (Fig. 5d). To explore the regulatory relationship between ATF3 and pri-miR-216a, we constructed luciferase reporter vectors containing the wild-type (WT) or a mutant (MT) full-length pri-miR-216a promoter to determine whether ATF3 regulates pri-miR-216a transcription (Fig. 5e). The results demonstrated that DOX treatment or ATF3 overexpression significantly increased WT reporter luciferase activity in 4T1 cells, whereas ATF3 knockdown attenuated the DOX-induced increase in WT reporter luciferase activity. Moreover, the luciferase activity of the MT reporter (in which the ATF3 binding site was mutated) was not altered by DOX or ATF3 (Fig. 5e).

Additionally, we performed a chromatin immunoprecipitation‒PCR (ChIP‒PCR) assay with an anti-ATF3 antibody to further confirm that ATF3 binds to the miR-216a promoter. As shown in Fig. 5f, the miR-216a promoter region was significantly enriched with ATF3, not with IgG, and this enrichment was markedly enhanced by DOX treatment, indicating that ATF3 can directly bind to the promoter region of miR-216a and promote its transcription and that this effect can be strengthened by DOX treatment. Additionally, we observed a positive correlation between miR-216a-5p expression and ATF3 expression in 4T1 cells treated with DOX at different time points, further supporting the transactivation of miR-216a by ATF3 in D-BCC-EXOs (Fig. 5g).

To further evaluate the functional significance of ATF3 in the pathological communication between BCCs and cardiomyocytes in DOXIC, we silenced ATF3 in DOX-induced 4T1 cells and examined the effects of their derived EXOs. The AMVCs were treated with EXOs from negative control or ATF3-knockdown DOX-induced 4T1 cells (siNC-BCC-EXOs or siATF3-BCC-EXOs, respectively), with or without DOX exposure. Both in DOX and DMSO-treated conditions, siNC-BCC-EXOs significantly impaired AMVC function, as evidenced by decreased cell viability (Fig. 5h), increased LDH release (Fig. 5i), and elevated IL-1β and IL-18 secretion (Fig. 5j, k). However, ATF3 knockdown markedly attenuated these detrimental effects. These findings strongly suggest that ATF3 plays a crucial role in mediating the pathological communication between BCCs and cardiomyocytes during DOXIC.

### SF3B4 selectively packages miR-216a-5p into BCC-EXOs

**Fig. 6:**
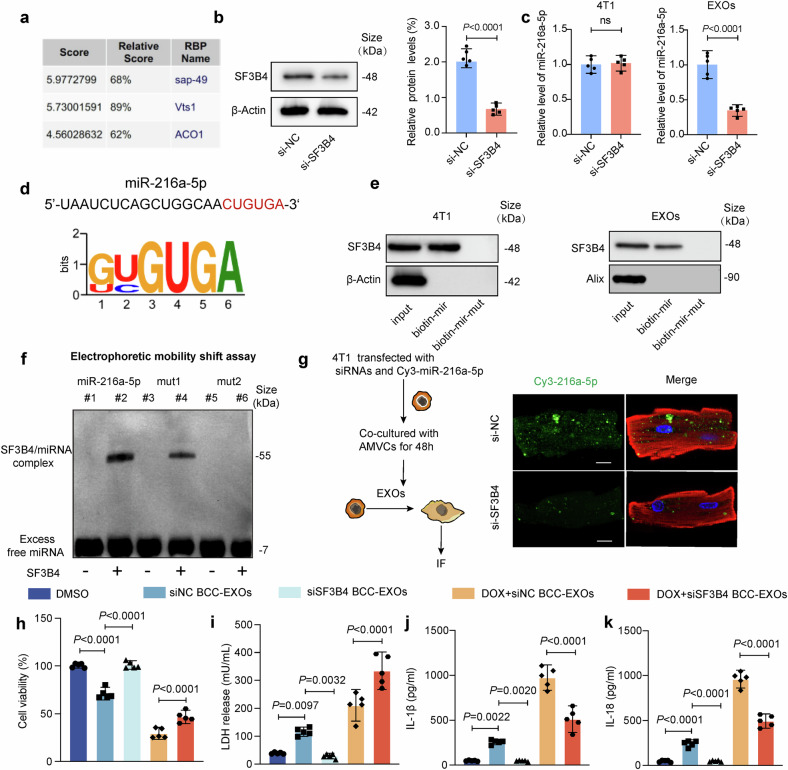
SF3B4 (sap-49) packages miR-216a-5p into 4T1 exosomes. **a** Three candidate proteins were screened from RBPBD. **b** The effect of SF3B4 knockdown in 4T1 cells was assessed via western blotting. **c** The miR-216a-5p level in cell lysates and EXOs was analyzed via qRT‒PCR. **d** The SF3B4 binding motif and the matched miR-216a-5p sequence. **e** The binding of the wild-type and mutated miR-216a-5p probes to SF3B4 in 4T1 cells and EXOs from 4T1 cells was measured using an RNA pull-down assay. **f** The identified motif was the genuine binding region of miR-216a-5p and SF3B4, and mutated versions of miR-216a-5p were tested for binding to SF3B4 via EMSA. **g** SF3B4 in 4T1 cells was knocked down, and EXOs were isolated. After adult murine ventricular cardiomyocytes (AMVCs) were incubated with 4T1-derived EXOs containing Cy3-miR-216a-5p, Cy3 fluorescence in AMVCs was detected. Scale bar: 20 μm. **h** AMVCs were treated with SF3B4-knockdown and DOX-exposed 4T1 BCC-EXOs and exposed to DOX. The viability of AMVCs was assessed using CCK-8 assays (*n* = 5). **i**–**k** LDH release and IL-18 and IL-1β levels in AMVCs were assessed using a colorimetric method (*n* = 5). “siNC-BCC-EXOs” indicates exosomes from control siRNA-transfected DOX-induced 4T1 cells, “siSF3B4-BCC-EXOs” denotes exosomes from SF3B4-knockdown DOX-induced 4T1 cells, “BCCs” denotes breast cancer cells, “ns” indicates non-significant, and “DOX” indicates doxorubicin. Data are presented as means ± SD

Recently, RNA-binding proteins (RBPs) have emerged as crucial determinants of the selective sorting and packaging of transcripts into EXOs.^29^ We investigated the mechanisms underlying the RBP-mediated sorting and packaging of miR-216a-5p in BCC-derived EXOs. The specific interaction between the miR-216a-5p sequence and RBP motifs was analyzed using the online prediction website RBPBD (threshold > 0.6).^30^ The results revealed that spliceosome-associated protein 49 (SAP49, gene symbol: SF3B4), VTS1 protein (*S. cerevisiae*, not present in humans or mice), and aconitase 1 (ACO1) have specific miR-216a-5p binding sites (Fig. 6a). Further research revealed that knockdown of SF3B4 but not ACO1 in 4T1 cells by specific siRNAs significantly reduced EXO miR-216a-5p levels, whereas intracellular miR-216a-5p levels remained almost unchanged (Supplementary Figs. 14 and 6, c), suggesting that SF3B4 has regulatory effects on miR-216a-5p levels in EXOs. The SF3B4 binding motif and the matched miR-216a-5p sequence are shown in Fig. 6d. We then performed an RNA pull-down assay to determine whether SF3B4 could bind to miR-216a-5p. The results demonstrated that the WT miR-216a-5p probe was able to effectively capture SF3B4 in both 4T1 cells and EXOs derived from 4T1 cells. However, the binding ability of the miR-216a-5p probe was abolished when the “CUGUGA” sequence of miR-216a-5p was mutated (Fig. 6e). To confirm that this motif was in fact the binding site between miR-216a-5p and SF3B4, the ability of mutated versions of miR-216a-5p to bind to SF3B4 was assessed via an electrophoretic mobility shift assay (EMSA). The first MT (miR-216a-5p-mut1) was only a partial MT, as bases 2 to 6 of the motif were preserved, while the first base was changed from C to A. The second MT (miR-216a-5p-mut2) was a complete MT, as the purine and pyrimidine residues throughout the motif were changed (Fig. 6f). RNA EMSA revealed that WT miR-216a-5p and the partial MT miR-216a-5p-mut1 bound to SF3B4, whereas miR-216a-5p-mut2 did not. These results further confirmed that specific binding between miR-216a-5p and SF3B4 was essential for the ability of miR-216a-5p to be packaged into BCC-derived EXOs (Fig. 6f). Furthermore, we knocked down SF3B4 in 4T1 cells via Cy3-miR-216a-5p transfection and isolated EXOs. After AMVCs were incubated with 4T1-derived EXOs expressing Cy3-miR-216a-5p, a Cy3 signal was observed in the AMVCs, whereas SF3B4 knockdown led to a decrease in the Cy3 fluorescence intensity in AMVCs, indicating that SF3B4 knockdown reduced the transport of exosomal miR-216a-5p from 4T1 cells to AMVCs (Fig. 6g).

To evaluate the functional significance of SF3B4 in pathological communication between BCCs and cardiomyocytes in DOXIC, we silenced SF3B4 in 4T1 cells and isolated EXOs before DOX treatment. Our results revealed that DOX-treated BCC-derived EXOs (siNC-BCC-EXOs) significantly decreased AMVC cell viability (Fig. 6h), increased LDH release (Fig. 6i), and increased the secretion of the pyroptosis markers IL-1β and IL-18 (Fig. 6j, k), whereas EXOs derived from SF3B4 knockdown BCCs (siSF3B4-BCC-EXOs) attenuated these detrimental effects. Moreover, when cardiomyocytes were treated with DOX, EXO-mediated cellular injury was further exacerbated, but SF3B4 silencing protected against these changes (Fig. 6h–k). These data further corroborated the pivotal role of SF3B4 in transporting miR-216a-5p from BCCs to cardiomyocytes in DOXIC.

### miR-216a-5p aggravates DOXIC by targeting ITCH

**Fig. 7:**
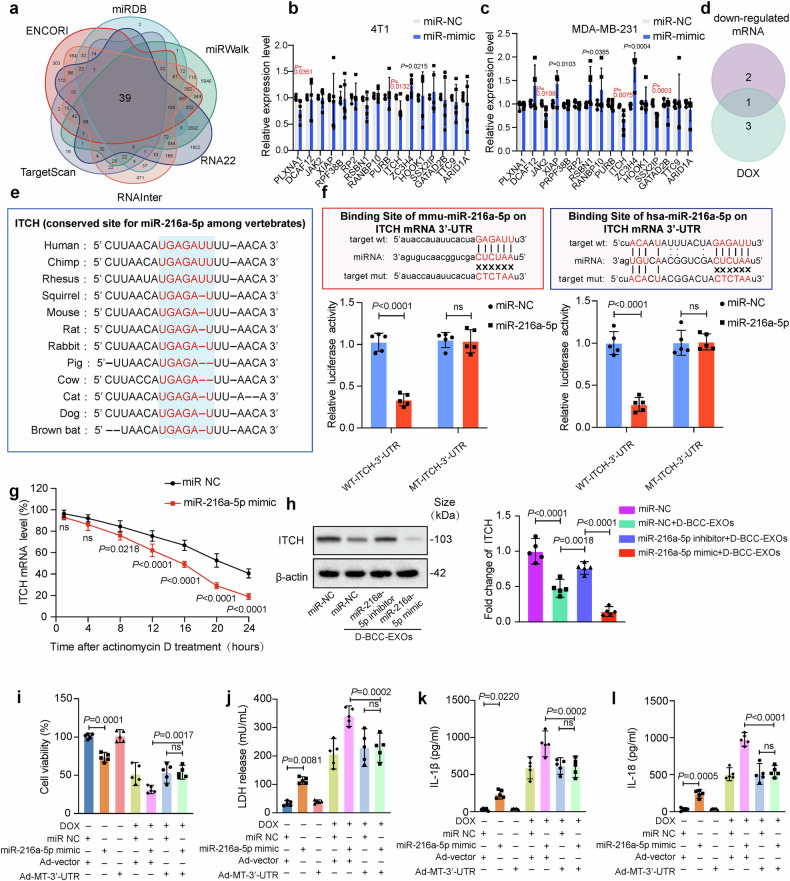
miR-216a-5p aggravates DOX-mediated cardiomyocyte injury by targeting ITCH. **a** A total of 39 human miR-216a-5p targets were identified via the intersection of six microRNA target prediction databases. **b** miR-216a-5p mimic-transfected 4T1 cells were cocultured with adult murine ventricular cardiomyocytes (AMVCs) and exposed to DOX, after which, downregulated mRNAs were identified via qRT‒PCR (*n* = 5). **c** miR-216a-5p mimic-transfected MDA-MB‒231 cells were cocultured with hiPSC‒CMs and exposed to DOX, after which the downregulated mRNAs were identified via qRT‒PCR. **d** Venn analysis was performed, and only ITCH decreased under both conditions. **e** The binding site between ITCH mRNA and the “seed” region of miR-216a-5p is highly conserved among various vertebrates. **f** The binding and regulatory relationships between miR-216a-5p and the 3’-UTRs of ITCH mRNAs in HL-1 and AC16 cells were identified via luciferase reporter plasmid analysis (*n* = 5). The reported plasmids containing the ITCH mRNA 3’-UTR regions (including binding sites) are shown above (mutated binding sites were reversed in sequence). **g** The half-life of ITCH mRNA was measured after actinomycin D treatment in the miR NC and miR-216a-5p mimic groups (*n* = 5). **h** ITCH levels were assessed via western blotting (*n* = 5). **i** AMVCs were transfected with adenovirus expressing ITCH mRNA containing an MT-3’-UTR and transfected with the miR NC/miR-216a-5p mimic before DOX treatment for 24 hours. Cell viability was assessed (*n* = 5). **j**–**l** LDH release and IL-18 and IL-1β levels were analyzed (*n* = 5). “Ad-vector” indicates adenovirus control vector; “Ad-MT-3’-UTR” denotes adenovirus expressing ITCH mRNA containing mutant 3’-untranslated region resistant to microRNA-216a-5p; “ns” indicates non-significant; and “DOX” indicates doxorubicin. Data are presented as means ± SD

To gain further insight into the signaling molecules that mediate the effects of miR-216a-5p, we conducted bioinformatic analyses and then performed experimental validation. A total of 39 targets of human miR-216a-5p were identified from six microRNA target prediction databases (miRDB, miRWalk, RNA22, RNAlnter, TargetScan, and ENCORI) (Fig. 7a and Supplementary Table 7). Given the conserved effects of miR-216a-5p in both humans and mice, we focused on putative targets with conserved miR-216a-5p binding sequences, and a total of 16 conserved targets were found (Supplementary Table 7). We performed two experiments to validate these 16 putative targets as genuine targets of miR-216a-5p in the aggravation of DOXIC. First, miR-216a-5p mimic-transfected 4T1 cells were cocultured with AMVCs and exposed to DOX. Second, miR-216a-5p mimic-transfected MDA-MB-231 cells were cocultured with hiPSC-CMs and exposed to DOX. The mRNA levels of the 16 putative targets were examined via qRT‒PCR under both conditions (Fig. 7b, c). Intersecting the downregulated targets revealed that only ITCH showed a decrease in expression under both conditions (Fig. 7d). Furthermore, the binding site between ITCH mRNA and the “seed” region of miR-216a-5p was found to be highly conserved among various vertebrates (Fig. 7e). Luciferase reporter plasmids carrying the ITCH 3′-untranslated region (UTR) with MT or WT binding sites were used. The results revealed that miR-216a-5p mimic cotransfection suppressed luciferase activity in HL1 cells and AC16 cells expressing WT-ITCH-3′-UTRs but not in HL1 cells or AC16 cells expressing mutant (MT)-ITCH-3′-UTRs (Fig. 7f), which supported the binding and regulatory relationship between miR-216a-5p and the 3′-UTR of ITCH.

miRNAs are known to repress target gene expression by increasing mRNA degradation. To determine whether miR-216a-5p increased ITCH mRNA degradation, the RNA stability of ITCH mRNA was measured in HL1 cells expressing the WT-3′-ITCH-3′-UTR. HL1 cells were treated with a miR-216a-5p mimic. The results revealed that the miR-216a-5p mimic markedly increased ITCH mRNA degradation in HL1 cells treated with the transcription inhibitor actinomycin D (Fig. 7g). Next, we assessed ITCH protein levels in response to the miR-216a-5p mimic in AMVCs. D-BCC-EXO treatment significantly reduced ITCH protein levels, which were further reduced by the miR-216a-5p mimic, and this change was reversed by the miR-216a-5p inhibitor (Fig. 7h).

Furthermore, miR-216a-5p aggravated DOXIC through interaction with the ITCH 3’-UTR. We evaluated the effects of miR-216a-5p overexpression and ITCH 3’-UTR mutation in response to DOX treatment. For this purpose, we employed an adenovirus vector expressing ITCH mRNA with an MT-3’-UTR unable to bind miR-216a-5p (Ad-MT-3’-UTR). Upon DOX treatment, the miR-216a-5p mimic induced a significant decrease in cell viability (Fig. 7i), an increase in LDH release (Fig. 7j), and an increase in the levels of the pyroptosis markers IL-1β and IL-18 (Fig. 7k, l). Importantly, LDH release was increased (Fig. 7J), and the levels of the pyroptosis markers IL-1β and IL-18 were increased (Fig. 7k, l). ITCH 3’-UTR mutation attenuated these deleterious effects.

Taken together, these results demonstrated that miR-216a-5p aggravated DOXIC by binding to and inducing the degradation of ITCH mRNA.

### The miR-216a-5p/ITCH axis aggravates DOXIC by reducing the ubiquitination of TXNIP

**Fig. 8:**
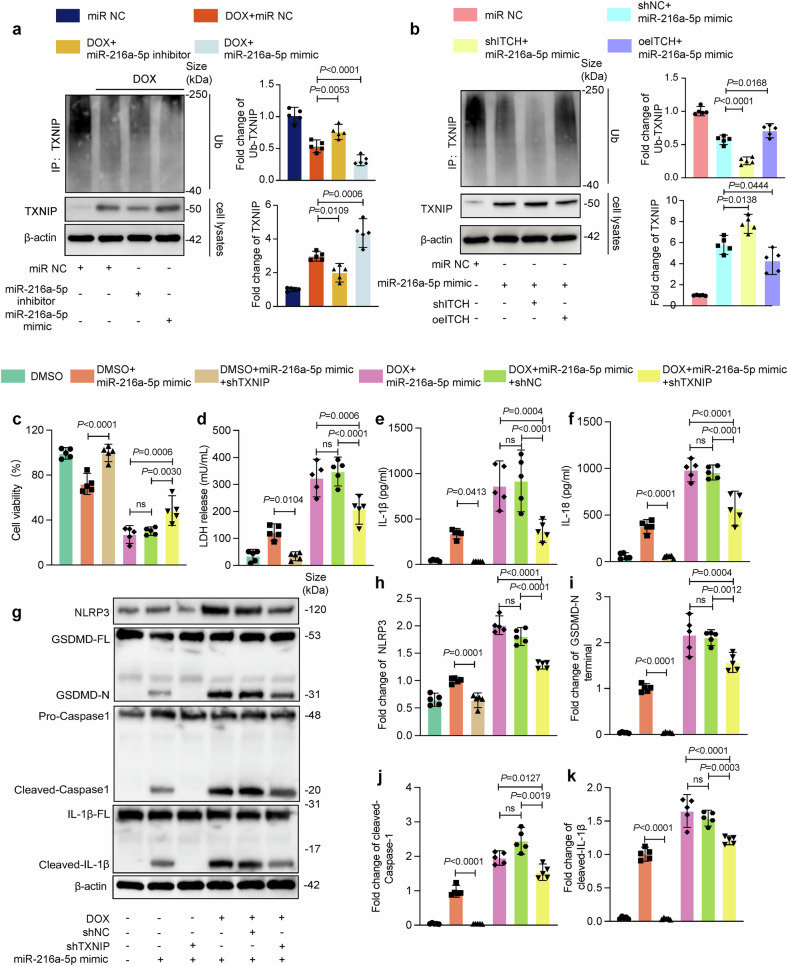
The miR-216a-5p/ITCH axis reduces TXNIP ubiquitination, aggravating DOX-induced cardiomyocyte pyroptosis. To determine whether miR-216a-5p inversely regulates TXNIP ubiquitination to promote TXNIP expression in DOX-treated adult murine ventricular cardiomyocytes (AMVCs), (**a**) TXNIP ubiquitination levels were assessed via Co-IP. AMVCs were transfected with a miR-216a-5p inhibitor or mimic for 24 hours and exposed to DOX or DMSO for another 24 hours. The protein lysates were immunoprecipitated with an anti-TXNIP antibody and immunoblotted with the indicated antibodies. To explore whether this effect could be regulated by ITCH knockdown or overexpression, (**b**) we assessed TXNIP ubiquitination via co-IP after AMVCs were transfected with adenovirus-shITCH or overexpression (oe) ITCH and transfected with the miR-216a-5p mimic. The reverse effect of TXNIP knockdown on DOX-induced pyroptosis was verified by (**c**) cell viability (*n* = 5), (**d**–**f**) LDH release; IL-18 and IL-1β levels; (**g**–**k**) and levels of pyroptosis-related proteins (NLRP3, GSDMD-N, cleaved-caspase-1, and cleaved-IL-1β) (*n* = 5). “ns” indicates non-significant, and “DOX” indicates doxorubicin. Data are presented as means ± SD

ITCH has been identified as an E3 ubiquitin ligase for the ubiquitination and degradation of TXNIP.^31–33^ Additionally, studies have demonstrated that TXNIP promotes pyroptosis by activating the NLRP3-GSDMD inflammasome pathway.^34^ We speculated that the miR-216a-5p/ITCH axis regulates DOXIC through the modulation of TXNIP ubiquitination. To verify this hypothesis, we overexpressed or knocked down TXNIP and ITCH by transfecting AMVCs with adenovirus vectors for 24 hours (Supplementary Fig. 15). We subsequently examined the level of TXNIP ubiquitination and found that miR-216a-5p decreased TXNIP ubiquitination to increase TXNIP expression in DOX-treated AMVCs (Fig. 8a) and that this effect was blocked by ITCH knockdown and aggravated by ITCH overexpression (Fig. 8b). In addition, our cell injury experiments revealed that, compared with the DMSO control, the miR-216a-5p mimic alone significantly decreased AMVC viability and increased LDH release, IL-1β and IL-18 levels, and pyroptosis-related protein expression, while these effects were reversed by TXNIP knockdown under baseline conditions. Similarly, under DOX treatment, TXNIP knockdown reversed the miR-216a-5p-induced decreases in AMVC viability, LDH release, and the release of the pyroptosis markers IL-18 and IL-1β (Fig. 8c–f). Western blot analysis revealed that the levels of pyroptosis-related markers were significantly reduced by TXNIP knockdown under both DMSO and DOX treatment (Fig. 8g–k). These data suggested that the miR-216a-5p/ITCH axis aggravated DOX-induced cardiomyocyte pyroptosis by regulating TXNIP ubiquitination.

### Cardiomyocyte-specific miR-216a-5p sponges attenuate the exacerbation of DOX-induced cardiomyocyte pyroptosis by BCC-derived EXOs in vivo

**Fig. 9:**
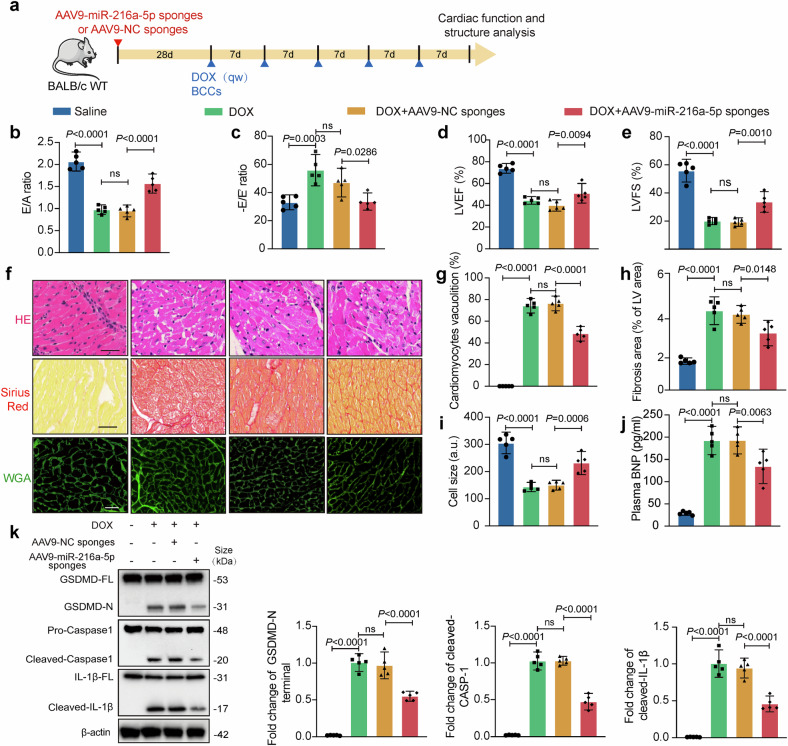
The cardiomyocyte-specific miR-216a-5p sponges reduce the damaging impact of breast cancer EXOs on DOX-induced cardiomyocyte pyroptosis in vivo. **a** miR-216a-5p sponges and negative control (NC) sponges were carried by adeno-associated virus with serotype 9 (AAV9) with the cTnT promoter. Four weeks after the injection, the breast cancer model mice were treated with DOX (*n* = 5). **b**, **c** The E/A and -E/E′ ratios were quantified using the Doppler echocardiography. **d**, **e** Quantification of the left ventricular (LV) ejection fraction and LV fractional shortening via M-mode echocardiography. **f** A representative image of H&E staining, Sirius red staining indicating myocardial fibrosis, and WGA staining indicating myocardial atrophy are shown. Scale bar: 50 μm. **g** Statistics of vacuolization in ventricular tissues. **h** The fibrotic area per left ventricle was quantified. **i** Cell size was quantified. **j** Plasma BNP levels (marker of heart failure) were measured. **k** Western blot analysis of pyroptosis-related protein levels in mouse ventricular tissue. “E” indicates early diastolic transmitral flow velocity, “E′” indicates early diastolic mitral annular velocity, “A” indicates late (atrial) diastolic transmitral flow velocity, “ns” indicates non-significant, and “DOX” indicates doxorubicin. Data are presented as means ± SD

To obtain more conclusive evidence that miR-216a-5p from BCC-derived EXOs exacerbates adriamycin-induced cardiomyopathy, we used a cardiomyocyte-specific AAV9 miR-216a-5p sponge. To determine the knockdown efficiency of cardiac miR-216a-5p, we first examined its expression levels in heart tissue. Compared with both the saline and AAV9-NC sponges, the AAV9-miR-216a-5p sponges significantly reduced cardiac miR-216a-5p expression (Supplementary Fig. 16a). Under saline treatment, neither the cardiomyocyte-specific AAV9-NC sponges nor the AAV9-miR-216a-5p sponges altered cardiac function parameters (Supplementary Fig. 16b–e), myocardial morphology (Supplementary Fig. 16f–i), plasma BNP levels (Supplementary Fig.16j), or the expression of pyroptosis-related proteins (Supplementary Fig. 16k–n). The mice were injected locally with 4T1 cells four weeks after AAV9 injection and were administered DOX (5 mg/kg) weekly when the tumor reached ~100 mm^3^. (Fig. 9a). The results indicated that the AAV9-miR-216a-5p sponges, but not the AAV9-NC sponges, substantially reversed the DOX-induced decreases in the E/E’ ratio, E/A ratio, LVEF, and LVES, as shown by echocardiography (Fig. 9b–e). Moreover, the AAV9-miR-216a-5p sponges reduced DOX-induced vacuolization of ventricular tissue, as shown by H&E staining (Fig. 9f, g). Sirius red staining also demonstrated that the AAV9-miR-216a-5p sponges decreased DOX-induced cardiac fibrosis (Fig. 9f, h). As shown by WGA staining, the AAV9-miR-216a-5p sponges attenuated DOX-induced myocardial atrophy (Fig. 9f, i). Similarly, the plasma levels of the heart failure marker BNP were dramatically decreased by the AAV9-miR-216a-5p sponges (Fig. 9j). Additionally, the AAV9-miR-216a-5p sponges dramatically downregulated the expression of the pyroptosis-related markers N-terminal GSDMD, cleaved caspase-1, and cleaved IL-1 (Fig. 9k). These data demonstrated that the inhibition of miR-216a-5p could attenuate DOX-induced myocardial injury in vivo.

To determine the optimal therapeutic window for intervention, we performed weekly echocardiographic assessments of DOX-treated mice, which revealed significant cardiac dysfunction beginning at week 2, as evidenced by a decreased LVEF and LVFS, along with altered E/E’ and E/A ratios, in the DOX group compared with the DMSO control group (Supplementary Fig. 17).

To validate the therapeutic effect of the AAV9 miR-216a-5p sponges on the D-BCC-EXO-induced exacerbation of DOXIC, we administered AAV9 at week 2, when cardiac function started to be significantly impaired, as revealed by serial echocardiography (Supplementary Fig. 18a). Compared with mice injected with the NC sponges, mice receiving the AAV9 miR-216a-5p sponges presented improved cardiac function, as evidenced by preserved LVEF and LVFS, an increased E/E’ ratio, and an increased E/A ratio (Supplementary Fig. 18b–e). Histological analysis further revealed that the miR-216a-5p sponges reduced cardiomyocyte vacuolization, cardiac fibrosis, and atrophy (Supplementary Fig. 18f–h) and decreased plasma BNP levels (Supplementary Fig. 18i).

To further validate the role of the miR-216a-5p/ITCH axis in a more clinically relevant patient-derived xenograft (PDX) model, we established a breast cancer PDX model in female NSG mice using primary tumor tissues (Supplementary Fig. 19a) and administered AAV9 at a different time point (for treatment rather than prevention; 2 weeks after DOX exposure, when cardiac function started to be significantly impaired, as revealed by serial echocardiography) (Supplementary Fig. 19b). Echocardiographic examination revealed that compared with DOX alone, the AAV9-miR-216a-5p sponges significantly improved cardiac function, as evidenced by increases in the E/A ratio, E/E’ ratio, LVEF, and LVFS. However, this protective effect was partially abolished by ITCH knockdown (Supplementary Fig. 19c–f). Moreover, plasma BNP levels were significantly lower in the AAV9-miR-216a-5p sponge group than in the DOX group, while ITCH knockdown partially reversed this effect. Consistently, H&E staining revealed that the AAV9-miR-216a-5p sponges markedly attenuated DOX-induced cardiomyocyte vacuolization, whereas shITCH reversed this protective effect (Fig. S18g–i). Sirius red staining revealed reduced cardiac fibrosis in the AAV9-miR-216a-5p sponges group, which was aggravated by ITCH knockdown (Supplementary Fig. 19j, k). WGA immunofluorescence staining further confirmed that the AAV9-miR-216a-5p sponges suppressed DOX-induced cardiomyocyte atrophy, and this effect was compromised by shITCH (Supplementary Fig. 19m, n). These findings further corroborated the therapeutic value of targeting the miR-216a-5p/ITCH axis in DOXIC.

### Pharmacological targeting of Caspase-1/NLRP3/TXNIP signaling prevents DOX-induced cardiotoxicity in a PDX breast cancer model

**Fig. 10:**
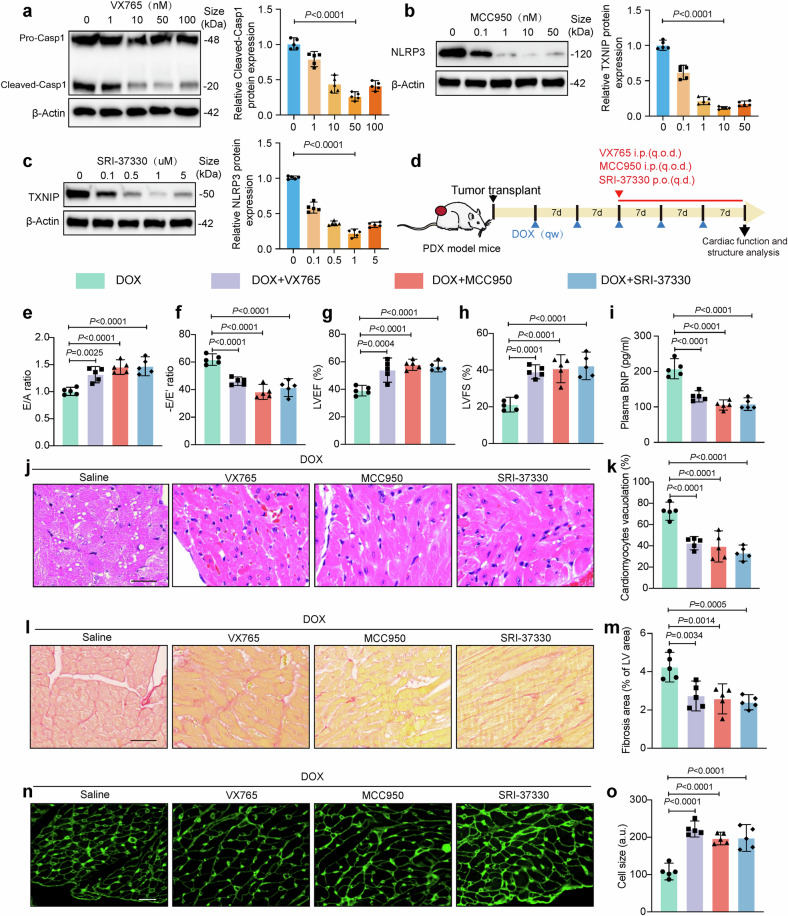
Treatment with TXNIP/NLRP3/Caspase-1 pathway inhibitors attenuates DOX-induced cardiotoxicity in PDX models. **a**–**c** Western blot analysis of target proteins in DOX (1 μM), 24 h)-treated adult murine ventricular cardiomyocytes (AMVCs) co-cultured with 4T1 cells upon VX765, MCC950, or SRI-37330 treatment. **d** Schematic diagram showing the treatment protocol of PDX model mice with DOX and inhibitor. **e**–**i** Cardiac function parameters including E/A ratio, -E/E′ ratio, left ventricular ejection fraction (LVEF), left ventricular fractional shortening (LVFS), and plasma BNP levels. **j** Representative image of H&E staining. Scale bar: 50 μm. **k** Quantification of cardiomyocyte vacuolation. Scale bar: 50 μm. **l** Representative image of Sirius red staining. Scale bar: 50 μm. **m** Quantification of cardiac fibrosis area. **n** Representative image of wheat germ agglutinin (WGA) staining. **o** Quantification of cardiomyocyte size. “i.p.(q.o.d.)” indicates intraperitoneal injection every other day; “p.o.(q.d.)” indicates oral administration once daily, “E” indicates early diastolic transmitral flow velocity, “E′” indicates early diastolic mitral annular velocity, “A” indicates late (atrial) diastolic transmitral flow velocity, and “DOX” indicates doxorubicin. The schematic diagram part was created using SMART - Servier Medical Art by Servier. Data are presented as means ± SD

To increase the translational significance of our study, the effects of three small-molecule inhibitors, VX765, which specifically inhibits Caspase-1,^35^ MCC950, which specifically inhibits the NLRP3 inflammasome,^36^ and SRI-37330, which specifically inhibits TXNIP,^37^ on DOXIC in a PDX breast cancer model were examined. Western blot analysis revealed that VX765, MCC950, and SRI-37330 dose-dependently suppressed the expression of cleaved-Casp1 and TXNIP/NLRP3 in DOX-treated AMVCs cocultured with 4T1 cells (Fig. 10a–c). Echocardiographic examination revealed that all three inhibitors significantly improved cardiac function, as evidenced by increases in the E/A ratio, E/E’ ratio, LVEF, and LVFS in the inhibitor-treated groups compared with the DOX group (Fig. 10e–h). Moreover, plasma BNP levels were significantly lower in the inhibitor groups than in the DOX group (Fig. 10i). Consistently, histological analysis revealed that all three inhibitors significantly attenuated DOX-induced cardiomyocyte vacuolization (H&E staining) (Fig. 10j, k), reduced DOX-induced cardiac fibrosis (Sirius red staining) (Fig. 10l, m), and suppressed DOX-induced cardiomyocyte atrophy (WGA immunofluorescence staining) (Fig. 10n, o). These findings provided preliminary evidence for their potential application in the clinical treatment of DOXIC.

### EXOs from patients receiving chemotherapy aggravate cardiomyocyte pyroptosis, which is reversed by a miR-216a-5p inhibitor

**Fig. 11:**
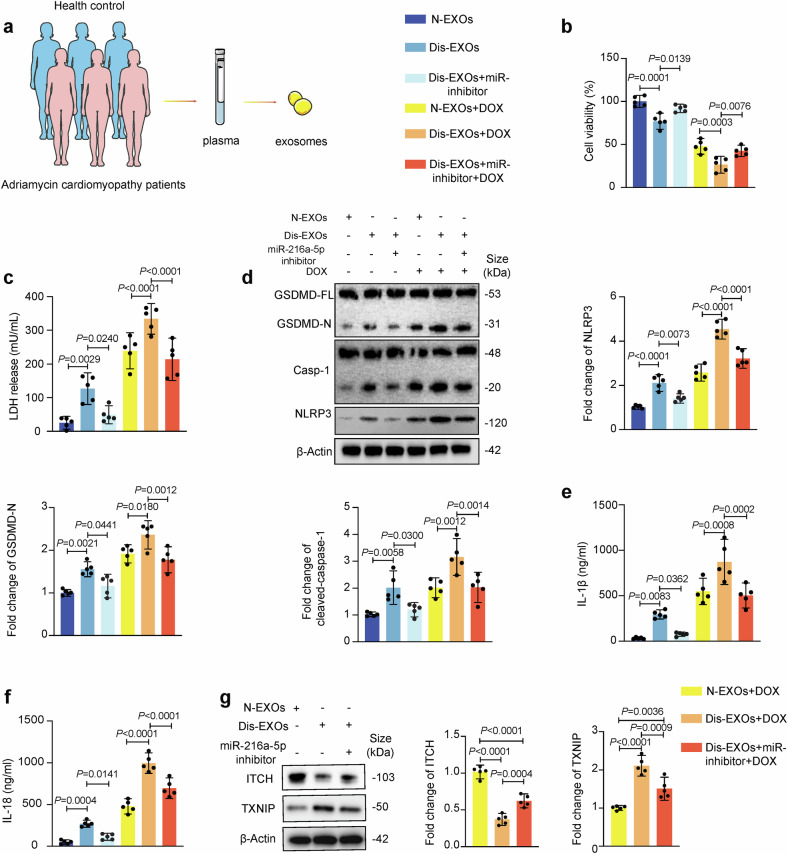
Human exosomes aggravate DOX-induced cardiomyocyte pyroptosis, an effect attenuated by a miR-216a-5p inhibitor. **a** EXOs were isolated from cardiomyopathy patients with adriamycin (Dis-EXOs) and matched healthy donors (N-EXOs). Human induced pluripotent stem cell-derived cardiomyocytes (hiPSC-CMs) were transfected with a miR-216a-5p inhibitor/NC for 24 hours and then treated with Dis-EXOs/N-EXOs or DOX/DMSO for 24 hours. **b** Cell viability was determined (*n* = 5). **c** LDH release (**d**) the levels of pyroptosis-related proteins (GSDMD-N, cleaved-CASP1, and NLRP3) were assessed by western blotting. **e**, **f** The levels of pyroptosis-related cytokines (IL-1β and IL-18) were assessed (*n* = 5). **g** Western blotting was used to assess ITCH and TXNIP levels after cells were treated with Dis-EXOs (*n* = 5). “DOX” indicates doxorubicin. The schematic diagram part was created using SMART - Servier Medical Art by Servier. Data are presented as means ± SD

EXOs were isolated from the plasma of five breast cancer patients who were diagnosed with DOXIC to verify whether the plasma EXOs of these patients had similar effects. EXOs isolated from patients with DOXIC (Dis-EXOs) and from age- and sex-matched healthy donors (N-EXOs) were used (Fig. 11a). The Dis-EXOs and N-EXOs were characterized by TEM, NTA, and Western blotting (Supplementary Fig. 20a–c). The expression level of miR-216a-5p in Dis-EXOs was approximately 2 times greater than that in N-EXOs. Additionally, we found that Dis-EXOs aggravated DOX-induced cardiomyocyte injury, as evidenced by increases in the expression of pyroptosis-related proteins (N-terminal GSDMD, cleaved caspase-1, and NLRP3), LDH release, the production of pyroptosis-related cytokines (IL-1β and IL-18), and a decrease in cell viability (Fig. 11b–f). These effects were reversed by transfection with a miR-216a-5p inhibitor. After treating hiPSC-CMs with Dis-EXOs, we measured ITCH and TXNIP expression levels. We found that ITCH expression decreased whereas TXNIP expression increased after Dis-EXO treatment and that these changes were reversed by transfection with a miR-216a-5p inhibitor (Fig. 11g). These data indicated that Dis-EXOs aggravated DOX-induced cardiomyocyte injury via the miR-216a-5p/ITCH/TXNIP axis.

## Discussion

The present study yielded five novel findings. First, we provide concrete evidence that tumor cells can impact DOX-induced cardiotoxicity through heart–tumor communication *via* EXOs and can be blocked by the inhibition of exosomal secretion by shRab27a in breast cancer tissue. Second, we found that miR-216a-5p is a key molecule and promising therapeutic target for DOXIC in BCC EXOs, as evidenced by the mechanistic investigation and curative effect of blocking myocardial miR-216a-5p. Third, we identified ITCH as a new downstream target mRNA of miR-216a-5p. The ubiquitination and degradation of TXNIP are reduced by miR-216a-5p-mediated ITCH degradation, which subsequently activates TXNIP/NLRP3, thereby exacerbating DOXIC. Fourth, we found that miR-216a-5p transcription is upregulated by the DOX-induced increase in ATF3 in breast cancer cells. Finally, we discovered that SF3B4 preferentially binds to miR-216a-5p and selectively packages it into 4T1 EXOs.

Patients receiving cancer therapy are more likely to suffer from cardiovascular illnesses, such as heart failure, coronary artery disease, and cardiac arrhythmias.^38^ Therefore, CVD has emerged as a significant factor in the morbidity and death of cancer survivors.^39^ The majority of preclinical and clinical research on the classical cardiotoxicity-inspired approach to cardio-oncology is aimed at improving our understanding of the specific effects of anticancer therapy on the heart in cancer patients, as well as how these effects may increase oxidative stress and inflammation in cardiomyocytes.^40,41^ However, the understanding of the mechanisms linking cardiovascular disease to cancer and the methods for monitoring this condition remain insufficient. This highlights the need for a more comprehensive understanding of the relationship between cardiology and cancer to elucidate the processes of cardiotoxicity, thus enabling more effective stringent surveillance and treatment strategies.^42–44^ Increasing evidence points to biochemical crosstalk between the heart and tumors, and these associations add to the complexity of the relationship between cancer and cardiovascular disease.^45^ Recent data have indicated that cardiovascular diseases, such as heart failure, may promote tumor growth.^46^ A previous study revealed that increased amounts of the tumor metabolite D-2-hydroxyglutarate (D2-HG) are produced by IDH2-mutant leukemia cells, leading to cardiac systolic dysfunction.^45^ Direct evidence is still scarce, although many studies have suggested that cancer and cardiac disease share some mechanistic pathways.^47,48^ Our data reveal a novel mechanism by which DOX indirectly injures cardiomyocytes by inducing pathogenic exosome production in breast cancer cells, as demonstrated in a coculture system and in a tumor-bearing mouse model. These data indicate that breast cancer makes a noteworthy contribution to the development of DOXIC, which has implications for the use of cardiac risk stratification in the management of breast cancer patients during chemotherapy and cardiotoxicity control.

Recent evidence indicates that EXOs play crucial roles in modulating cross-organ communication.^49,50^ In EXO-mediated communication between the heart and other organs, miRNAs have attracted much attention. Brown adipose tissue-derived EXOs participate in exercise-induced cardioprotective mechanisms by delivering cardioprotective miRNAs to the heart.^51^ Xia et al.^52^ reported that macrophage-derived EXOs treated with PD-1 inhibitors promote cardiomyocyte senescence by regulating the miR-34a-5p/PNUTS signaling pathway. In addition, hypo-EXO-derived miR125b-5p has been shown to facilitate ischemic cardiac repair by ameliorating cardiomyocyte apoptosis.^53^ Pathologic communication between tumors and the heart can also be mediated by EXOs. Recently, studies have shown that EXOs secreted by the myocardium after MI can promote tumor growth.^22^ During our research, we discovered that DOX therapy considerably altered the pathological biogenesis in tumor cell EXOs. In this study, we observed that DOX-induced significantly more exosome biogenesis and secretion in 4T1 BCCs, which was in accord with a previous study.^54^ In line with this observation, we also observed elevated expression of TSG101 in the lysis of DOX-treated exosomes. Of note, TSG101 plays a significant role in enhancing exosome production through its involvement in the endosomal sorting complex required for transport (ESCRT) pathway, which is critical for exosome biogenesis.^55^ Future studies are warranted to investigate whether and how TSG101 enhances exosome production in DOX-treated BCCs. Moreover, few studies have investigated the treatment targets of adriamycin-induced cardiomyopathy resulting from tumor intervention strategies. This study investigated novel therapeutic targets to treat DOXIC through the knockdown of Rab27a in tumor tissues to block the release of EXOs.

According to our data, the effect of BCC-EXO-induced additional cardiac injury can be reduced by deleting miRNAs in BCCs using the Dicer enzyme knockout system, which can inhibit the miRNA biosynthesis process in BCCs. These findings suggest that miRNAs, which are abundant in cancer cell EXOs, may be involved in the pathological process of DOXIC. We found that EXOs from DOX-induced breast cancer cells aggravated DOX-induced cardiomyocyte toxicity, whereas those from untreated breast cancer cells did not. The expression of miR-216a-5p was elevated in EXOs isolated from DOX-treated mouse plasma, BCCs, and BCT. Mechanistically, DOX upregulates ATF3 in BCCs, thereby promoting the transcriptional activation of miR-216a-5p. Furthermore, we confirmed that miR-216a-5p is selectively packaged into EXOs by SF3B4 in BCCs. These findings suggest that miR-216a-5p may be a key molecule in EXOs that exacerbates adriamycin-induced cardiomyopathy. Previous studies have reported that miR-216a-5p is expressed in a variety of cancer tissues,^56–59^ which suggests that the pathological effects of DOX chemotherapy need to be further investigated in the treatment of other malignancies. To investigate the clinical translational perspective of this study, we isolated plasma EXOs from adriamycin cardiomyopathy patients. The level of miR-216a-5p was found to increase approximately twofold, which induced the same pathological changes in cardiomyocytes treated with DOX in vitro, and this effect was reversed by the miR-216a-5p inhibitor. Additionally, our study revealed that targeting the myocardium using an AAV9 vector packed with a miR-216a-5p sponge can reduce the pathological effect of BCC-EXOs that exacerbate DOX-induced myocardial injury, suggesting a promising therapeutic target for DOXIC.

Through bioinformatics analysis and experimental verification, ITCH was shown to be the downstream molecular target of miR-216a-5p, which acts as an E3 ubiquitin ligase^31,33,60^ and is widely involved in various cardiovascular diseases.^61,62^ The elevated miR-216a-5p in EXOs from adriamycin cardiomyopathy patients induced cardiotoxicity in hiPSC-CMs, consistent with previous reports linking miR-216a-5p to cardiac dysfunction.^63^ To validate the miR-216a-5p/ITCH axis in vivo while enhancing translational relevance, we established PDX models using patient-derived breast cancer tissues. Notably, AAV9-mediated myocardial delivery of miR-216a-5p sponges markedly improved cardiac function and attenuated DOX-induced myocardial injury in the PDX mice model. Mechanistically, the protective effects of miR-216a-5p inhibition were largely abolished by ITCH knockdown, establishing miR-216a-5p/ITCH as a pivotal regulatory axis in DOXIC pathogenesis. These findings suggest that targeting exosomal miR-216a-5p may represent a promising therapeutic strategy for DOXIC. TXNIP is an endogenous negative regulator of thioredoxin (TXN) that is induced by various cellular stresses, including ischemia, oxidative stress, and apoptosis signaling.^64^ The interaction between ITCH and TXNIP has been previously reported. ITCH has been shown to target the ubiquitin‒proteasome-mediated degradation of TXNIP in cardiomyocytes and alleviates reactive oxygen species-induced cardiotoxicity through the thioredoxin system.^32^ In addition, numerous studies have demonstrated that TXNIP can cause pyroptosis by activating the NLRP3/GSDMD pathway.^34,65^ Furthermore, recent studies have shown that DOX activates the NLRP3 inflammasome and caspase-1, causing cardiomyocyte pyroptosis, which plays an important role in the progression of myocardial dysfunction and the pathogenesis of DCM.^66^ Our data revealed that miR-216a-5p induced the downregulation of ITCH expression, resulting in decreased TXNIP ubiquitination, which led in turn to the upregulation of TXNIP expression and the activation of the NLRP3 inflammasome, further aggravating DOXIC. Despite these novel insights into breast cancer-derived miR-216a-5p and its role in DOX-induced cardiac injury, our study has several limitations. First, our findings indicated that breast cancer cells played a significant role in DOXIC, but we also recognized that more research was needed to determine the involvement of EXOs from other cancer types in DOXIC. Second, we were unable to conduct the correlation analysis between miR-216a-5p and ITCH in human subjects due to the insufficient number of autopsy myocardial specimens for ITCH-level detection. Third, due to current technical limitations, high-throughput screening of therapeutic small molecules in the co-culture system remains to be explored, and their translational value needs to be further validated through clinical trials.

The potential therapeutic application of miR-216a-5p inhibition deserves careful consideration of its systemic effects. On one hand, the role of miR-216a-5p in cancer biology is complex, as it exhibits both tumor-suppressive and oncogenic properties depending on the context. In various cancers, including pancreatic cancer, small cell lung cancer, and breast cancer, miR-216a-5p has been identified as a tumor suppressor.^59,67,68^ Conversely, in certain contexts, miR-216a-5p can act as an oncogene such as renal cell carcinoma, gastric cancer, prostate cancer, and esophageal carcinoma.^59,67–69^ On the other hand, miR-216a-5p plays significant roles in various normal tissues and organs beyond its well-documented involvement in tumor biology. miR-216a-5p has been shown to be protective in the context of acute kidney injury (AKI).^70^ miR-216a-5p has also been implicated in protecting vascular endothelial cells from damage induced by lipopolysaccharide.^71^ Therefore, while targeting miR-216a-5p presents a promising avenue for cancer therapy and cancer therapy-induced cardiac dysfunction, its dual roles necessitate a careful evaluation of potential trade-offs that could affect both tumor dynamics and overall systemic health. In this scenario, improving the cardiac specificity of miR-216a-5p inhibition by nanocarriers with cardiac targeting property might be necessary.

To our knowledge, this is the first study to verify that EXO-packaged miR-216a-5p is the key pathogenic factor and therapeutic target for DOXIC. We determined that DOX promoted ATF3 expression, leading to increased miR-216a-5p expression in BCCs. Additionally, SF3B4 was shown to mediate the packaging of miR-216a-5p into EXOs in DOX-treated breast cancer cells. EXO-packaged miR-216a-5p was then taken up by cardiomyocytes and aggravated DOX-induced pyroptosis through the ITCH/TXNIP/NLRP3 axis. Furthermore, we developed several effective strategies that may relieve DOXIC by blocking pathological BCC-CM communication, including inhibiting the release of BCC-EXOs, preventing aberrant BCC-EXO secretion, silencing miR-216a-5p, and interfering with the exosomal packaging of miR-216a-5p. These findings may provide novel insights into cancer–heart cross talk in cardio-oncology.
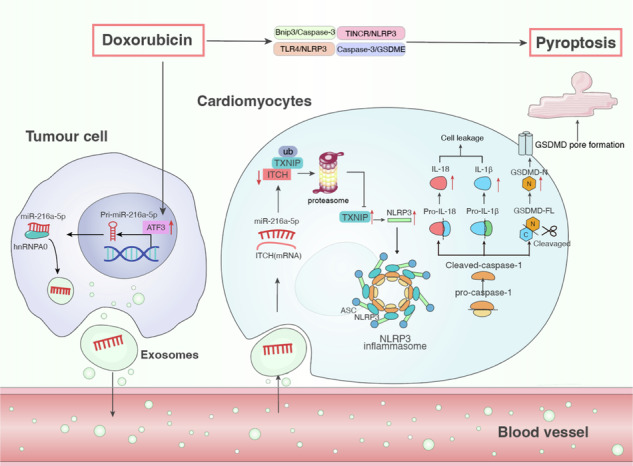


## Materials and methods

### Mouse study approval

All studies were conducted following the National Institutes of Health Guidelines for the Care and Use of Laboratory Animals and were approved by the Ethics Committee of the Chinese People’s Liberation Army General Hospital (2021-X17-105).

### Human clinical samples

The study was approved by the Ethics Committee of the Chinese People’s Liberation Army General Hospital (S2021-400-02). All patients provided written informed consent for participation in this study, and all procedures were conducted following the Declaration of Helsinki. Clinical samples were collected under approved IRB guidelines and coded to ensure subject anonymity.

### Statistical analysis

Statistical analysis was performed using the statistical analysis program GraphPad Prism 8 (Graph Pad Software, Inc.). The Kolmogorov–Smirnov test and the Shapiro–Wilk test was used to determine the normality of the data distribution. For continuous data with a normal distribution, the Brown–Forsythe test was used to check whether the variances were equal. When appropriate, an unpaired Student’s *t* test was used to analyze the differences between the two groups. For multiple sets of data, a single- or two-way analysis of variance (ANOVA) was used, and the Bonferroni multiple comparison test was used when 3 or more groups needed to be compared. The results are presented as the means ± standard deviations. *P* < 0.05 was considered statistically significant.

### Mouse treatment

Eight-week-old female BALB/c mice were randomly divided using a simple and free online randomization tool in GraphPad (http://www.grahpad.com/quickcalcs/randomizel.cfm). Identical volumes of 0.1% dimethyl sulfoxide in phosphate buffer saline (Saline) were administered to control animals. Subchronic DICM (DOX group) models of DOX exposure were generated in mice via the intraperitoneal injection of 5 mg/kg DOX every week for 0, 7, 14, 21, and 28 days (with a cumulative dose of 25 mg/kg body weight). On day 35, the structure and function of the heart were analyzed.

### In situ BALB/c mouse model of breast cancer

To establish a breast cancer mouse model, eight-week-old BALB/c mice were anesthetized using inhaled isoflurane. The parameters used were an oxygen flow rate of 1 L/minute and an isoflurane concentration of 1%. Tumor cells (10^5^ 4T1 cells (BALB/c mouse-derived breast cancer cell line)) suspended in 10 µL of PBS were injected into the fourth pair of breast fat pads after a longitudinal incision was made at the intersection of the fat and the abdomen. After tumor development, the tumor volume was measured using a Vernier caliper. The formula used to calculate the tumor volume was as follows: tumor volume = (D×d2) ×2 (according to a previous report),^72^ where D represents the long diameter of the tumor and d represents the short diameter of the tumor. When the tumor volume reached an appropriate size,^10^ the mice were randomly divided into different groups, and follow-up experiments were conducted. Noninvasive cardiac function tests were performed, and serological myocardial enzyme indexes were measured.

### Knockdown of Rab27a

For 4T1 cell transfection, shNC and a plasmid (pcDNA3.1^+^) expressing shRNA targeting Rab27a were purchased from Genepharma Biotechnology Company (Shanghai, China). A total of 2 µg of plasmid was transfected into 4T1 cells seeded in a 6-well plate at a density of 80% with Lipofectamine^TM^ 3000 according to the manufacturer’s instructions. After transfection, stable cell lines were screened with neomycin.

For intertumoral injection, breast cancer model mice were randomly divided into the shNC and shRab27a groups to study the effects of BCC-derived EXOs on DOX-induced myocardial injury. Once the tumor diameter reached approximately 0.5 to 0.6 cm,^10^ intratumoral injection was performed with an insulin syringe with a 31 G needle under ultrasound guidance. For ultrasound-guided intratumoral injection, the mice were sedated via inhaled isoflurane (oxygen flow rate of 1 L/min, isoflurane concentration of 1%), placed in the supine position, and fixed to an electrode patch. The ultrasound probe was adjusted to provide a parasternal short axis view to reveal the tumor, avoiding the blood vessels. The animals in the experimental group then received an intratumoral injection of 200 µL of MMP-9-shRNA lentivirus (concentration of 2 × 10^8^ TU/ml; Genechem Co., Shanghai, China), and those in the control group were treated with 200 μL of GFP lentivirus once a week for 3 weeks. The sequence information is listed in Supplementary Table 9.

### Measurement of mouse cardiac function

Cardiac function was measured using noninvasive transthoracic echocardiography, as previously described.^73^ Briefly, the mice were anesthetized using inhaled isoflurane. The parameters used were an oxygen flow rate of 1 L/minute and an isoflurane concentration of 1%. A Visual Sonics Vevo 3100 machine was used to assess the cardiac function of the mice. The mice were placed in the supine position, and a 30 MHz high-frequency transducer was used for analysis. Long-axis and short-axis projections of 2D images and M-mode recordings at the positive ventricular level were captured. The Vevo computer algorithm was used to calculate the LVEF and LVFS. The late (atrial) diastolic transmitral flow velocity (A), early diastolic transmitral flow velocity (E), and early diastolic mitral annular velocity (e’) were measured and recorded using color Doppler images of the mitral valve via color Doppler flow imaging. The Vevo computer algorithm was used to calculate the E/A ratio and the E/E’ ratio.

### Hematoxylin‒eosin (H&E) staining, Sirius red staining, and wheat germ agglutinin (WGA) staining

H&E staining, Sirius red staining, and WGA staining were performed as described previously.^7^ Briefly, samples were fixed in 4% paraformaldehyde, embedded in paraffin, and cut into 3.0–5.0 μm thick cross-sections. An H&E staining kit (C0105s, Beyotime, China) was used for H&E staining following the manufacturer’s instructions. Collagen fibers were stained with a Sirius red staining kit (36324, Yeasen, China). Myocardial atrophy was detected via an Alexa Fluor 488-labeled WGA staining kit (W11261 Thermo Fisher Scientific, USA). For each heart section, five images of different regions were captured, and the areas of nearly 100 cells were measured and averaged (depending on cell availability). The degree of fibrosis was determined by the percentage of the Sirius red-stained area in each heart section.

### Establishment of PDX models

Female NSG (NOD.Cg-PrkdcscidIl2rgtm1Wjl/SzJl) mice (6–8 weeks old) were purchased from Beijing SPF Biotechnology Co., Ltd. and maintained under pathogen-free conditions. Fresh breast cancer samples were collected from patients undergoing surgery at the Department of General Surgery at Chinese PLA General Hospital. The protocol was approved by the Ethics Committee of Chinese PLA General Hospital (Ethics approval number: S2021-400-02), and signed informed consent was obtained. All animal procedures were performed following protocols approved by the Institutional Animal Care and Use Committee. The PDX donor was a 44-year-old female diagnosed with T2N1M0 stage invasive breast carcinoma exhibiting poor differentiation (Grade III), triple-negative (ER-, PR-, HER2-) breast cancer (TNBC), and a Ki-67 index of 57–63%.

Fresh breast tumor samples were collected immediately after surgical resection and maintained in ice-cold DMEM/F12 supplemented with 1% penicillin/streptomycin (P/S). After removing necrotic and normal adjacent tissues, viable tumor tissue was mechanically dissociated and cut into uniform 3 mm³ fragments. For the initial engraftment (P0), tumors were implanted subcutaneously in the dorsolateral flank region, approximately 1–2 cm above the hind limb of anesthetized NSG mice. When the P0 tumors reached approximately 1000 mm³, they were harvested for passaging. The harvested tumors were cut into smaller fragments and reimplanted into new immunocompromised mice using the same surgical technique used for initial implantation to establish P1 generation PDX models, which were used for all subsequent experiments in this study. Once the tumors reached ~100 mm³ in diameter, DOX treatment was started.

### AAV9 delivery in mice

To investigate the cardioprotective effects of miR-216a-5p inhibition and ITCH knockdown in PDX models, the mice were administered DOX (5 mg/kg) weekly beginning when the tumor diameter reached ~100 mm^3^. At week 3, the mice were intravenously injected with AAV9-miR-216a-5p sponges and AAV9-shITCH. At week 5, cardiac function was evaluated via echocardiography, followed by histological analysis. For the inhibition of miR-216a-5p, AAV9-miR-216a-5p sponges and negative control sponges (AAV9-NC sponges) were purchased from Genechem (Shanghai, China). The constructs consisted of an AAV vector containing cTnT promoter elements specific to the chicken heart (cTnTp-MCS-SV40 PolyA). On the basis of the miRNA sequences and their “seed” sequences, bulged/imperfect complementary design methods were used to create the miR-216a-5p sponges.^74^ The EGFP coding sequence was inserted before the sponge sequence and after the cTnT promoter. Each mouse was intravenously injected (via the tail vein) with either 100 μl of AAV9-miR-216a-5p sponges (1012 VG/ml) or scrambled AAV9-NC sponges. For ITCH knockdown, AAV9-shITCH and its corresponding control (AAV9-shNC) were purchased from Genechem (Shanghai), China. The constructs consisted of the GV478 vector (U6-MCS-CAG-EGFP) containing shRNA under the control of the U6 promoter. Three shRNA sequences targeting mouse ITCH (Gene ID: 16396) were designed and validated, with the most effective sequence selected for subsequent experiments. Each mouse was intravenously injected (via the tail vein) with 100 μl of AAV9-miR-216a-5p sponges (1012 VG/ml), scrambled AAV9-NC sponges, AAV9-shITCH (1012 VG/ml), or AAV9-shNC.

### Evaluation of the effects of TXNIP/NLRP3/Caspase-1 inhibitors in AMVCs and PDX models

AMVCs were isolated and cocultured with 4T1 breast cancer cells in a Transwell system. The cells were treated with DOX (1 μM) for 24 h, followed by treatment with varying concentrations of VX765 (0–100 nM), MCC950 (0–50 nM), or SRI-37330 (0–5 μM).

To evaluate the therapeutic effects of small-molecule inhibitors, including VX765, MCC950 and SRI-37330, a PDX breast cancer model was constructed, and DOX treatment was started when the tumors reached ~100 mm³ in diameter. Two weeks after the first two doses of DOX, VX765 (catalog number: S2228; Selleck) was injected intraperitoneally (100 mg/kg) every 2 days for three weeks; MCC950 (catalog number: S8930; Selleck) was injected intraperitoneally (50 mg/kg) every 2 days for three weeks; or SRI-37330 (catalog number: E1272; Selleck) was administered via oral gavage at a dose of 100 mg/kg/day for three weeks. After the treatment period, cardiac function was examined via echocardiography, and the mice were sacrificed for further histological analysis.

### Isolation of cardiomyocytes from adult mice

Left ventricular myocytes from eight-week-old adult mice were isolated using a Langendorff perfusion system and collagenase digestion. The mice were anesthetized via isoflurane inhalation. After the intraperitoneal injection of cefoperazone (2 mg/kg) and heparin (10 U/g), a median sternotomy was performed to expose the heart, which was rapidly resected and washed with saline. The aortic lumen was fixed on an 18 G cannula with Tyrode bicarbonate buffer (PB180341, Procell, China) at 37 °C for 5 minutes. The tissue was then switched to digestion buffer (0.14 mg/mL collagenase type II) in Tyrode bicarbonate buffer (C2-22-1G; Sigma‒Aldrich, Inc., USA) for 12 to 15 minutes. The heart was separated into small pieces with blunt forceps in digestion buffer. The tissue was repeatedly pipetted, filtered through 140 µm nylon mesh, and transferred into Tyrode bicarbonate buffer containing 12.5 M CaCl_2_. The calcium concentration was adjusted to 500 μM by stepwise recalcification at room temperature. The suspension was subsequently placed in a 50 mL conical tube and allowed to settle for 10 minutes at room temperature. The supernatant was transferred to another tube, and the pellet was placed in modified Eagle medium (D8437, Sigma‒Aldrich, Inc., USA) containing 0.1% P/S. The cells were transferred into dishes coated with 5 µg/mL laminin mouse protein (23017015; Thermo Fisher Scientific, USA) and incubated overnight at 37 °C with 5% CO_2_. The medium was changed before the experiment to wash away unattached cells for subsequent studies.

### Cell culture and coculture system

hiPSC-CMs were differentiated and cultured as previously described.^75,76^ Beating cardiomyocytes were cultured in cardiomyocyte maintenance medium containing Gibco™ RPMI 1640 medium (11875093, Thermo Fisher Scientific, USA), B27 (10889038, Thermo Fisher Scientific, USA), and 0.1% P/S (PB180120, Pricella Inc., China) for subsequent experiments.

AMVCs were cultured in complete DMEM/F12 (PM150312B; Pricella, Inc., China) containing 10% fetal bovine plasma (A5256801; Thermo Fisher Scientific, USA) and 0.1% P/S. The cells were grown in an incubator at 37 °C with 5% CO_2_. For the cell coculture experiment, BCCs were cultured in the upper layer of a coculture system (140660, Thermo Fisher Scientific, USA), and cardiomyocytes were cultured in the lower layer.

For single culture, 4T1 cells (CL0007, Pricella, Inc., China) were cultured in Gibco™ RPMI 1640 medium (11875093, Thermo Fisher Scientific, USA) supplemented with 10% fetal bovine plasma and 0.1% P/S. MDA-MB-231 cells (C6550, Beyotime, China) were cultured in complete DMEM/F12 (PM150312B, Pricella, Inc., China) containing 10% fetal bovine plasma and 0.1% P/S.

HL1 cells (CL0605, Pricella, Inc., China) were cultured in MEM (PM150410, Pricella, Inc., China) supplemented with 10% fetal bovine plasma and 1% P/S. AC16 cells (SCC109, Sigma‒Aldrich, Inc., USA) were cultured in complete DMEM/F12 supplemented with 10% fetal bovine plasma and 0.1% P/S.

### Treatment of cocultured cells

DOX (Sigma Aldrich, USA) was dissolved in DMSO at a concentration of 10 mM and then added to culture medium to assess the ability of 4T1 cells or MBA-MD-231 cells to affect DOX-induced damage to AMVCs and hiPSC-CMs. The final DOX concentration was 1 μmol/L, and the cells were treated for 24 hours, as described in our previous study.^7^ In the coculture study, cardiomyocytes and BCCs were divided into the following groups and cultured for 24 hours: DMSO group (2.5 × 10^6^ cardiomyocytes treated with an equal volume of solvent), DOX group (2.5 × 10^6^ cardiomyocytes treated with 1 µM DOX), BC group (2.5 × 10^6^ cardiomyocytes cocultured with an equal number of BCCs and treated with an equal volume of DMSO was added), and BC + DOX group (cardiomyocytes cocultured with BCCs and treated with 1 µM DOX).

### Adenovirus transduction

For the knockdown of ITCH and TXNIP, adenovirus vectors expressing shITCH and shTXNIP were purchased from Genechem Co., Shanghai, China (vector No.: hU6-MCS-CMV-EGFP, GV119). For ITCH and TXNIP knockdown, a short hairpin RNA (shRNA) containing a green fluorescent protein (EGFP) transcription box was cloned and inserted into the GV119 vector. The expression of shRNA was driven by the hU6 promoter. The AdEasy and Admax adenovirus packaging systems were used to transfect HEK-293 cells. The virus particles were centrifuged and filtered through an ultrafast density gradient, and the titer was determined by a shell protein immunoassay.

To overexpress ITCH and TXNIP, oeITCH and oeTXNIP adenovirus vectors were synthesized by Genechem Co., Shanghai, China (vector No.: CMV-MCS-3FLAG-SV40-EGFP, GV314). An adenovirus construct containing a mutated 3’-UTR of ITCH (MT1-3’UTR) was produced by RiboBio, Guangzhou, China. The cDNA fragment was inserted into an AdTrack shuttle vector containing a green fluorescent protein (EGFP) transcription box driven by the cytomegalovirus (CMV) promoter, and infection was directly monitored by observing the expression of EGFP.

To explore the mechanism in vitro, cardiomyocytes were infected with adenoviruses as described previously.^77^ Briefly, cardiomyocytes were transduced with an adenovirus at a concentration of 1-5 × 10^10^ pfu/ml for 24–48 hours, after which the medium was replaced with a complete culture medium. The sequence information is listed in Supplementary Table 9.

### Transfection

miR-216a-5p mimics and inhibitors were synthesized by Genechem Co. (Shanghai, China). A total of 10^6^ cardiomyocytes were seeded in a 6-well plate, and the miR-216a-5p mimic, miR-216a-5p inhibitor, or negative control miRNA (NC) were transfected into cells using Lipofectamine 3000 reagent (L3000015, Thermo Fisher Scientific, USA) for the 24 hours according to the manufacturer’s protocol. siACO1, siSF3B4, and their negative controls (siNC) were purchased from MDLBiotech, Beijing, China. BC cells were transfected with siACO1, siSF3B4, siATF3, or siNC using Lipofectamine 3000 according to the manufacturer’s instructions. All sequence data are listed in Supplementary Table 9.

### EXO isolation and characterization

To obtain mouse and human plasma EXOs, a Total EXO Isolation Kit (plasma) (4484450 Thermo Fisher Scientific, USA) was used. Briefly, whole blood was collected and centrifuged twice at 1000 × g for 10 minutes within two hours. At room temperature, the plasma samples were centrifuged at 2000 × g for 20 minutes to remove cells and debris. The supernatant containing part of the clarified plasma was transferred to a new tube. The new tubes were centrifuged at 10,000 × g for 20 minutes to remove debris. The desired volume of clarified plasma was transferred into a new tube, a 0.2 volume of total EXO separation reagent (for isolation from plasma) was added, and the plasma/reagent mixture was evenly mixed by vortexing or pipetting up and down until a uniform solution was obtained. After incubation, the samples were centrifuged at room temperature at 10000 × g for 10 min. The supernatant was aspirated and discarded. The particles were resuspended in 1X PBS. EXOs were stored at ≤20 °C for long periods.

To isolate EXOs from BCC culture medium, the cell culture medium was collected, and a Total EXO Isolation Kit (cell culture) (4478359 Thermo Fisher Scientific, USA) was used to isolate EXOs from the culture supernatants. Briefly, the cell mixture was centrifuged at 2000 × g for 30 minutes to remove cells and debris. The supernatant of the cell-free medium was then transferred to a new tube without disturbing the particles. A total of 0.5 volumes of Total Exosome Isolation reagents (for isolating EXOs from cell culture medium) were added to the medium and mixed evenly by swirling or pipetting up and down until a uniform solution was obtained. The samples were incubated at 2 °C to 8 °C overnight. After incubation, the samples were centrifuged at 10000 × g at 2–8 °C for 1 hour, after which the supernatant was aspirated and discarded. Next, 1X PBS buffer was used to resuspend the particles in preparation for downstream analysis.

The resulting EXOs suspended in PBS were pipetted, mixed, diluted multiple times, and then placed onto copper mesh for electron microscopy. The samples were allowed to stand at room temperature for 4 min, and filter paper was used to absorb the excess liquid. After negative staining with phosphotungstic acid at room temperature for 2 min, filter paper was used to remove the excess liquid. The samples were dried at room temperature, and images were captured via TEM. A NanoSight LM10 instrument (Malvern, UK) was used for particle size distribution and concentration analysis. The concentration of EXOs was quantified according to the total protein concentration using a BCA protein concentration assay kit (P0012S; Beyotime, China). The levels of exosomal-specific proteins, including HSP70 (ab2787, Abcam), CD81 (ab109201 Abcam, USA), TSG101 (ab125011, Abcam, USA), calnexin (ab22595, Abcam, USA), and GM130 (ab52649, Abcam, USA), were assessed via Western blot analysis.

#### Preparation of conditioned medium

Conditioned medium (CM) was prepared as follows: MDA-MB-231 cells were seeded in 6-well plates at a density of 2.5 × 10^6^ cells/well and cultured in DMEM supplemented with 10% FBS for 24 hours to allow cell attachment. The cells were then washed three times with PBS and cultured for an additional 48 hours in an exosome-depleted serum medium (prepared using serum ultracentrifuged at 120,000×g for 18 h). The cell supernatant was collected and centrifuged at 300×g for 10 minutes to remove cell debris, followed by centrifugation at 2,000×g for 20 minutes to eliminate large cell debris and apoptotic bodies. The supernatant was filtered through a 0.22 μm filter and divided into two portions: one portion underwent concentration for conditioned medium preparation, while the other portion underwent further processing for exosome isolation. The conditioned medium portion was concentrated using Amicon Ultra-15 centrifugal filter units (10 kDa molecular weight cut-off, Millipore) to achieve a 30× concentration (equivalent to exosomes isolated from equal cell numbers via commercial kit and resuspended in PBS). The concentrated conditioned medium (CM) was then aliquoted and stored at -80 °C until use.

### Western blotting

Gels were prepared using a TGX Stain-Free FastCast Acrylamide Kit (10%, BioRed Laboratory, USA). Proteins that migrated to the separation gel were transferred to a PVDF membrane (BioRed Laboratories, USA) via the wet transfer method (300 mA for 120 minutes). After transfer, the PVDF membrane was incubated with 5% skim milk on a shaker for 1 hour at room temperature. The PVDF membrane was incubated with primary antibody overnight at 4 °C. The PVDF membrane was immersed in a secondary antibody mixture, incubated at room temperature for 1 hour, and washed with TBST buffer. ECL luminescent solution was used to detect the signals with a Bio-Rad gel imaging system. Images were collected, and the gray value of each band was calculated using ImageJ software (FiJi for Mac OS X). The levels of pyroptosis-related proteins, including TXNIP (ab188865, Abcam, USA), ITCH (ab108515, Abcam, USA), NLRP3 (ab263899, Abcam, USA), GSDMD (ab239377, Abcam, USA), Caspase-1 (#89332, Cell Signaling Technology, USA), and IL-1 beta (ab283818, Abcam, USA), were assessed via Western blot analysis.

### CRISPR/Cas9-mediated gene knockout

Dicer knockout BCCs were generated using paired CRISPR/Cas9, as described by Wettstein et al.^78^ The Dicer CRISPR/Cas9 KO Plasmid (sc-431380, Santa Cruz Biotech, Germany) and UltraCruz® Transfection Reagent (sc-395739, Santa Cruz Biotech, Germany) were used to knock out the dicer gene in 4T1 cells according to the manufacturer’s protocol. A total of 2.5 × 10^5^ cells were seeded into a 6-well culture plate containing 3 mL of standard growth medium without antibiotics 24 hours before transfection. Plasmid DNA solution (Solution A) was added directly to diluted UltraCruz® transfection reagent (Solution B) and incubated at room temperature for 20 minutes. A total of 300 µl of plasmid DNA/UltraCruz® transfection reagent mixture (solution + solution B) was added to each well. GFP fluorescence was observed 48 hours later via fluorescence microscopy. The cell culture medium was replaced with a fresh medium containing the appropriate concentration of puromycin. The medium was then replaced with freshly prepared selective medium every 2 days. RNA was isolated via guanidine thiocyanate‒phenol chloroform extraction and analyzed via qRT‒PCR. The sequences are listed in Supplementary Table 9.

### Real-time quantitative PCR

For quantification of miRNA levels, total RNA was extracted from tissues, cells, and EXOs using an RNAiso Small RNA Kit (9735 A; TAKARA, Japan) following the manufacturer’s instructions. Total RNA was extracted from 200 µg EXOs. RNAiso was added to the total RNA, followed by homogenization. To extract total exosomal RNA, EXOs were resuspended in PBS, after which RNAiso was added. One microgram of total RNA was used to synthesize cDNA using the Mir-X^TM^ miRNA First-Stand Synthesis Kit (688313, TAKARA, Japan). A SYBR Premix Ex Taq^TM^ II Kit (A46109, TAKARA, Japan) was used for qRT‒PCR. Relative gene expression data were analyzed using cycle threshold values. Considering that U6 RNA may be unstable in EXOs,^79^ cel-miR-39 was used as the control for miRNA expression. All the data are expressed as the fold increase over the control group. The sequences of the primers used are listed in Supplementary Table 9.

For quantification of mRNA levels, total RNA was extracted from cells using TRIzol reagent (15596026, Thermo Fisher Scientific, USA) and reverse transcribed into cDNA using the PrimeScript^TM^ RT Reagent Kit with gDNA Eraser (RR047A TAKARA, Japan). Real-time quantitative PCR (RT‒qPCR) was performed using TB Green Premix Ex TaqTM II (RR820A, TAKARA, Japan) and a Step OnePlus Real-Time PCR System (Applied Biosystems). GAPDH was used as the control for mRNA expression. The primer sequences are presented in Supplementary Table 9.

### Phagocytosis of EXOs

4T1 and MDA-MB-231 cells were seeded (1 × 10^6^ cells/well) onto chamber slides and treated with 1 µM DOX or DMSO for 48 hours. A Total EXO Isolation Kit (4478359 Thermo Fisher Scientific, USA) was used to isolate EXOs from the culture supernatants. The extracted EXOs were resuspended in 150 μL of PBS and frozen at 80 °C for subsequent experiments. EXOs were labeled using a PKH67 Green Fluorescent Cell Linker Kit (PKH67PCL, Sigma‒Aldrich, Inc., USA). In the PBS control group (without EXOs), a volume of PBS equivalent to the volume of extracted EXOs used in the other groups was added to the medium as the vehicle control. After the cardiomyocytes were incubated for 12 hours, a confocal microscope was used to detect the PKH67 fluorescence signal. Sodium pentobarbital (3%, 60 mg/kg) was used to anesthetize the mice to study the capacity of EXOs to be taken up by cardiomyocytes in vivo. For EXO treatment experiments, 10 µg of purified EXOs in 100 µL of PBS (EXO group) or an equivalent volume of PBS (PBS group) was administered to the mice by tail vein injection one time per week, with treatments beginning one week after DOX administration. Tail vein injection was selected as it resembles the route of delivery for circulating tumor-derived exosomes to the heart and is comparatively less invasive and more convenient for translation to clinics. The PKH67 signals were detected via confocal microscopy 24 hours after AMVCs were isolated, as described above.

### Sequencing of exosomal miRNAs and data analysis

Blood samples were collected by enucleation under isoflurane anesthesia and immediately transferred to EDTA-coated tubes, followed by centrifugation at 3,000 g for 15 min at 4 °C to obtain plasma. Hearts were excised after euthanasia, and rinsed with ice-cold PBS, and left ventricles were dissected and snap-frozen in liquid nitrogen. EXOs were isolated from the plasma of orthotopic breast cancer model mice. The peripheral blood of ten mice was extracted from the orbital vein. Approximately 5 mL of whole blood was collected into tubes and allowed to coagulate for 30 minutes at room temperature. The sample was subsequently centrifuged at 2500 rpm for 10 minutes to obtain plasma (upper layer). EXOs were extracted using a Total EXO Isolation Kit (plasma) (4484450 Thermo Fisher Scientific, USA). Total RNA from EXOs was extracted using a Total Exosomal RNA and Protein Isolation Kit (4478545; Thermo Fisher Scientific, USA) following the manufacturer’s protocol. Small RNAs in total RNA were detected by Nuohe Biological Co. Ltd. The construction and sequencing of the small RNA library were completed by Nuohe Biological Co. Ltd. Then, the cDNA library was sequenced using an Illumina HiSeq 2500. The raw data were collected using Illumina analysis software. For the data analysis, the criterion for significantly differential expression was set at a *q* value < 0.05 and |log2(fold change) | > 1. Using R packages, volcano plots, and hierarchical clustering heatmaps were generated to visualize the differentially expressed miRNAs.

### Study of human plasma EXOs

Participants were recruited on the basis of direct recommendations by breast surgeons and oncologists at the People’s Liberation Army General Hospital. The included patients were female, aged 40 to 75 years, diagnosed with BC, and scheduled for DOX treatment. This study was approved by the Ethics Committee of the People’s Liberation Army General Hospital. Eight breast cancer patients and healthy donors were recruited from the Department of Medical Oncology between June 1, 2021, and May 31, 2022 (Supplementary Table 8). All study subjects were of Han nationality. An informed consent form was signed by all the subjects before inclusion in the study.

The diagnostic criteria for DOXIC at the follow-up examination after DOX therapy were as follows (https://www.uptodate.cn/contents/clinical-manifestations-diagnosis-and-treatment-of-anthracycline-induced-cardiotoxicity): (1) new significant left ventricular systolic dysfunction (decline in LVEF of >10 percentage points but ≤40 percentage points or an absolute decline in LVEF of ≤15 percentage points or >40 percentage points and/or HF) or (2) an asymptomatic decline in LVEF of <50 percentage points but >40 percentage points.

Participants were excluded if they met the following conditions: (1) had a history of structural heart disease or congenital heart disease; (2) had severe blood disorders, active liver disease, other malignant tumors, inflammation in the past two months, or infectious diseases; or (3) used other chemotherapy drugs.

Five patients diagnosed with DOXIC were selected. Whole blood samples were collected in tubes without anticoagulants and allowed to coagulate for 30 minutes at room temperature. The samples were subsequently centrifuged at 2000 rpm for 15 minutes, after which the plasma was stored at −80 °C for subsequent experiments. EXOs were extracted from the cell supernatants using a Total EXO Isolation Kit (plasma) (4484450 Thermo Fisher Scientific, USA).

AMVCs were treated with a miR-216a-5p inhibitor or the corresponding negative control (NC) for 24 hours to investigate the effect of human EXOs on DOXIC following the manufacturer’s protocol. A total of 1 × 10^6^ AMVCs were treated with 20 µg of EXOs and 1 µM DOX or an equal volume of medium for 24 hours after transfection with the miRNA inhibitor.

### Correlation analysis of miR-216a-5p levels and cardiotoxicity in breast cancer patients

An additional 36 patients (mean age 48 ± 8 years) who were evaluated at our institute between 2020 and 2022, met the aforementioned criteria, and experienced DOXIC were retrospectively recruited to perform a correlation analysis of miR-216a-5p levels and cardiotoxicity. All of them underwent 6 cycles of anthracycline-based chemotherapy for histopathologically confirmed breast cancer (after surgery). The total planned DOX dose was ≥300 mg/m^2^. The breast cancer treatment regimen was DOX 40–60 mg/m² and cyclophosphamide 400–600 mg/m² per cycle. Individuals were excluded if they had baseline myocardial injury (cardiac troponin I [cTnI] concentration of ≥0.04 ng/mL), an LVEF < 50%, or other serious organ diseases. Ethical approval was obtained from the Human Ethics Committee of Chinese PLA General Hospital, and all participants provided written informed consent.

Plasma miR-216a-5p levels in frozen blood that had been collected in conventional EDTA tubes were measured by qPCR. TnI concentrations were analyzed at a centralized laboratory at the time of collection at our institution. Plasma cTnI concentrations were measured within 24 hours after the completion of chemotherapy infusion. The cTnI level was measured using a fluorometric enzyme immunoassay analyzer (Tosoh Bioscience, Inc.) with low-end sensitivities of 0.06 ng/mL (MSKCC) and 0.04 ng/mL (DF/HCC). Furthermore, cardiac assessment was performed via echocardiography. The LVEF (measured via the biplane method according to Simpson’s rule) was evaluated prior to the initiation of DOX chemotherapy and re-evaluated at 2 weeks, 1 month, 3 months, and 6 months after completion of DOX therapy. The maximal reduction in LVEF was calculated and defined as ΔLVEF%. Pearson correlation analysis of plasma miR-216a-5p and cTnI levels, as well as of plasma miR-216a-5p levels and ΔLVEF%, was performed.

### Isolation of EXOs from mouse BCT

For the isolation of EXOs from BCT, BALB/c mouse models of breast cancer were used. BCT was weighed after being cut into pieces (1-2 mm^3^). The tissue was subsequently incubated for 30 minutes at 37 °C in Gibco^TM^ RPMI 1640 medium (11875093, Thermo Fisher Scientific, USA) containing 100 U/mL collagenase D (11088858001, Sigma‒Aldrich, Inc., USA) and 20 U/mL DNase I (90083, Thermo Fisher Scientific, USA). After filtering (0.45 µm; SLHP033RB, Millipore, USA), the supernatant was collected and subjected to gradient centrifugation at 300 × g for 10 minutes, 2,000 × g for 10 minutes, and 10,000 × g for 30 minutes to remove cells and debris. After being filtered through a 0.45 µm porous membrane, the supernatant was centrifuged at 120,000 × g for 70 minutes using a Ti70 rotor-equipped L-80XP ultracentrifuge (Beckman Coulter, Brea, California, USA). The pellet was redissolved in PBS, and the suspension was kept at −80 °C until needed for experiments.^80^

### Biotin miRNA pull-down assay

Biotin-labeled WT and MT miR-216a-5p constructs were synthesized by Genechem Co. (Shanghai, China) and transfected into 4T1 cells at 20 °C for 48 hours. 4T1 cell lysates and EXO extracts from cell lysates were obtained using Total Exosome Isolation Reagent (4478359; Thermo Fisher Scientific, USA) in accordance with the manufacturer’s instructions. The EXOs and 4T1 cell lysates were subsequently incubated overnight at 4 °C, followed by incubation with Pierce™ Streptavidin magnetic beads (88816, Thermo Fisher Scientific, USA). Briefly, 50 µL of Pierce Streptavidin magnetic beads were added to a 1.5 mL microcentrifuge tube. Next, the samples were mixed with 10 µg of biotinylated antibodies and incubated at 4 °C overnight. The antibody mixture was added to a 1.5 mL microcentrifuge tube containing prewashed magnetic beads, mixed and incubated at room temperature for 1 hour, after which the supernatant was removed and preserved. Next, 100 µL of elution buffer was added to the test tube. Magnetic separation beads and 100 µL of SDS‒PAGE reduction sample buffer was added to the supernatant containing the target antigen and heated in a heating block at 96‒100 °C for 5 minutes. Finally, the precipitates were washed and analyzed via Western blotting; biotinylated poly (G) (5′-GGGGGGGGGGGGGGGGGGGGG-3′) served as the negative control. The primer sequences are listed in Supplementary Table 9.

### Chromatin immunoprecipitation (ChIP) assay

ChIP assays were performed with an EZ-ChIP Assay Kit (17-371, Millipore, USA) following the manufacturer’s instructions. Briefly, 4T1 cells (10^7^ per assay) were collected, cross-linked with 1% formaldehyde, quenched in glycine, lysed by sonication in the presence of protease inhibitors, and immunoprecipitated with anti-ATF3 antibodies (ab254268, Abcam, USA) or nonspecific anti-IgG antibodies (negative control) (ab171870, Abcam, USA). After washing, the DNA was released, eluted, and amplified via PCR, and the fragments were analyzed via agarose gel electrophoresis. The amount of precipitated DNA was calculated as the percentage of the input. The primers used for ChIP‒PCR were specific for the promoter regions containing putative ATF3 binding sites within miR-216a-5p.

### LDH release assay

Cellular LDH release was measured using an LDH cytotoxicity assay kit (C0016, Beyotime, China). The samples were incubated at room temperature for 30 minutes in the dark. The absorbance was then measured at 490 nm.

### Cell viability assay

Cell viability was assessed with a Cell Counting Kit-8 (CCK-8) assay kit (C0037, Beyotime, China). After treatment, CCK-8 working solution was added to the cells, followed by incubation in an incubator for 0.5 hours. A microplate reader was used to measure the absorbance at 450 nm.

### Dual-luciferase assay

HL-1 and AC16 cells were seeded in 24-well plates and transfected with plasmids expressing WT or MT 3’UTR luciferase reporters (500 ng per well). Forty-eight hours after transfection, luciferase activity was measured using a GloMax-Multi Detection System (E1960, Lumiprobe, US) and a Promega Dual-Luciferase System (E1910, Promega Corporation, USA) according to the manufacturer’s instructions.

### Cell death staining

AMVCs and hiPSC-CMs were separated and placed in eight-well cell culture slides (CCS-8, MatTek Corporation, Slovak Republic). SYTOX (R37109 Thermo Fisher Scientific, USA), α-an anti-actinin antibody (ab68194), and IgG H&L (Alexa Fluor® 555) (A21428, Thermo Fisher Scientific, USA) were used to detect dead cells. The nuclei were stained with DAPI (Beyotime, China), and dead cells were labeled with SYTOX, which passes through the damaged cell membrane. A Nikon AX HD25 confocal microscope was used to capture images. The total number of SYTOX-positive cells in five randomly selected fields was counted.

### ELISA

The secretion of the cytokines IL-18 and IL-1β by cells into the culture supernatant was measured via ELISA. IL-18 and IL-1β ELISA kits (KMC0181, BMS6002-2 Thermo Fisher Scientific, USA) were used following the manufacturer’s instructions. Cardiac troponin I (cTnI) levels were also measured using a cTnI ELISA kit (ab200016, Abcam, USA) according to the manufacturer’s protocol. We tested 5 samples per group, and the values were normalized to those obtained for standards provided by the manufacturer; the significance of differences between groups was analyzed.

### Electrophoretic mobility shift assays (EMSAs)

miRNA-specific probes were labeled with biotin using Roche Biotin RNA Labeling Mix (11685597910, Scientific Laboratory Supplies, UK) according to the manufacturer’s instructions. The binding reaction was performed using a LightShift™ Chemiluminescent EMSA Kit (20148, Thermo Fisher Scientific, USA). A 50-fold molar excess of each probe was added simultaneously with unlabeled oligonucleotides as competitor-labeled probes. To identify RNA-binding proteins, the cell extracts were incubated with anti-SF3B4 antibodies (#68862; Cell Signaling Technology, USA) or nonspecific anti-IgG antibodies (negative control) (ab171870) for 20 minutes before a labeled probe was added. The probe sequences are listed in Supplementary Table 9.

## Supplementary information

Revised Supplementary data_0403 Clean

Revised Supplementary data_0403 Mraked up

Orginal blots

## Data Availability

To enable replication of the findings or procedures following the guidelines provided by the corresponding authors, other researchers will have access to the data, analytical techniques, and research materials. Mouse plasmic exosome miRNA sequencing data have been deposited in the Gene Expression Omnibus (GEO) under accession code GSE192764. Mouse blood and cardiac tissue miRNA sequencing data have been deposited in the Sequence Read Archive (SRA) under accession code PRJNA1218477.
