# Supplementary material for: Exosomal transfer of pro-pyroptotic miR-216a-5p exacerbates anthracycline cardiotoxicity through breast cancer-heart pathological crosstalk
Source: Signal Transduct Target Ther. 2025 May 14;10:157. doi: 10.1038/s41392-025-02245-4 (PMC12075849; doi:10.1038/s41392-025-02245-4)
Supplement: Supplementary file 1 — Revised Supplementary data_0403 Clean [file 41392_2025_2245_MOESM1_ESM.docx]

Supplementary Materials for

**Exosomal transfer of pro-pyroptotic miR-216a-5p exacerbates anthracycline cardiotoxicity through breast cancer-heart pathological crosstalk**

Yan Ma^1, #^, Yongjun Wang^2, #^, Renzheng Chen^1, #^, Yabin Wang^1^, Yan Fang^1^, Cheng Qin^1^, Tianhu Wang^1^, Xiaoying Shen^1^, Tingwen Zhou^2^, Lei Tian^1^, Ting Sun^1^, Li Fan^1^, Xiaoning Wang^3, *^, Dong Han^1, *^ and Feng Cao^1, *^

Correspondence to: fengcao8828@163.com

**This supplementary file includes:**

Figures. S1 to S20

Tables S1 to S9

Captions for Figures S1 to S20


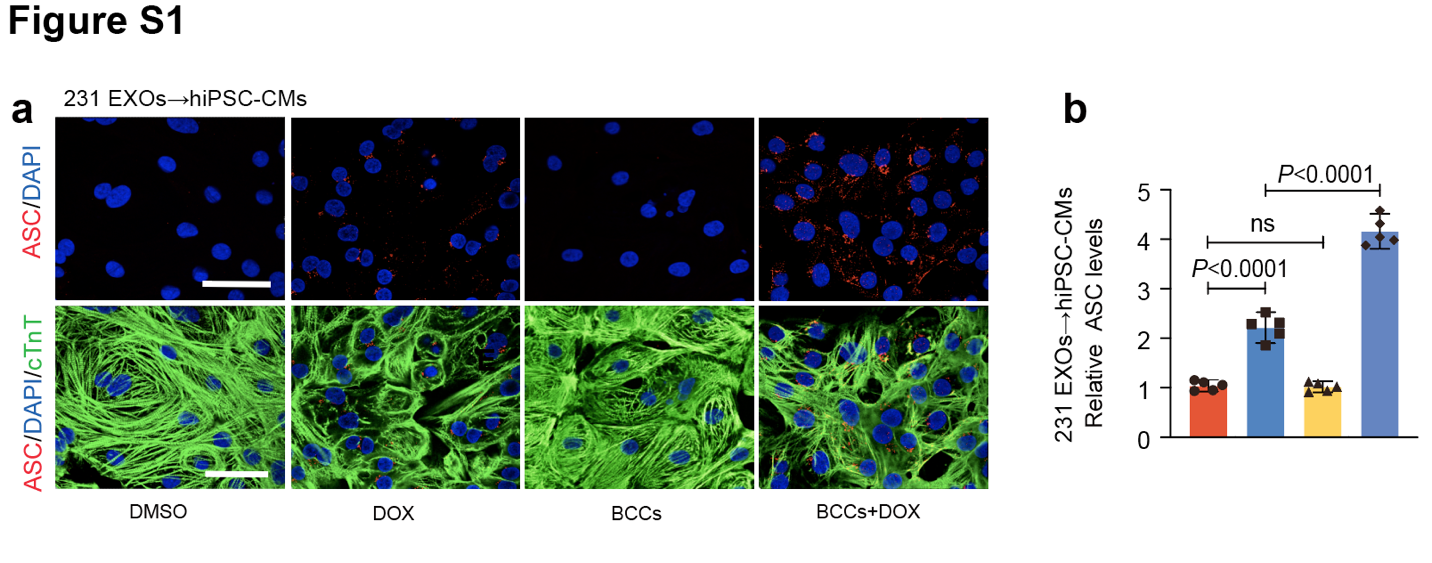


Figure. S1.

**Coincubation with breast cancer cells increases DOX-induced ASC levels in human-induced pluripotent stem cell-derived cardiomyocytes (hiPSC-CMs). (a-b)** ASC levels in the vehicle, DOX, BCC, and BCC+DOX groups were detected by anti-ASC antibody-based immunofluorescence. Scale bar: 50 μm. Red denotes ASC, green denotes α-actinin, and blue denotes DAPI (n = 5). “BCCs” indicates breast cancer cells, “ns” indicates nonsignificant, and “DOX” indicates doxorubicin. Data are presented as means ± SD.


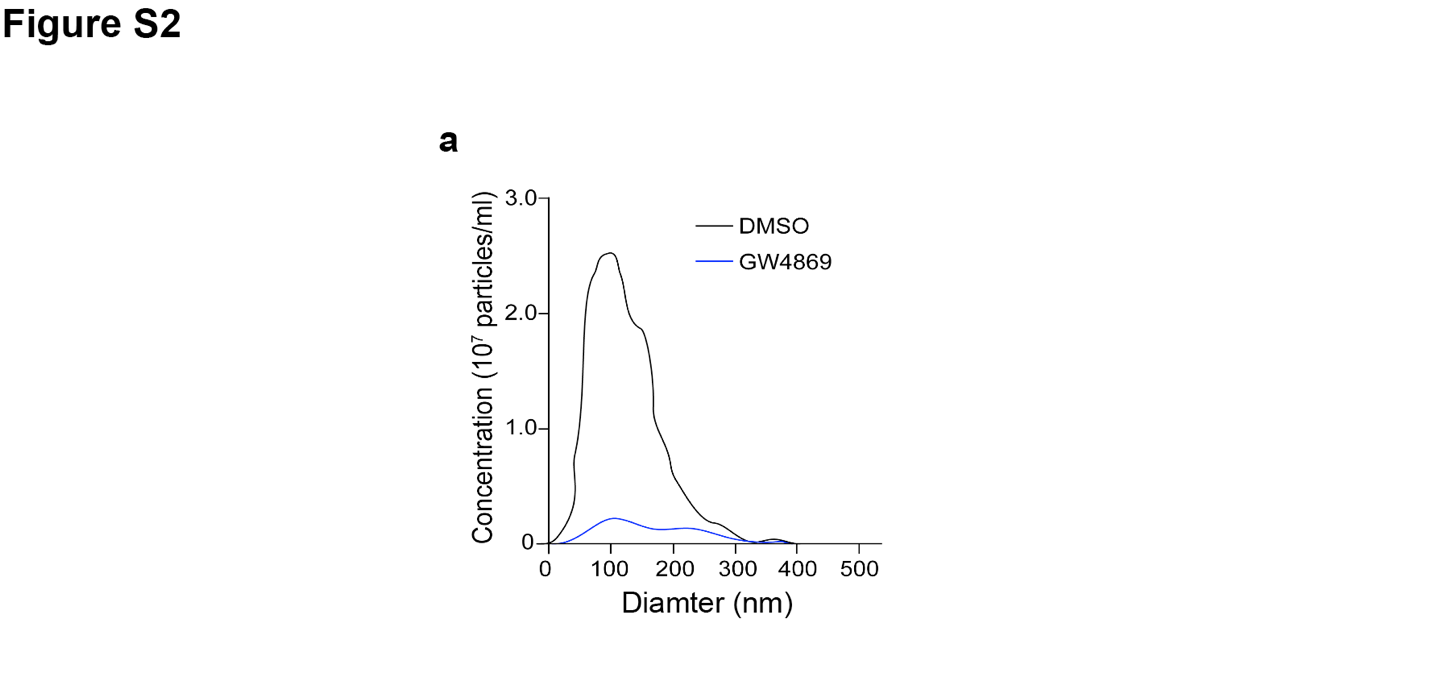


Figure. S2.

**GW4869 inhibits exosome secretion from breast cancer cells. (a)** NTA analysis shows the size distribution and concentration of exosomes isolated from 4T1 breast cancer cells treated with DMSO or GW4869 (10 μM, 24 hours).


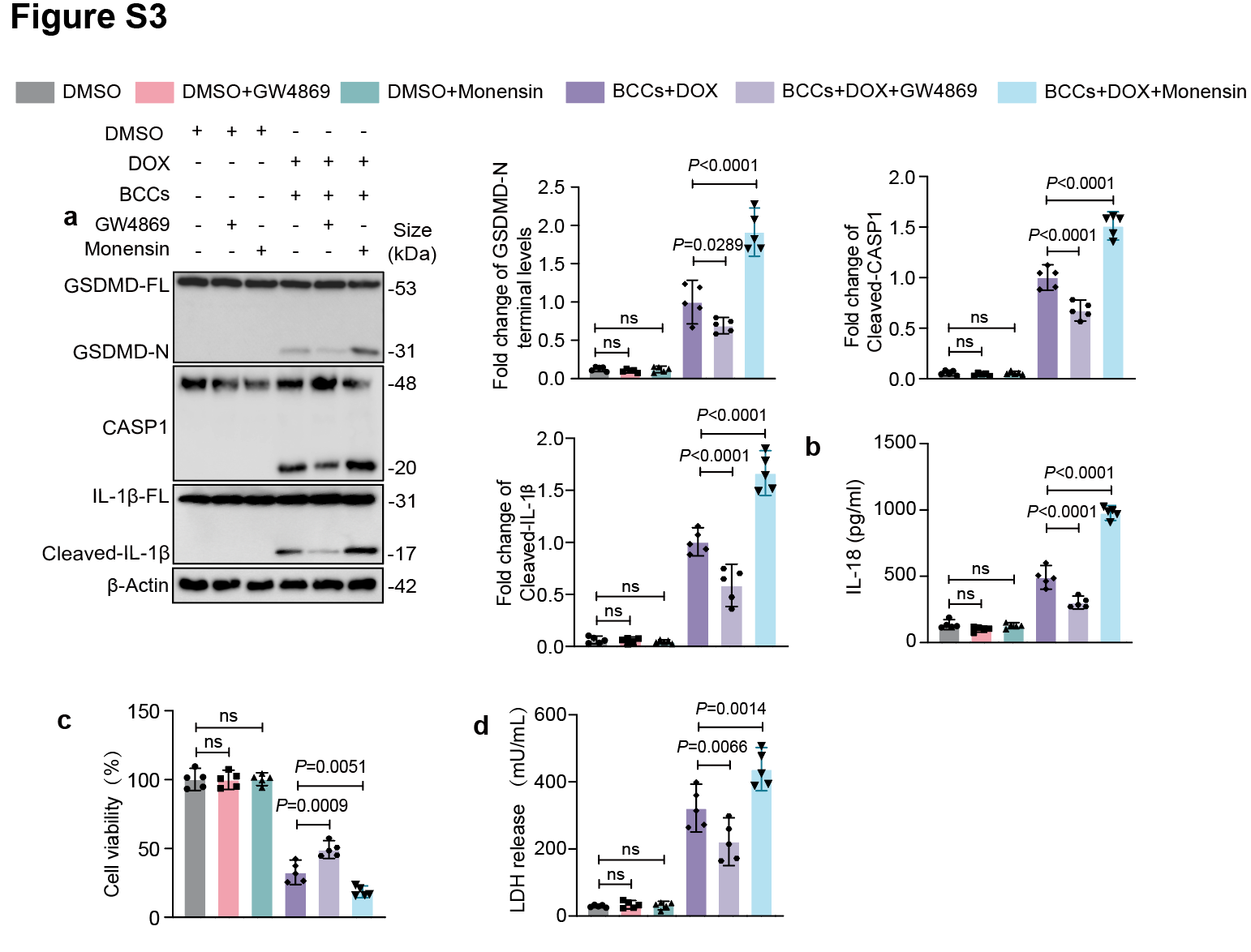


Figure. S3.

**In the cocultured system, the exosome (EXO) release inhibitor GW4869 reduces adult murine ventricular cardiomyocytes (AMVCs) pyroptosis but is aggravated by Monensin.** **(a)** Cell pyroptosis, represented by the levels of GSDMD-N, cleaved-CASP1, and cleaved-IL-1β in AMVCs (n = 5), was detected via Western blotting (using one-way ANOVA, all experimental groups were compared with the DOX+BCCs group). **(b)** The IL-18 level in AMVCs was assessed using a colorimetric method (n = 5). **(c-d)** Cell viability and lactate dehydrogenase (LDH) release levels (n = 5) were measured. “ns” indicates nonsignificant, and “DOX” indicates doxorubicin. Data are presented as means ± SD.


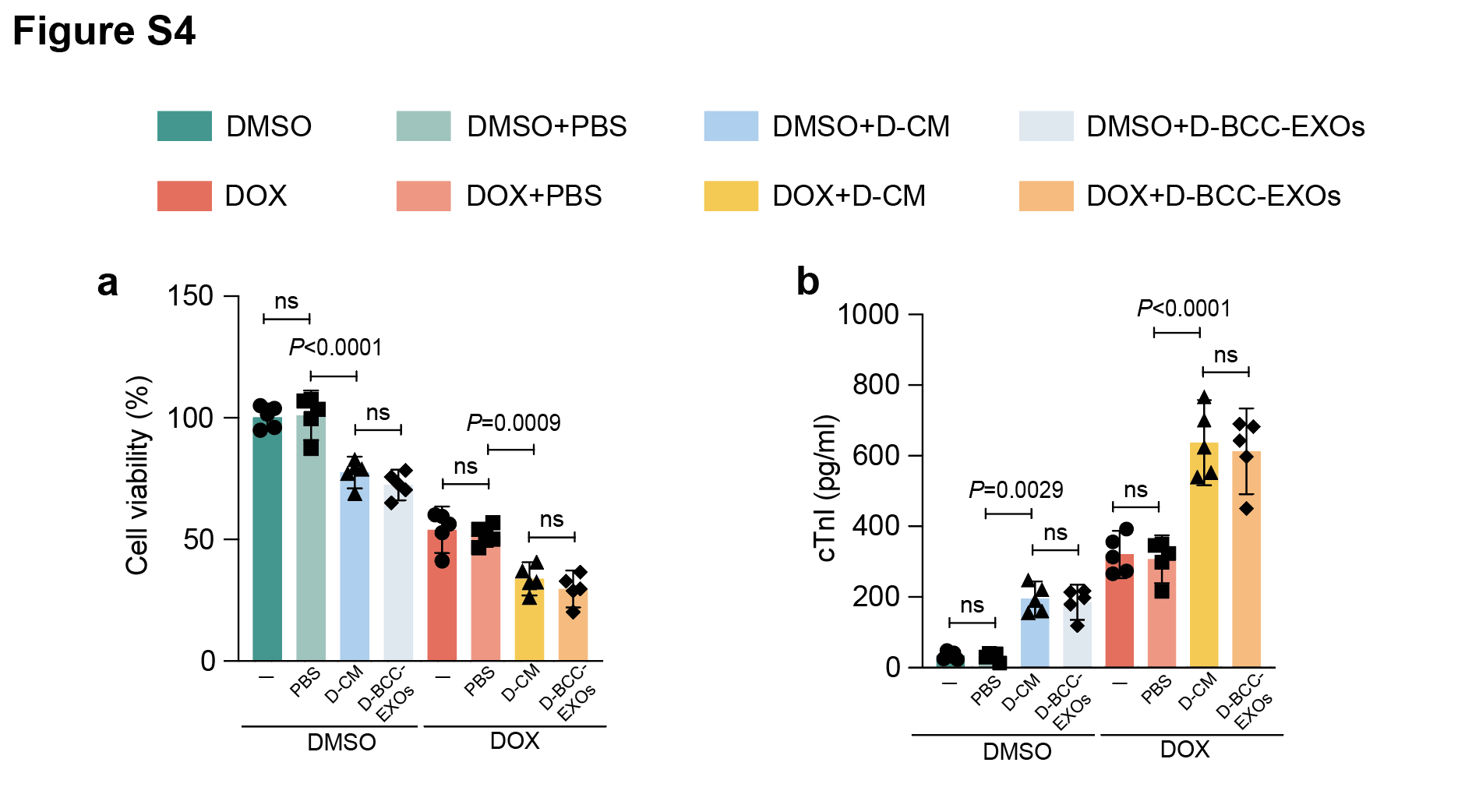


Figure. S4.

**Doxorubicin-damaged breast cancer cell-derived conditioned medium (D-CM) has similar injury effects as D-BCC-EXOs.** D-CM (doxorubicin-induced MDA-MB-231 cell-conditioned medium) and D-BCC-EXOs (DOX-induced breast cancer cell EXOs) were added to DOX-treated cardiomyocytes. (a) Human induced pluripotent stem cell-derived cardiomyocytes (hiPSC-CMs) cell viability was determined using CCK-8 assays (n = 5). (b) cTnI level were assessed using a colorimetric method (n = 5). "ns" indicates non-significant. Data are presented as mean ± SD.


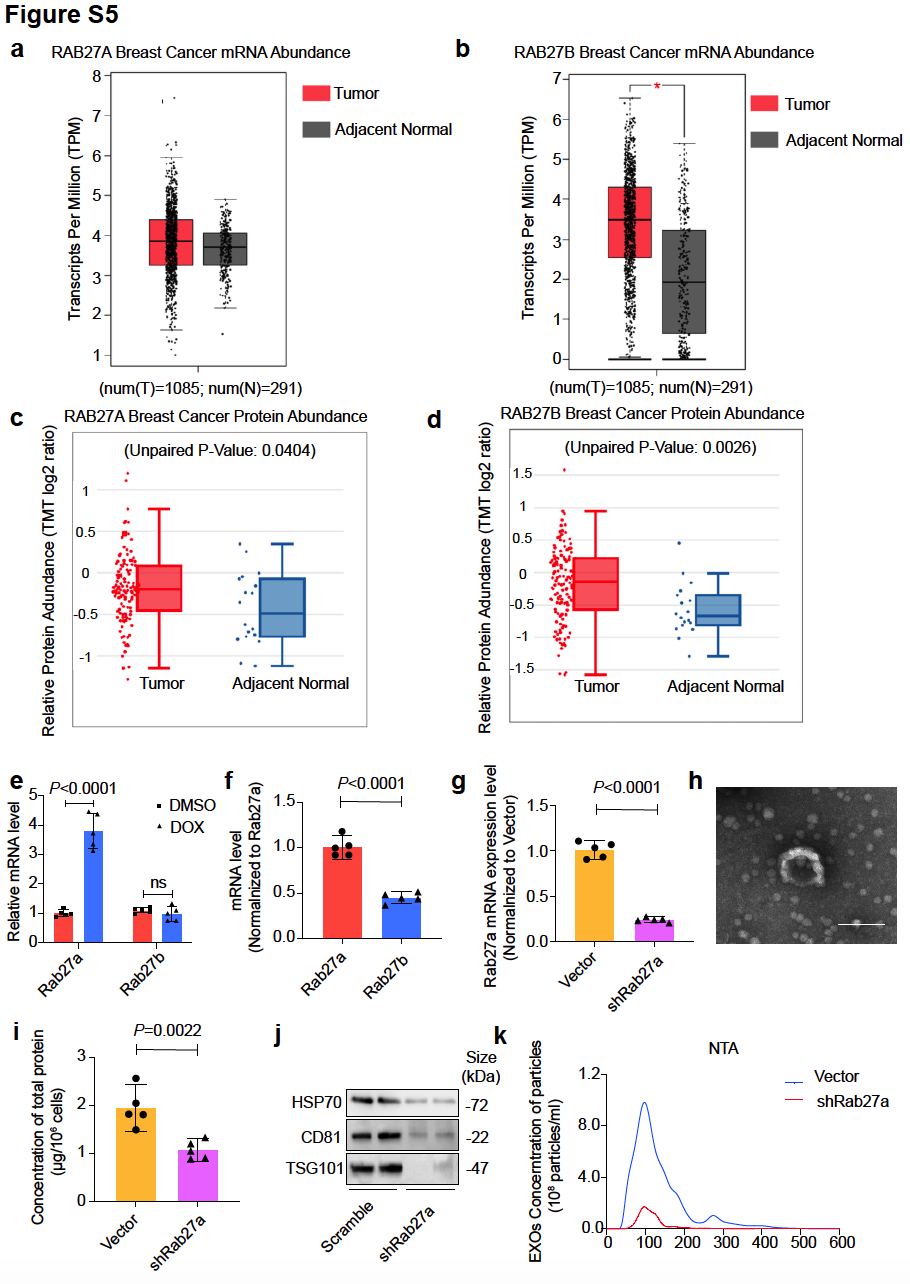


Figure. S5.

The knockdown of Rab27a inhibits the release of EXOs in breast cancer cells. (a-b) Analysis of GEPIA database (RNA sequencing data from TCGA and GTEx projects) showing mRNA levels of Rab27a and Rab27b in breast cancer tumor tissues (red) compared to adjacent normal tissues (grey). (c-d) Analysis of NCI's Proteomic Data Commons showing protein abundance of Rab27a and Rab27b. (e) qPCR analysis of Rab27a and Rab27b mRNA expression in DOX-treated murine breast cancer tissues. (f) Rab27a and Rab27b mRNA levels in the breast cancer tissue (BCT) were measured by qRT-PCR (n = 5). (g) Rab27a mRNA levels in the breast cancer cells (BCCs) were measured by qRT-PCR. (h) Transmission electron microscopy (TEM) was used to examine the morphology of EXOs that were extracted from 4T1 cell culture media. Scale bar: 100 nm. (i) Measurement of protein concentrations in the EXOs extracted from the culture medium of 4T1 cells. (j) EXO markers HSP70, CD81, and TSG101 were analyzed in EXOs isolated from the culture medium of 4T1 cells transfected with either control or shRab27a plasmid. (k) NanoSight tracking analysis of EXOs obtained from the culture media of 4T1 cells transfected with control or shRab27a plasmid. Data are presented as mean ± SD.


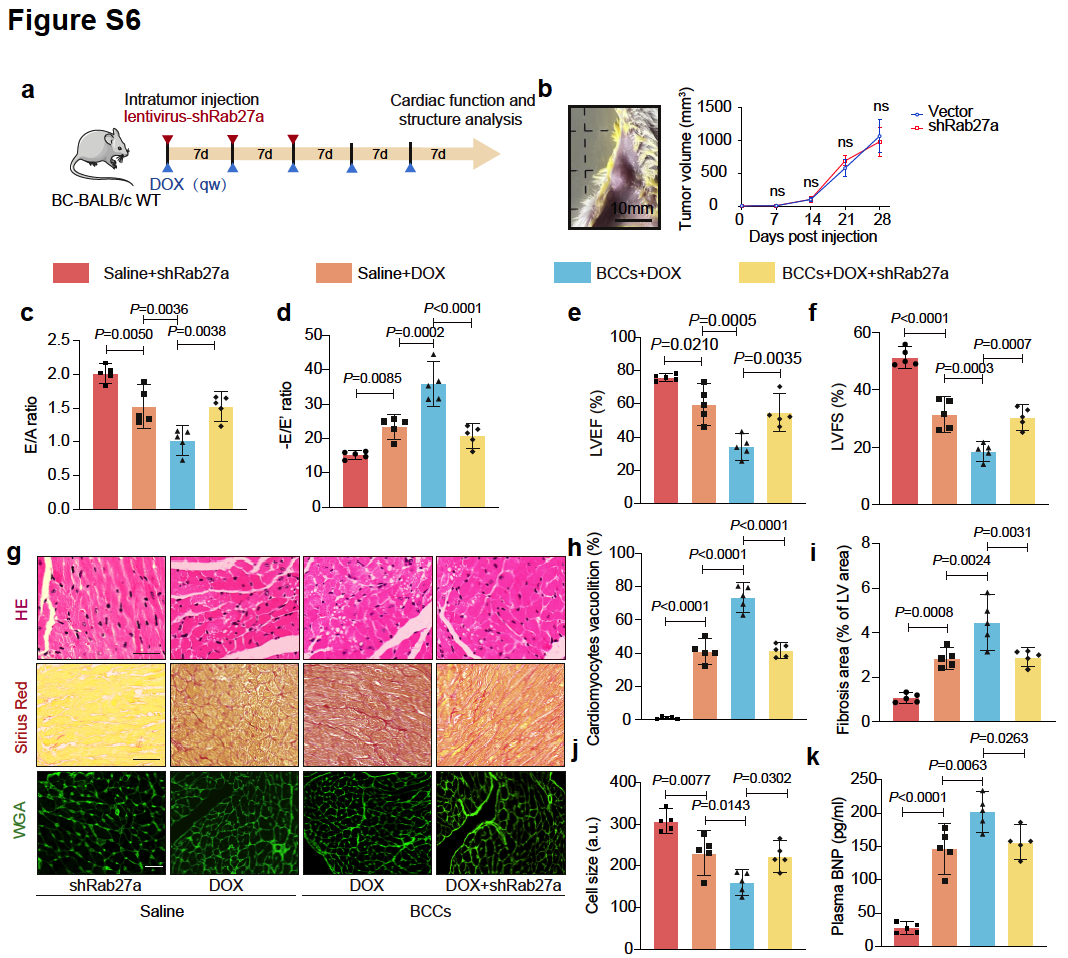


Figure. S6.

**The inhibition of exosomal secretion by shRab27a reversed the breast cancer tissue (BCT) participation in DOX-induced cardiac injury.** **(a)** Schematic illustration showing subchronic DOX injury in breast cancer BALB/c mouse models intratumorally injected with lentivirus-shRab27a (n = 5). **(b)** Image and the tumor volume of the mouse model of breast cancer. **(c-d)** Quantification of the E/A ratio and the -E/E’ ratio via a Doppler echocardiography. **(e-f)** Quantification of the left ventricular ejection fraction (LVEF) and left ventricular fractional shortening (LVFS) via M-mode echocardiography. **(g)** Representative image of hematoxylin-eosin (H&E) staining (upper), Sirius red staining indicating myocardial fibrosis (middle), and wheat germ agglutinin (WGA) staining indicating myocardial atrophy (lower). Scale bar: 50 μm. **(h)** Quantification of vacuolization in ventricular tissues. **(i)** The fibrotic area per left ventricle was quantified. **(j)** Cell size was quantified. **(k)** Plasma levels of brain natriuretic peptide (BNP) were measured. “BCCs” indicate breast cancer cells. “E” indicates early diastolic transmitral flow velocity, “E′” indicates early diastolic mitral annular velocity, “A” indicates late (atrial) diastolic transmitral flow velocity, and “DOX” indicates doxorubicin. Data are presented as means ± SD.


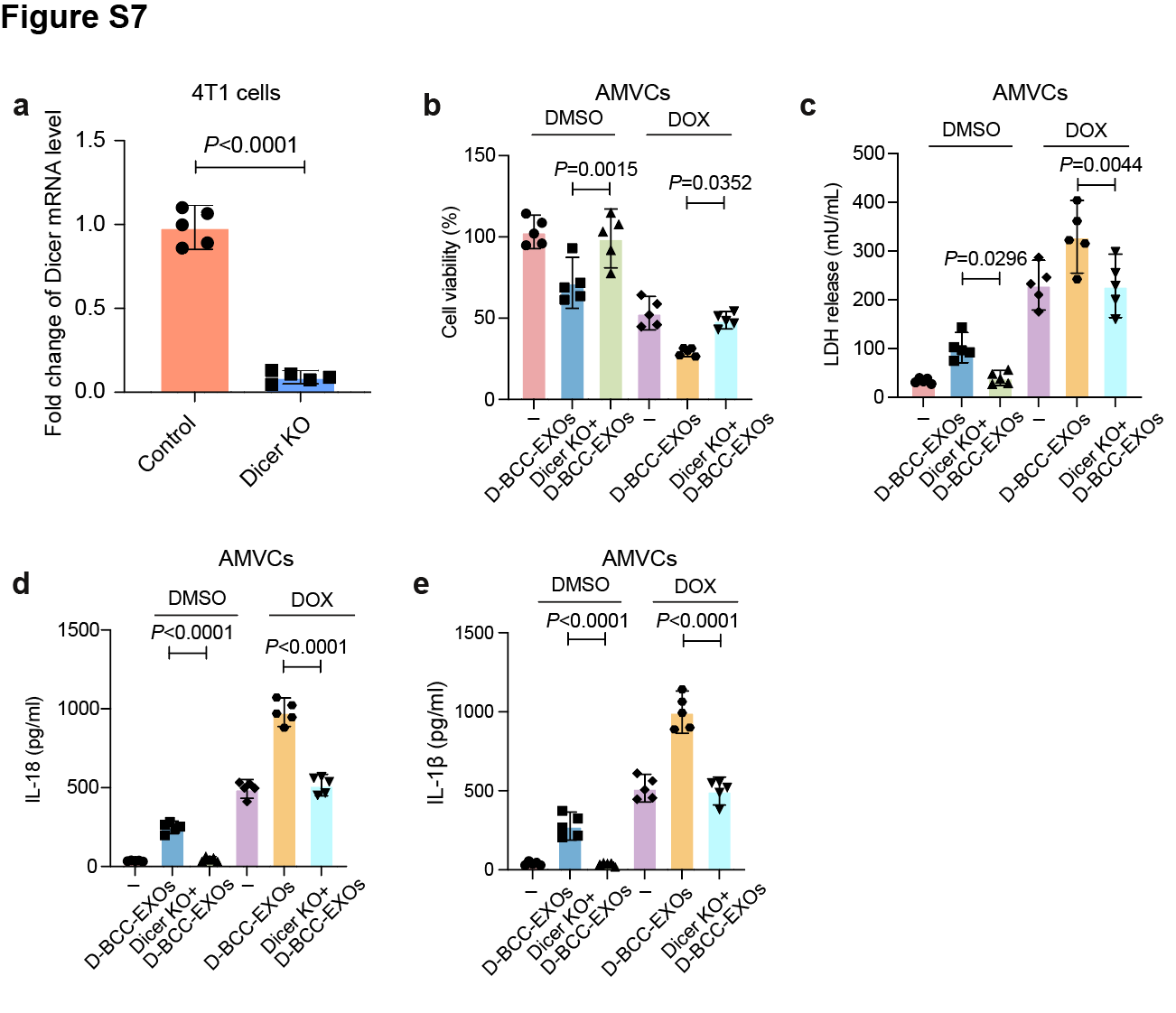


Figure. S7.

**Knocking out Dicer enzymes to delete miRNAs in 4T1 cells can reverse the injury effect of D-BCC-EXOs in DOX-induced cardiomyocytes. (a)** The Dicer mRNA level was detected in 4T1 cells (n = 5). **(b)** Cell viability was determined via CCK-8 assays in AMVCs (n = 5). **(c-e)** LDH release and IL-18 and IL-1β levels in AMVCs were assessed using a colorimetric method (n = 5). “DOX” indicates doxorubicin, “N-BCC-EXOs” indicates normal breast cancer cell EXOs, and “D-BCC-EXOs” indicates DOX-induced breast cancer cell EXOs. Data are presented as means ± SD.


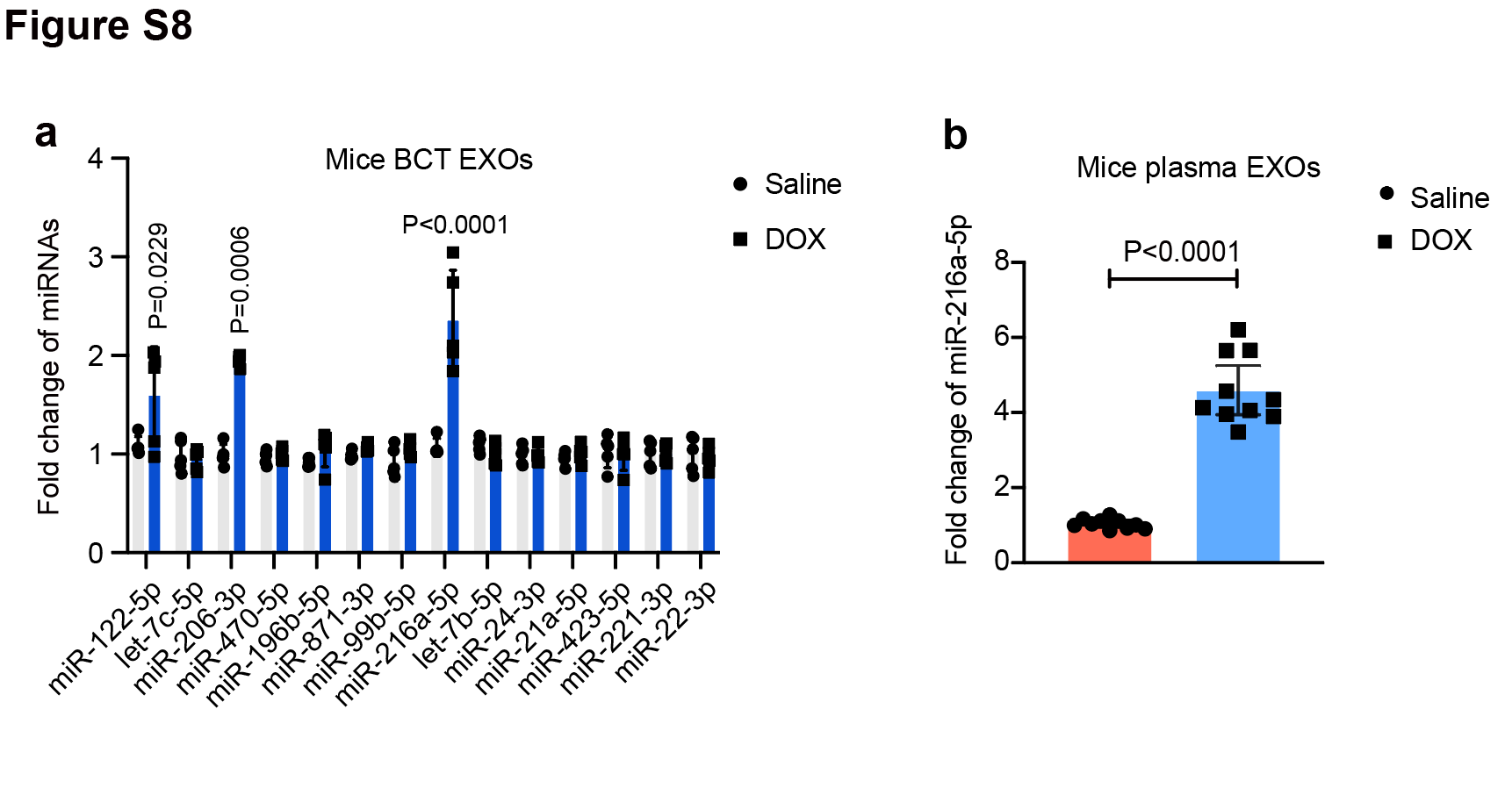


Figure. S8.

**The expression level of miR-216a-5p is increased in mouse plasma EXOs and BCT EXOs. (a)** The top 14 upregulated miRNAs in BC tissue EXOs were detected via qRT‒PCR (n = 5). **(b)** The fold change of miR-216a-5p was measured by qRT-PCR (n = 5). DOX-induced breast cancer EXOs. Data are presented as means ± SD.


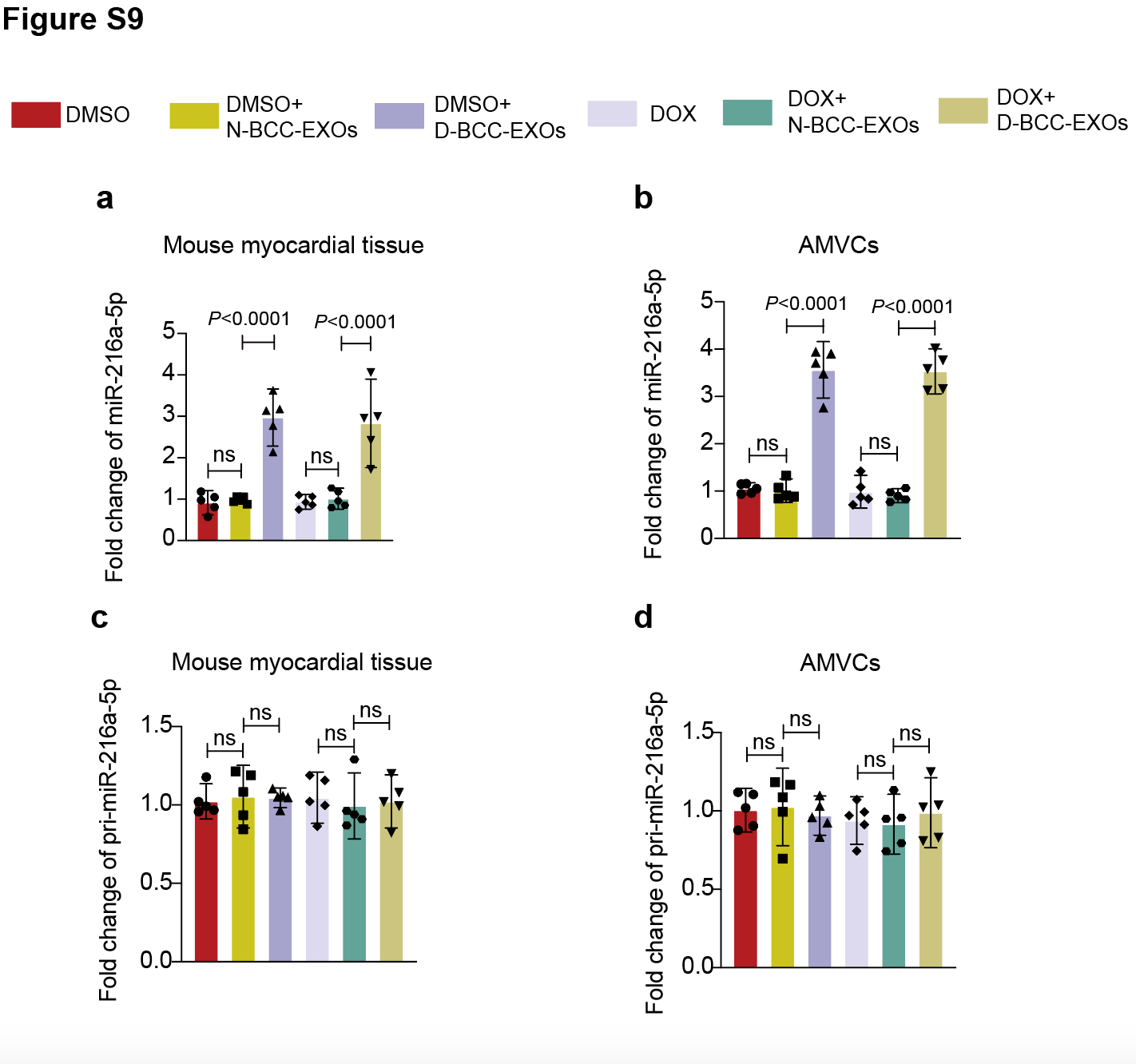


Figure. S9.

**DOX-induced breast cancer cell-EXOs (D-BCC-EXOs) significantly increase miR-216a-5p levels without affecting pri-miR-216a-5p in cardiac tissue and cardiomyocytes.** The fold change of miR-216a-5p was detected via qRT‒PCR in **(a-b)** mouse myocardial tissue and AMVCs (n = 5). **(c-d)** The expression level of miR-216a-5p in mouse myocardial tissue and AMVCs was measured by qRT-PCR (n = 5). “DOX” indicates doxorubicin, “N-BCC-EXOs” indicates normal breast cancer cell EXOs, and “D-BCC-EXOs” indicates DOX-induced breast cancer cell EXOs. Data are presented as means ± SD.


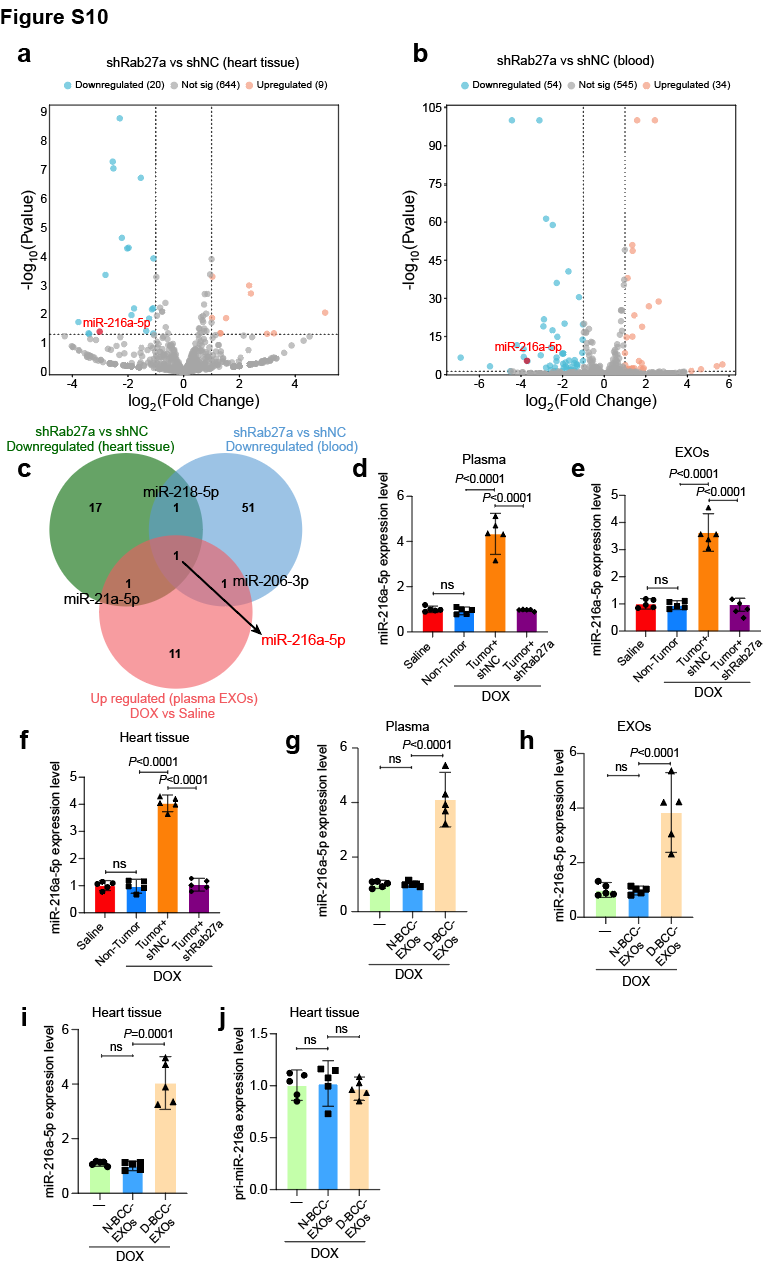


Figure. S10.

**Analysis of miRNA expression profiles in heart tissue and blood before and after administering shRab27 to breast cancer model mice. (a)** Volcano plot showing differentially expressed miRNAs in heart tissue after administering shRab67 to breast cancer model mice, blue dots indicate downregulated miRNAs, red dots indicate upregulated miRNAs, and grey dots represent non-significantly changed miRNAs. **(b)** Volcano plot showing differentially expressed miRNAs in blood samples after administering shRab67 to breast cancer model mice. **(c)** Venn diagram showing the overlap of downregulated miRNAs in heart tissue (green) and blood (blue) and upregulated miRNAs in plasma EXOs (pink). **(d-f)** miR-216a-5p levels in plasma, plasma EXOs, and heart tissue from vehicle, and DOX-treated tumor-bearing mice with or without shRab27a (n = 5). **(g-i)** miR-216a-5p levels in plasma, plasma EXOs, and cardiac tissue from non-tumor-bearing mice injected with EXOs from N-BCC-EXOs or D-BCC-EXOs (n = 5). **(j)** Pri-miR-216a expression in cardiac tissue from mice treated with N-BCC-EXOs or D-BCC-EXOs under DOX exposed (n = 5). “DOX” indicates doxorubicin, “N-BCC-EXOs” indicates normal breast cancer cell EXOs, and “D-BCC-EXOs” indicates DOX-induced breast cancer cell EXOs. Data are presented as means ± SD.


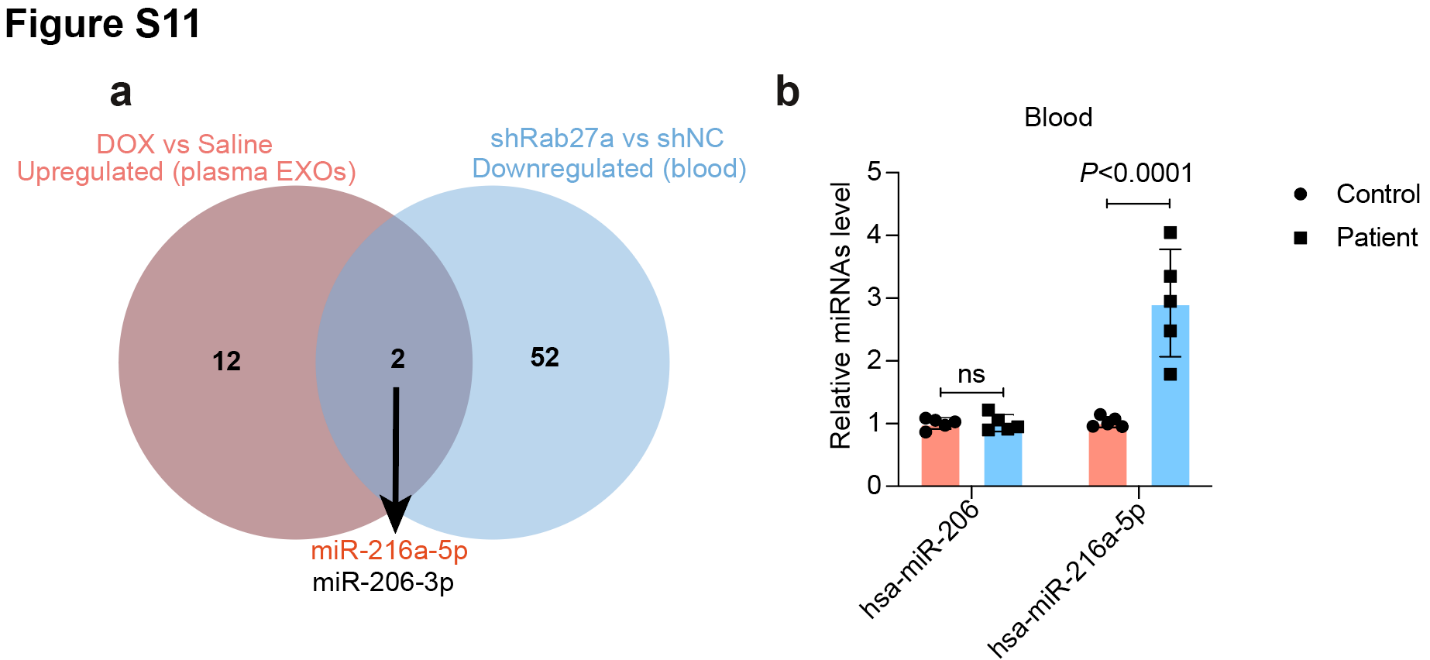


Figure S11.

**miR-216a-5p elevation in breast cancer patients receiving DOX administration. (a)** Venn diagram showing overlapping miRNAs between DOX-induced upregulation in plasma EXOs (red) and Rab27a knockdown-induced downregulation in blood (blue). **(b)** Quantitative PCR analysis of blood miR-206-3p and miR-216a-5p levels in control subjects and breast cancer patients receiving DOX treatment (n = 5). "ns" indicates non-significant, and “DOX” indicates doxorubicin. Data are presented as means ± SD.


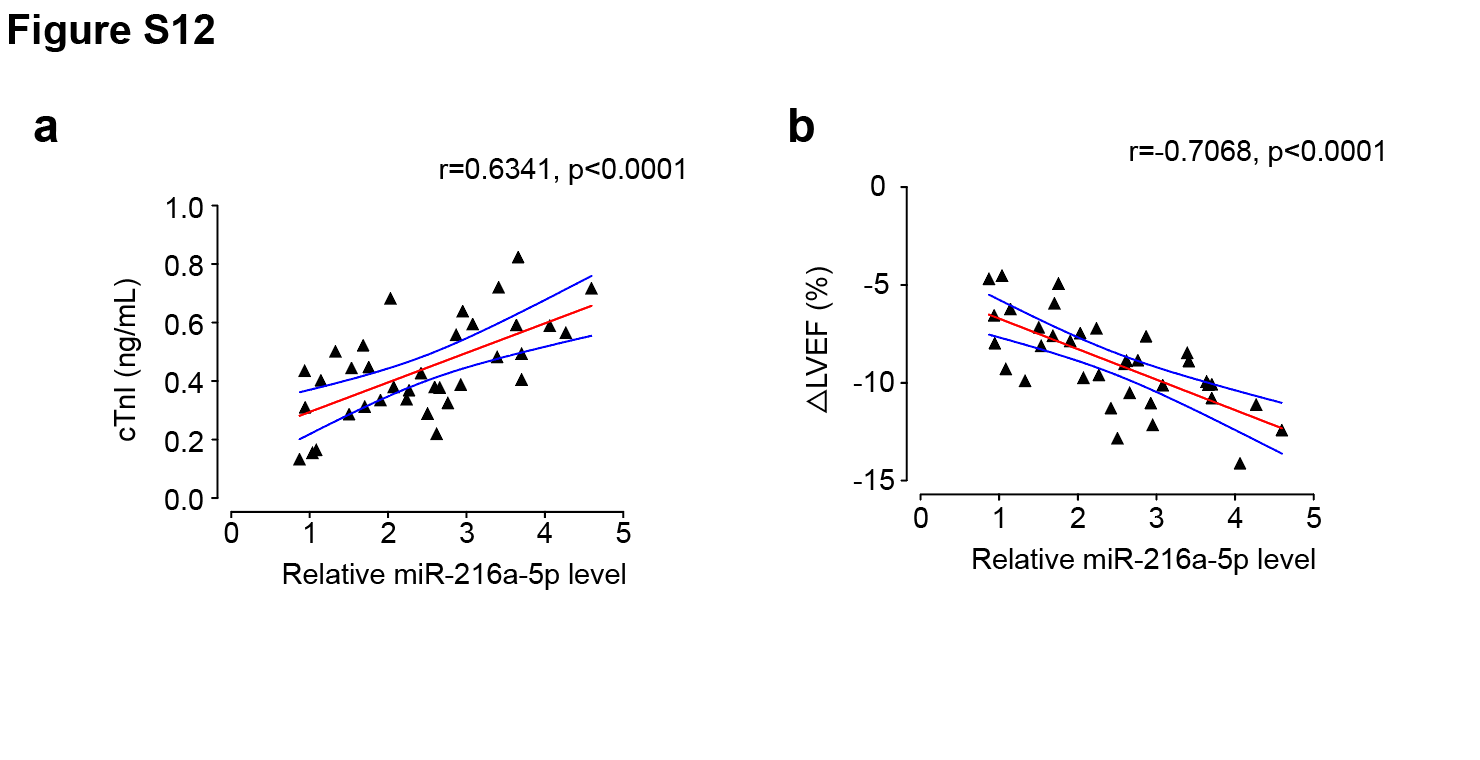


Figure S12.

**Correlation between miR-216a-5p and cardiotoxicity in breast cancer patients receiving DOX treatment.** **(a)** Correlation between miR-216a-5p levels and plasma cTnI (n = 36). **(b)** Correlation between miR-216a-5p levels and ΔLVEF (left ventricular ejection fraction) (n = 36). Red line: linear regression; blue dotted lines: 95% confidence intervals


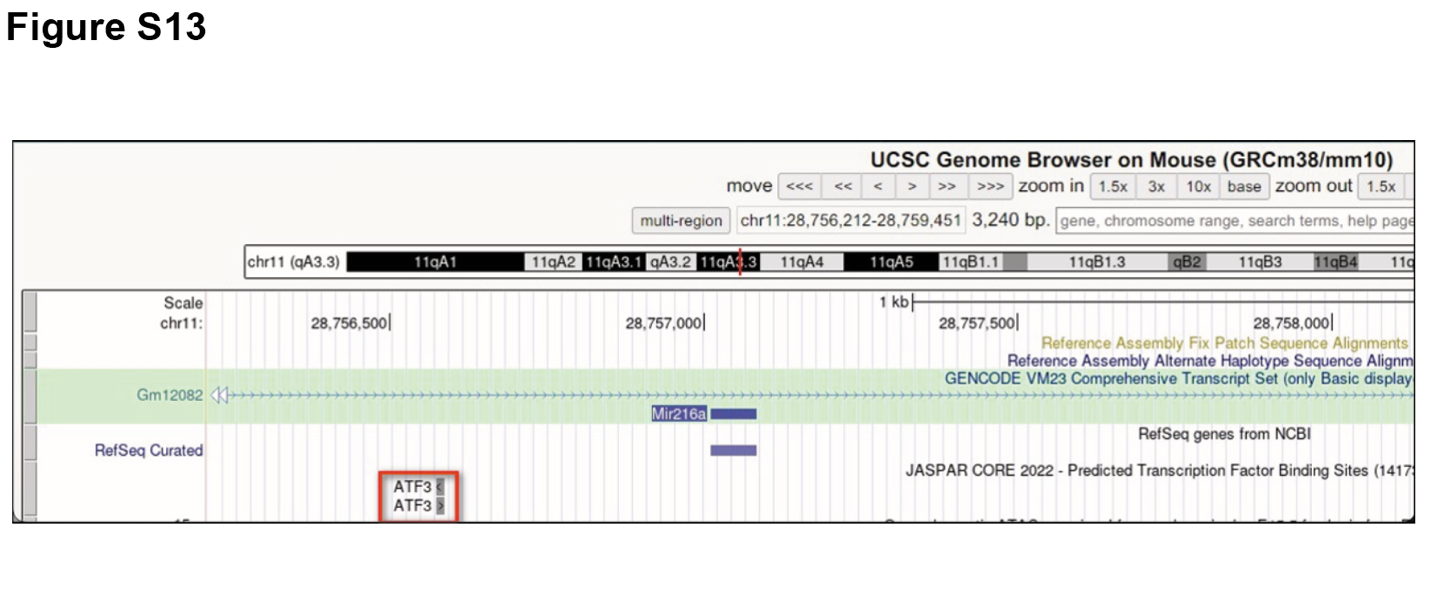


Figure. S13.

**Bioinformatics prediction reveals that the promoter region of pri-miR-216a contains binding sites for ATF3.**


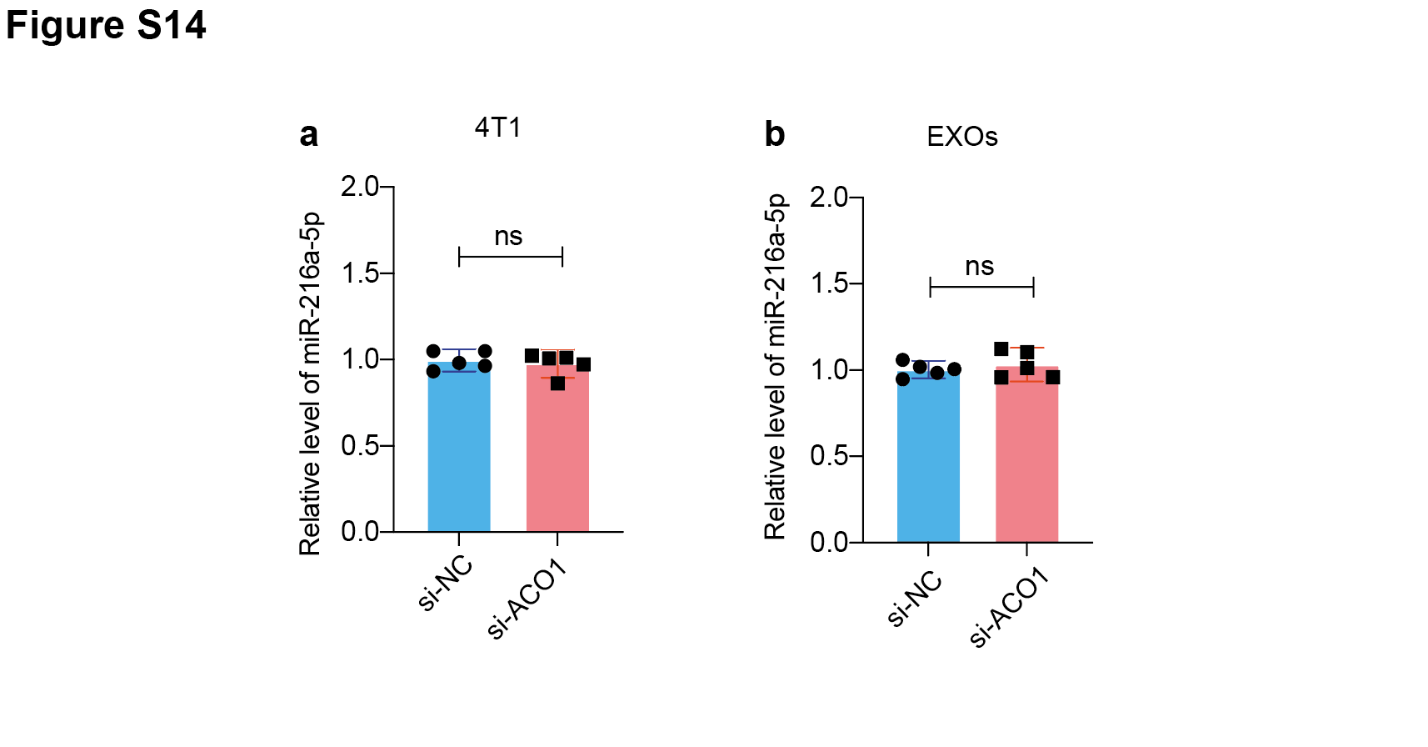


Figure. S14.

**ACO1 knockdown does not affect miR-216a-5p levels in 4T1 cells or EXOs. (a-b)** The expression level of miR-216a-5p in 4T1 cells and EXOs was measured by qRT-PCR (n = 5). “ns” indicates nonsignificant. Data are presented as means ± SD.


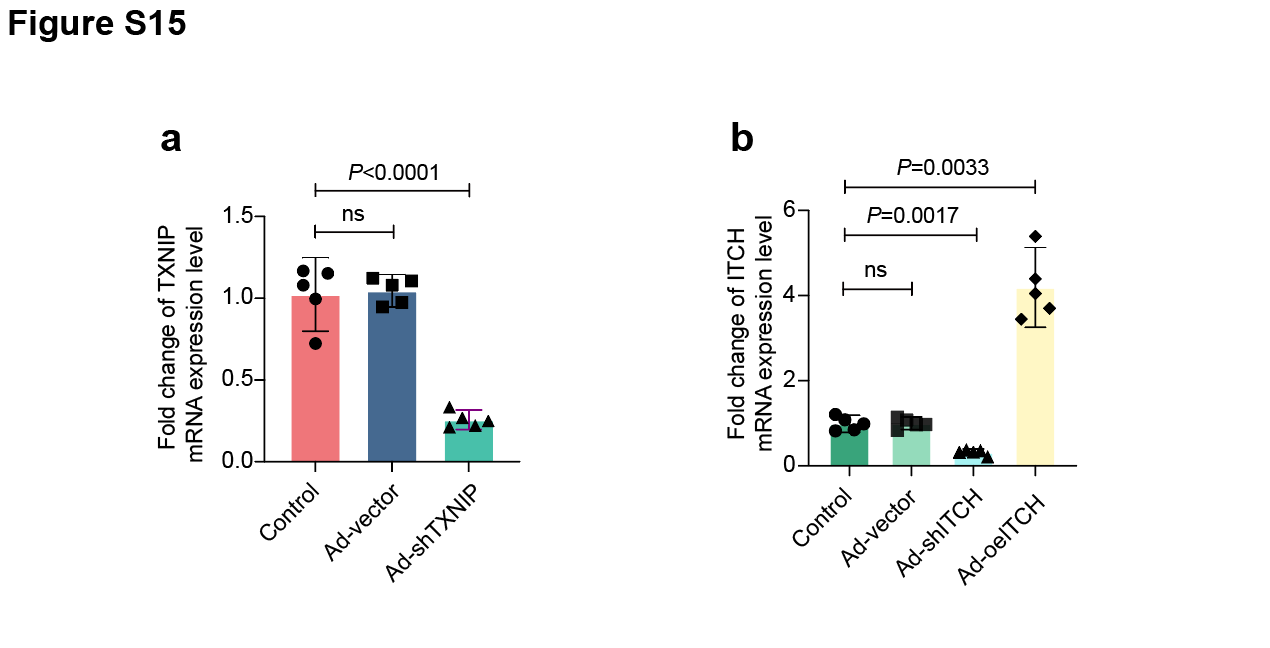


Figure. S15.

**Validation of adenovirus-mediated TXNIP knockdown and ITCH knockdown/overexpression in AMVCs. (a)** The fold change of TXNIP mRNA was detected via qRT‒PCR in AMVCs (n = 5) after adenoviral transduction for 24 hours (n = 5). **(b)** The expression level of ITCH mRNA in AMVCs was measured by qRT-PCR after adenoviral transduction for 24 hours (n = 5). “Ad” indicates adenovirus, “oe” indicates overexpression, and “ns” indicates nonsignificant. Data are presented as means ± SD.


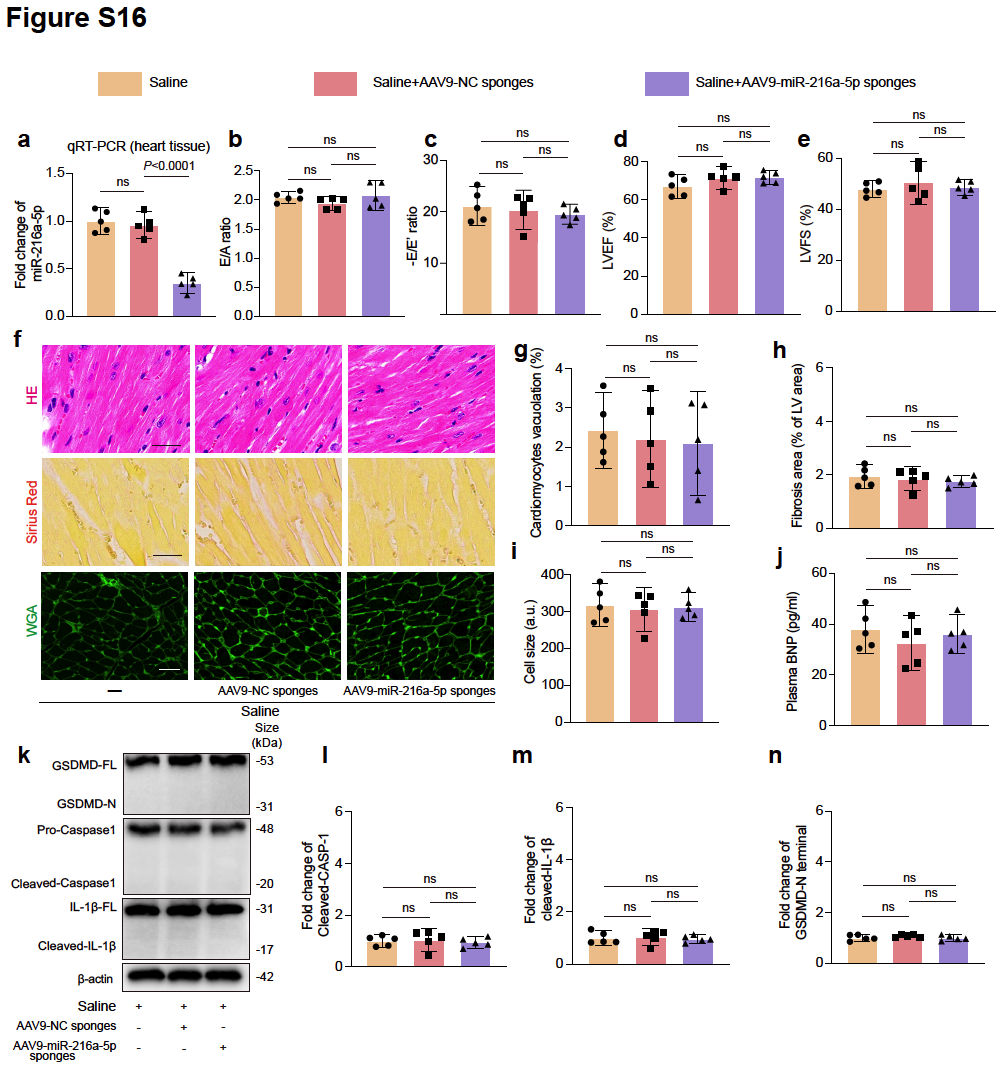


Figure. S16.

**Adeno-associated virus with serotype 9 (AAV9)-mediated miR-216a-5p knockdown does not affect cardiac function and pyroptosis under vehicle conditions.** **(a)** qRT-PCR analysis of cardiac miR-216a-5p expression (n = 5). **(b)** The E/A ratio analysis via Doppler echocardiography (n = 5). **(c)** The -E/E' ratio analysis via Doppler echocardiography (n = 5). **(d-e)** Quantification of the left ventricular ejection fraction (LVEF) and left ventricular fractional shortening (LVFS) via M-mode echocardiography (n = 5). **(f)** Representative images of hematoxylin and eosin (HE) staining, Sirius red staining, and wheat germ agglutinin (WGA) staining. Scale bar: 50 μm. **(g)** Cell vacuolization, **(h)** fibrotic area, and **(i)** cell size was quantified (n = 5). **(j)** Plasma brain natriuretic peptide (BNP) levels were measured. **(k)** Western blot analysis of pyroptosis-related proteins in mouse ventricular tissue. **(l-n)** Quantification of cleaved-caspase-1 (cleaved-CASP1), cleaved-interleukin-1β (cleaved-IL-1β), and gasdermin D N-terminal (GSDMD-N) levels. “E” indicates early diastolic transmitral flow velocity, “E′” indicates early diastolic mitral annular velocity, and “A” indicates late (atrial) diastolic transmitral flow velocity. "ns" indicates nonsignificant. Data are presented as means ± SD.


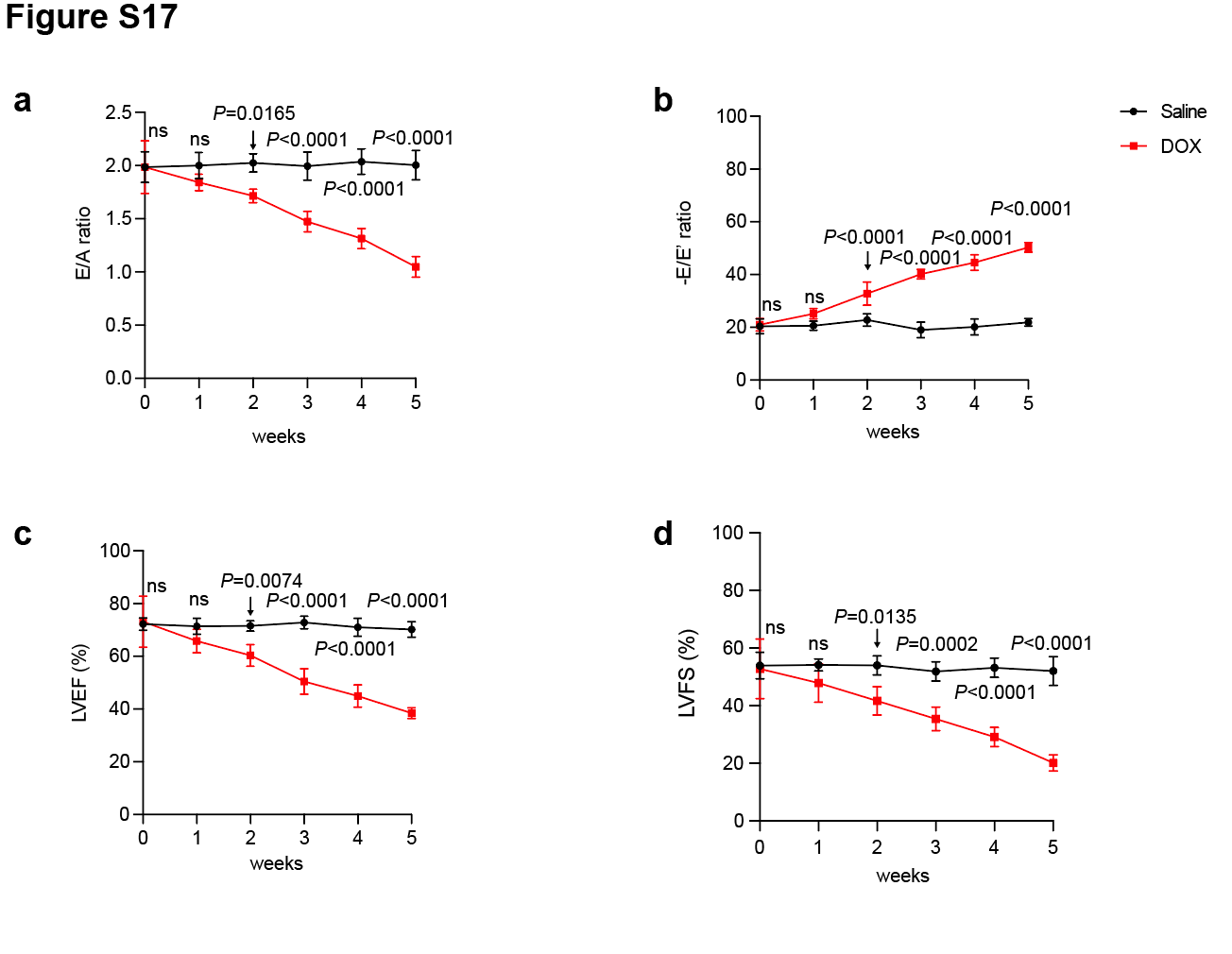


Figure. S17.

**Time course assessment of cardiac function in DOX-treated and vehicle control mice.** **(a)** E/A ratio, **(b)** -E/E' ratio, **(c)** Left ventricular ejection fraction (LVEF), and **(d)** left ventricular fractional shortening (LVFS) ratio were monitored weekly by echocardiography (n = 5). “E” indicates early diastolic transmitral flow velocity, “E′” indicates early diastolic mitral annular velocity, and “A” indicates late (atrial) diastolic transmitral flow velocity. “DOX” indicates doxorubicin, and "ns" indicates non-significant. Data are presented as means ± SD.


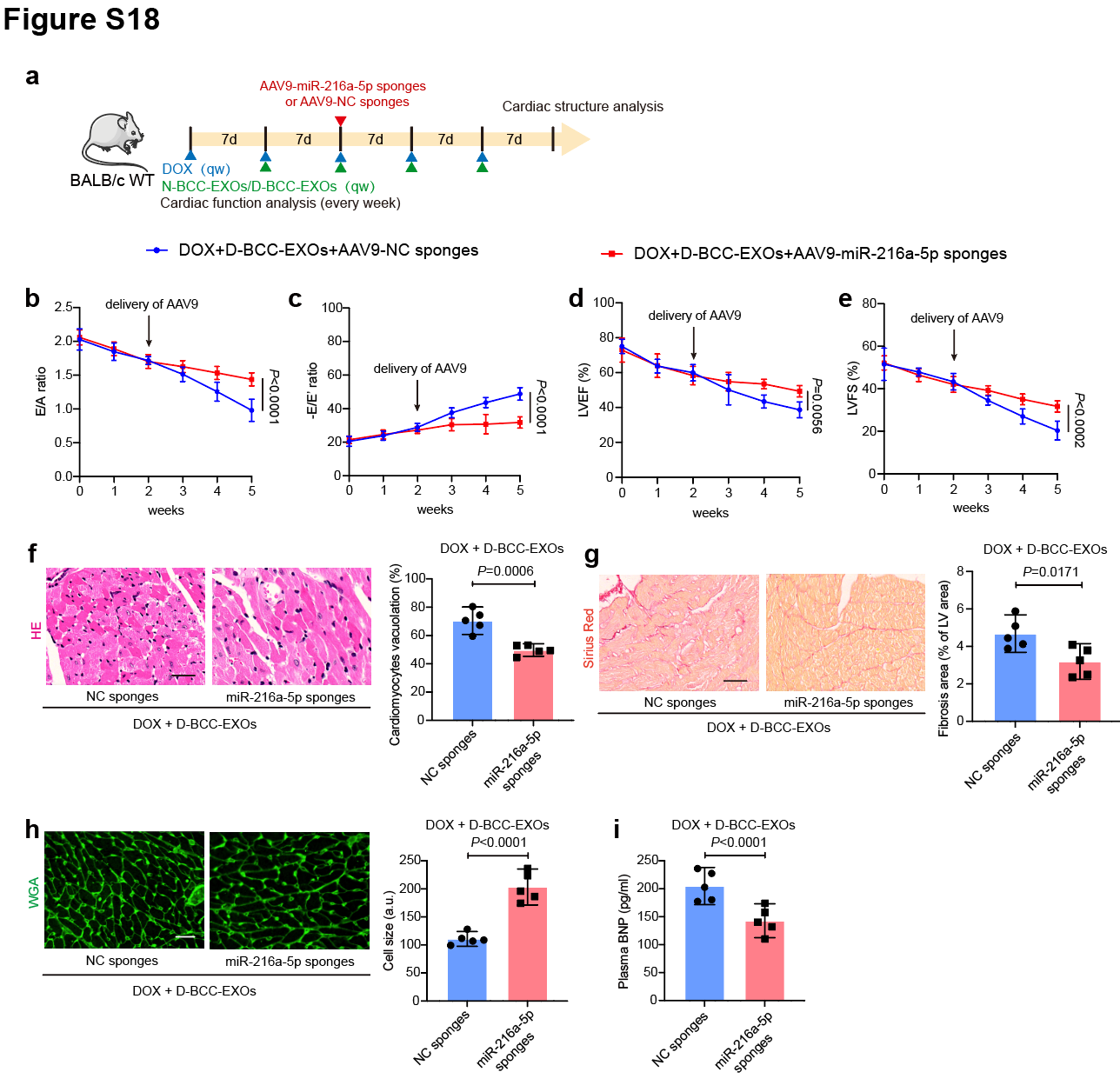


Figure. S18.

**The therapeutic value of AAV9 miR-216a-5p sponge on DOXIC in mice.** **(a)** Schematic diagram showing the treatment protocol of mice treated with DOX and D-BCC-EXOs. AAV9 was delivered at week 2 (n=5). **(b-e)** Sequential monitoring of cardiac function by echocardiography showing E/A ratio, -E/E' ratio, left ventricular ejection fraction (LVEF) and left ventricular fractional shortening (LVFS) in mice, **(f)** Representative image of H&E staining and quantification cardiomyocyte vacuolization. Scale bar: 50 μm. **(g)** Representative image of Sirius red staining and quantification of the fibrotic area. Scale bar: 50 μm. **(h)** Representative wheat germ agglutinin (WGA) staining images and quantification of cardiomyocyte size. Scale bar: 50 μm. **(i)** Plasma brain natriuretic peptide (BNP) was measured by ELISA. “DOX” indicates doxorubicin, “N-BCC-EXOs” indicates normal breast cancer cell EXOs, and “D-BCC-EXOs” indicates DOX-induced breast cancer cell EXOs. Red denotes α-actinin, green denotes CASP1, and blue denotes DAPI. Data are presented as mean ± SD. "ns" indicates non-significant. Data are presented as means ± SD.


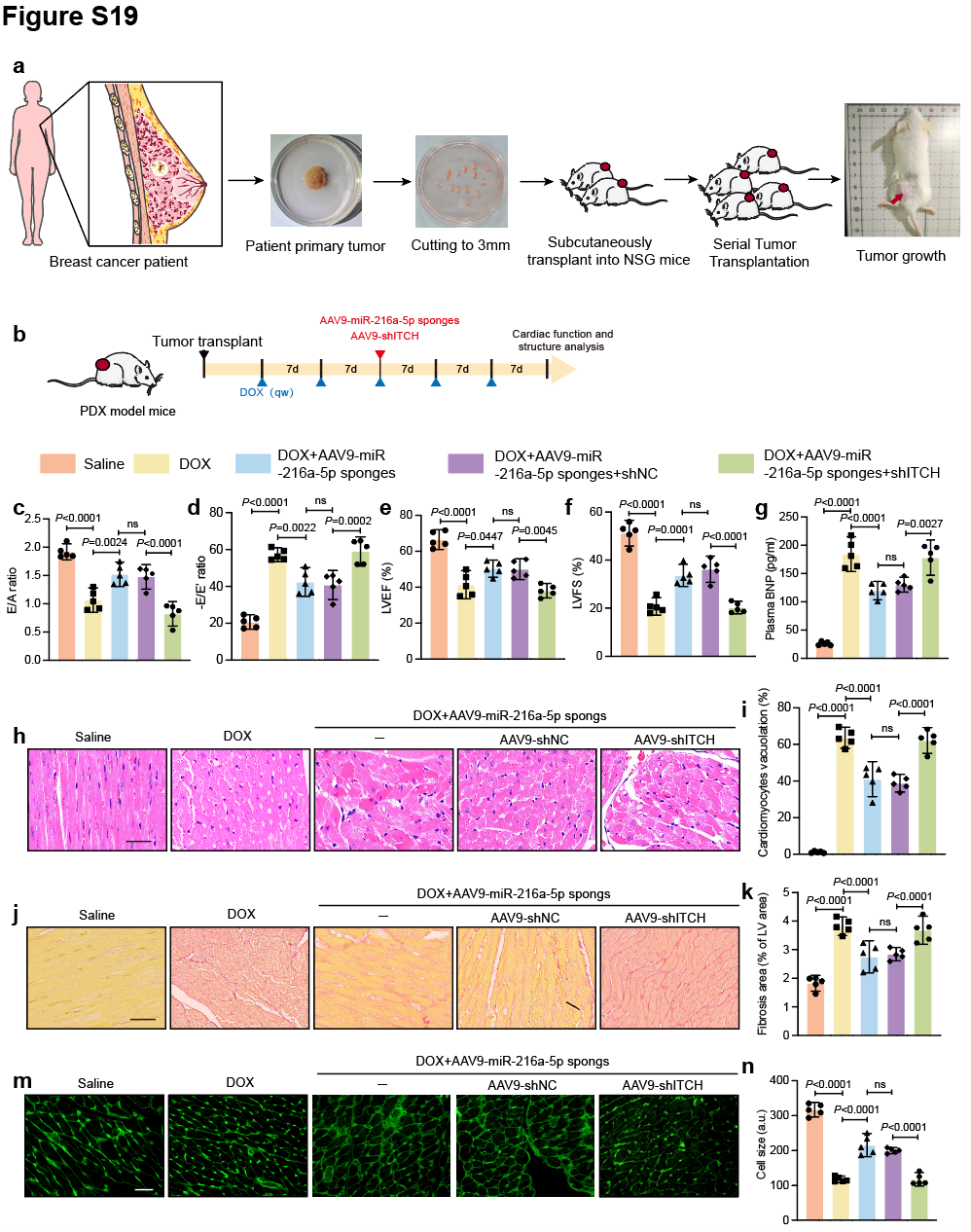


Figure. S19.

**Validation of the therapeutic value of miR-216a-5p/ITCH axis in DOX-induced cardiotoxicity using breast cancer PDX models.** **(a)** Schematic diagram showing the establishment of breast cancer PDX models. Fresh tumor specimens from breast cancer patients were cut into 3mm fragments, subcutaneously transplanted into NSG mice and serially propagated for tumor growth study (n=5). **(b)** Schematic diagram showing the treatment protocol of PDX model mice. **(c-f)** Quantification of cardiac function parameters including E/A ratio, -E/E' ratio, left ventricular ejection fraction (LVEF) and left ventricular fractional shortening (LVFS) by echocardiography (n＝5). **(g)** Plasma brain natriuretic peptide (BNP) levels. **(h-i)** Representative images and quantification of H&E staining showing cardiomyocyte vacuolization. Scale bar: 50 μm. **(j-k)** Representative images and quantification of Sirius red staining indicating cardiac fibrosis. Scale bar: 50 μm. **(m-n)** Wheat germ agglutinin (WGA) staining showing cardiomyocyte size. Scale bar: 50 μm. “DOX” indicates doxorubicin, “E” indicates early diastolic transmitral flow velocity, “E′” indicates early diastolic mitral annular velocity, and “A” indicates late (atrial) diastolic transmitral flow velocity. "ns" indicates non-significant. The schematic diagram part was created using SMART - Servier Medical Art by Servier. Data are presented as mean ± SD.


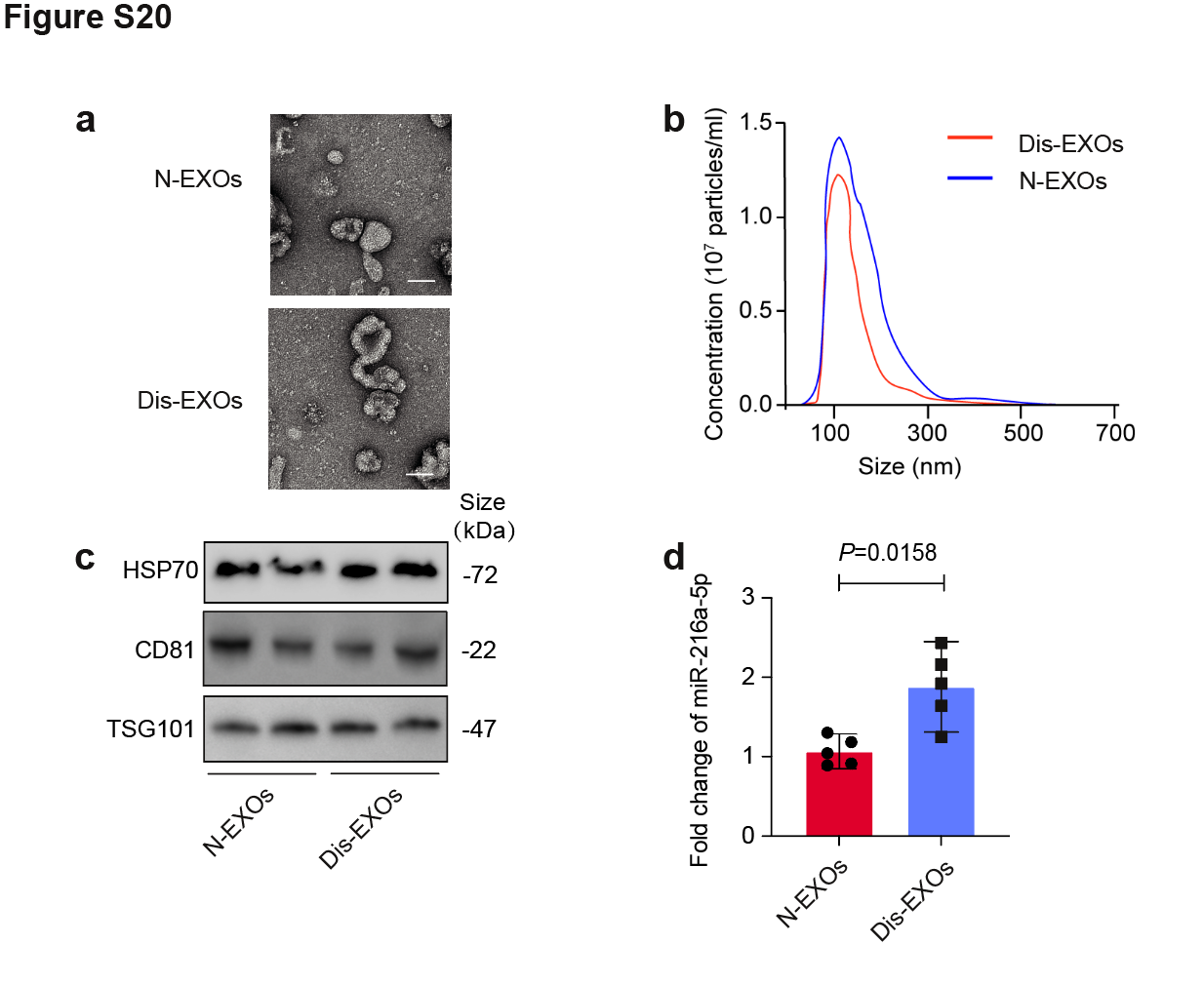


Figure. S20.

**Identification of plasma EXOs from adriamycin cardiomyopathy patients (Dis-EXOs) and healthy donors (N-EXOs). (a)** Plasma EXOs from breast cancer patients were characterized via transmission electron microscopy (TEM). Scale bar: 50 nm. **(b)** NanoSight tracking analysis (NTA) was used to determine the particle size distribution and quantity of plasma EXO particles. **(c)** Western blotting was used to assess the specific endosomal markers HSP70, CD81, and TSG101. **(d)** The expression level of miR-216a-5p in N-EXOs and D-EXOs was measured by qRT-PCR (n = 5). Data are presented as mean ± SD.

Table S1. Upregulated miRNAs in plasma EXOs

| **Gene name** | **Log_2_FC** | **q-value (Benjamini et al. 1995)** |
| --- | --- | --- |
| mmu-miR-216a-5p | 5.807354922 | 0.0000294412 |
| mmu-miR-122-5p | 4.247927513 | 0.0441266016 |
| mmu-miR-206-3p | 3.279106812 | 0.0014558261 |
| mmu-miR-470-5p | 2.949016071 | 0.0291199505 |
| mmu-miR-196b-5p | 2.941897045 | 0.0005169044 |
| mmu-miR-871-3p | 2.814208593 | 0.0171634260 |
| mmu-miR-99b-5p | 2.435490654 | 0.0003385567 |
| mmu-let-7c-5p | 2.264474735 | 0.0000339566 |
| mmu-let-7b-5p | 2.236455465 | 0.0005551904 |
| mmu-miR-24-3p | 1.691049759 | 0.0308207993 |
| mmu-miR-21a-5p | 1.596587826 | 0.0308207993 |
| mmu-miR-423-5p | 1.487938046 | 0.0000001980 |
| mmu-miR-221-3p | 1.383767349 | 0.0000001980 |
| mmu-miR-22-3p | 1.167644061 | 0.0000000010 |

Table S2. Downregulated miRNAs in plasma EXOs

| **Gene name** | **Log_2_FC** | **q-value (Benjamini et al. 1995)** |
| --- | --- | --- |
| mmu-miR-3095-3p | -2 | 0.0207311108 |
| mmu-miR-200c-3p | -1.263034406 | 0.0001816051 |
| mmu-miR-375-3p | -1.378511623 | 0.0000510732 |

Table S3. Downregulated miRNAs in Blood

| **Gene name** | **Log_2_FC** | **q-value (Benjamini et al. 1995)** |
| --- | --- | --- |
| mmu-miR-370-3p | -6.899959811 | 0.0000001962 |
| mmu-miR-409-5p | -5.490455492 | 0.0005243930 |
| mmu-miR-7058-5p | -4.515487179 | 0.0457690800 |
| mmu-miR-183-5p | -4.437343709 | 0.0000000000 |
| mmu-miR-541-5p | -4.197380391 | 0.0000000000 |
| mmu-miR-96-5p | -3.873153049 | 0.0000000001 |
| mmu-miR-133a-3p | -3.869263441 | 0.0000001018 |
| mmu-miR-199b-5p | -3.714506564 | 0.0000035118 |
| mmu-miR-216a-5p | -3.688615280 | 0.0000048412 |
| mmu-miR-206-3p | -3.112903106 | 0.0000000000 |
| mmu-miR-337-5p | -3.050062880 | 0.0000000252 |
| mmu-miR-379-5p | -2.922019868 | 0.0000000000 |
| mmu-miR-199a-5p | -2.895629649 | 0.0000000000 |
| mmu-miR-381-3p | -2.792629931 | 0.0000000000 |
| mmu-miR-195a-3p | -2.792554947 | 0.0018210550 |
| mmu-miR-30c-2-3p | -2.612642492 | 0.0002734720 |
| mmu-miR-329-3p | -2.574805506 | 0.0181100590 |
| mmu-miR-181a-1-3p | -2.557026045 | 0.0000000000 |
| mmu-miR-434-5p | -2.484339390 | 0.0000000000 |
| mmu-miR-1a-3p | -2.472654885 | 0.0000000000 |
| mmu-miR-219a-2-3p | -2.407730543 | 0.0484959250 |
| mmu-miR-152-5p | -2.364509103 | 0.0014012620 |
| mmu-miR-12182-3p | -2.312853958 | 0.0424534750 |
| mmu-miR-300-3p | -2.294312577 | 0.0000049336 |
| mmu-miR-411-5p | -2.291549647 | 0.0000001800 |
| mmu-miR-100-5p | -2.282373180 | 0.0000000000 |
| mmu-miR-182-5p | -2.249975631 | 0.0000000000 |
| mmu-miR-211-5p | -2.043323729 | 0.0406421040 |
| mmu-miR-203-3p | -2.019220966 | 0.0000000051 |
| mmu-miR-34c-5p | -1.972744667 | 0.0000556076 |
| mmu-miR-199b-3p | -1.964555571 | 0.0000000047 |
| mmu-miR-199a-3p | -1.964555571 | 0.0000000047 |
| mmu-miR-409-3p | -1.940816226 | 0.0014060880 |
| mmu-miR-10b-5p | -1.904233120 | 0.0000000000 |
| mmu-miR-434-3p | -1.858637226 | 0.0000167284 |
| mmu-miR-7670-3p | -1.850586795 | 0.0005842870 |
| mmu-miR-362-5p | -1.802391973 | 0.0033770530 |
| mmu-miR-127-3p | -1.718493573 | 0.0000000000 |
| mmu-miR-382-5p | -1.685360807 | 0.0000028008 |
| mmu-miR-214-5p | -1.667681494 | 0.0112286810 |
| mmu-miR-204-5p | -1.606568992 | 0.0012767300 |
| mmu-miR-7043-3p | -1.576683379 | 0.0115276300 |
| mmu-miR-218-5p | -1.444253436 | 0.0249282540 |
| mmu-miR-467a-5p | -1.437179245 | 0.0000255404 |
| mmu-miR-129-5p | -1.418144603 | 0.0000019539 |
| mmu-miR-342-5p | -1.410166458 | 0.0215637120 |
| mmu-miR-9-5p | -1.331311450 | 0.0000926384 |
| mmu-miR-149-5p | -1.315639809 | 0.0006280640 |
| mmu-miR-3535 | -1.292934173 | 0.0001113070 |
| mmu-miR-10a-5p | -1.211962212 | 0.0000000000 |
| mmu-miR-27a-5p | -1.053317061 | 0.0000000025 |
| mmu-miR-148a-3p | -1.037829623 | 0.0000000000 |
| mmu-miR-30e-3p | -1.023034389 | 0.0000036838 |
| mmu-miR-126a-3p | -1.021561470 | 0.0000000000 |

Table S4. Upregulated miRNAs in Blood

| **Gene name** | **Log_2_FC** | **q-value (Benjamini et al. 1995)** |
| --- | --- | --- |
| mmu-miR-217-5p | 5.683619586 | 0.0000980419 |
| mmu-miR-216b-5p | 5.409736980 | 0.0005059200 |
| mmu-miR-378b | 4.653543269 | 0.0075111290 |
| mmu-miR-671-5p | 4.194285278 | 0.0307699560 |
| mmu-miR-375-3p | 2.618403220 | 0.0000000000 |
| mmu-miR-296-5p | 2.436150992 | 0.0000000000 |
| mmu-miR-8114 | 2.147167802 | 0.0000000000 |
| mmu-miR-7672-3p | 1.901925325 | 0.0028750660 |
| mmu-miR-6967-3p | 1.854039549 | 0.0068502560 |
| mmu-miR-574-3p | 1.822699573 | 0.0000000000 |
| mmu-miR-6538 | 1.797521846 | 0.0000303908 |
| mmu-miR-877-5p | 1.751589966 | 0.0036579650 |
| mmu-miR-25-5p | 1.671294628 | 0.0000043338 |
| mmu-miR-92a-3p | 1.585456996 | 0.0000000000 |
| mmu-miR-185-3p | 1.450562605 | 0.0000000000 |
| mmu-miR-6952-3p | 1.449300908 | 0.0032769670 |
| mmu-miR-484 | 1.365474678 | 0.0000000000 |
| mmu-miR-7658-3p | 1.363633429 | 0.0327754890 |
| mmu-miR-425-3p | 1.360938192 | 0.0046091990 |
| mmu-miR-339-5p | 1.355579904 | 0.0000000000 |
| mmu-miR-122-5p | 1.353816420 | 0.0000000000 |
| mmu-miR-331-3p | 1.347755949 | 0.0029216660 |
| mmu-miR-6975-3p | 1.322140525 | 0.0449184390 |
| mmu-miR-1934-3p | 1.292394009 | 0.0450638660 |
| mmu-miR-3057-5p | 1.288956578 | 0.0293774630 |
| mmu-miR-664-5p | 1.271338997 | 0.0076783340 |
| mmu-miR-7063-3p | 1.265547240 | 0.0389713860 |
| mmu-miR-330-3p | 1.239137414 | 0.0217719100 |
| mmu-miR-877-3p | 1.184922945 | 0.0069275980 |
| mmu-miR-664-3p | 1.183127801 | 0.0049412660 |
| mmu-miR-744-5p | 1.125267396 | 0.0000000000 |
| mmu-miR-144-3p | 1.075288497 | 0.0000000000 |
| mmu-miR-5134-3p | 1.051437577 | 0.0059778310 |
| mmu-miR-17-3p | 1.010919058 | 0.0001839511 |

Table S5. Downregulated miRNAs in Myocardial tissue

| **Gene name** | **Log_2_FC** | **q-value (Benjamini et al. 1995)** |
| --- | --- | --- |
| mmu-miR-615-3p | -3.779523469 | 0.0189069470 |
| mmu-miR-18a-5p | -3.415778997 | 0.0460990590 |
| mmu-miR-20b-5p | -3.388036807 | 0.0497534000 |
| mmu-miR-216a-5p | -3.017701221 | 0.0403101390 |
| mmu-miR-375-3p | -2.808516046 | 0.0004380670 |
| mmu-miR-144-3p | -2.548395421 | 0.0000000529 |
| mmu-miR-215-5p | -2.523361267 | 0.0000000907 |
| mmu-miR-451a | -2.296756670 | 0.0000000017 |
| mmu-miR-142a-5p | -2.220889150 | 0.0000229310 |
| mmu-miR-218-5p | -2.025086015 | 0.0000532731 |
| mmu-miR-144-5p | -1.979475242 | 0.0000506099 |
| mmu-miR-224-5p | -1.875023499 | 0.0108622790 |
| mmu-miR-1983 | -1.781705344 | 0.0063530530 |
| mmu-miR-21a-5p | -1.541717880 | 0.0000001915 |
| mmu-miR-101b-3p | -1.343119473 | 0.0377013730 |
| mmu-miR-7a-5p | -1.237813716 | 0.0142351280 |
| mmu-miR-92a-3p | -1.138950633 | 0.0068449620 |
| mmu-miR-146b-5p | -1.097918472 | 0.0062962960 |
| mmu-miR-142a-3p | -1.090689050 | 0.0466945070 |
| mmu-miR-146a-5p | -1.086341982 | 0.0001183700 |

Table S6. Upregulated miRNAs in Myocardial tissue

| **Gene name** | **Log_2_FC** | **q-value (Benjamini et al. 1995)** |
| --- | --- | --- |
| mmu-miR-7032-3p | 5.076787577 | 0.0088543410 |
| mmu-miR-205-5p | 3.236515274 | 0.0461871750 |
| mmu-miR-6944-3p | 2.994912297 | 0.0479403890 |
| mmu-miR-3057-3p | 2.407660570 | 0.0019339820 |
| mmu-miR-470-5p | 2.353793127 | 0.0010202140 |
| mmu-miR-3057-5p | 1.520876481 | 0.0137505900 |
| mmu-miR-871-3p | 1.316351004 | 0.0460683330 |
| mmu-miR-143-5p | 1.027669804 | 0.0005038530 |
| mmu-miR-204-5p | 1.015778851 | 0.0135688630 |

Table S7.

Thirty-nine human miR-216a-5p targets and sixteen conserved putative targets of miR-216a-5p.

| GPBP1L1 | PRPF38B | SSX2IP | RSBN1 | GATAD2B |
| --- | --- | --- | --- | --- |
| F11R | ARID1A | GATAD2B | AGO1 | YBX1 |
| HOOK1 | GFRA1 | CBL | BCAT1 | ITPR2 |
| ERC1 | SLC16A7 | TTC9 | UACA | RANBP10 |
| ZC3H4 | ITCH | PLXNA1 | LPP | BOD1L1 |
| ARHGAP26 | PPARGC1B | AMD1 | PURB | TMEM106B |
| UBE2D4 | CDK14 | MMP16 | MLLT3 | DCAF12 |
| JAK2 | RP2 | BHLHB9 | XIAP |  |

| PLXNA1 | DCAF12 | JAK2 | XIAP | PRPF38B |
| --- | --- | --- | --- | --- |
| RP2 | RSBN1 | RANBP10 | PURB | ITCH |
| ZC3H4 | HOOK1 | SSX2IP | GATAD2B | TTC9 |
| ARID1A |  |  |  |  |

Table S8.

Characteristics of healthy donors and cancer patients.

| Subject | Diagnosis | Age (years) | Sex | LVEF (%) | Cumulative DOX dose | |
| --- | --- | --- | --- | --- | --- | --- |
| 1  2  3  4  5  6  7  8  9  10  11  12  13  14  15  16 | **healthy donor**  **healthy donor**  healthy donor  **healthy donor**  **healthy donor**  **healthy dono**r  healthy donor  healthy donor  breast cancer  **breast cancer**  **breast cancer**  **breast cancer**  **breast cancer**  breast cancer  breast cancer  **breast cancer** | **49**  **41**  67  **46**  **59**  **59**  43  52  64  **51**  **59**  **69**  **41**  49  47  **52** | **female**  **female**  female  **female**  **female**  **female**  female  female  female  **female**  **female**  **female**  **female**  female  female  **female** | **69**  **66**  70  **69**  **65**  **64**  68  65  53  **45**  **44**  **42**  **43**  51  50  **44** | -  -  -  -  -  -  -  -  440 mg/m^2^  **550 mg/m^2^**  **580 mg/m^2^**  **570 mg/m^2^**  **540 mg/m^2^**  360 mg/m^2^  420 mg/m^2^  **530 mg/m^2^** |  |

Table S9.

qRT-PCR (Real-Time Quantitative Reverse Transcription PCR) primer and other nucleotide sequences used in this study.

| 1. Primers for qRT-PCR | | |
| --- | --- | --- |
| Name | Sequence (5’-3’) | |
| mmu-miR-216a-5p reverse | GTCGTATCCAGTGCAGGGT | |
| mmu-miR-216a-5p forward | AACAAGTTGGTTTAATCTCAGCTGG | |
| mmu-miR-122-5p reverse | GTCGTATCCAGTGCAGGGT | |
| mmu-miR-122-5p forward | AACAAGAGCTGTGGAGTGTGA | |
| mmu-miR-470-5p reverse | GTCGTATCCAGTGCAGGGT | |
| mmu-miR-470-5p forward | AACAAGCAGTGCTCTTCTTGG | |
| mmu-miR-196b-5p reverse | GTCGTATCCAGTGCAGGGT | |
| mmu-miR-196b-5p forward | AACAAGAACTGGTCGGTGATTTAG | |
| mmu-miR-871-3p reverse | GTCGTATCCAGTGCAGGGT | |
| mmu-miR-871-3p forward | AACAAGTGCAGTGCTCTATTCAG | |
| mmu-miR-99b-5p reverse | GTCGTATCCAGTGCAGGGT | |
| mmu-miR-99b-5p forward | AATATTAGGCACCCACCCGTAG | |
| mmu-let-7b-5p reverse | GTCGTATCCAGTGCAGGGT | |
| mmu-let-7b-5p forward | AACAAGGCAGGGTGAGGTAG | |
| mmu-let-7c-5p reverse | GTCGTATCCAGTGCAGGGT | |
| mmu-let-7c-5p forward | AACAAGTGTGTGCATCCGG | |
| mmu-miR-24-3p reverse | GTCGTATCCAGTGCAGGGT | |
| mmu-miR-24-3p forward | AACAAGCTCCGGTGCCTAC | |
| mmu-miR-21a-5p reverse | GTCGTATCCAGTGCAGGGT | |
| mmu-miR-21a-5p forward | AACAAGTGTACCACCTTGTCG | |
| mmu-miR-423-5p reverse | GTCGTATCCAGTGCAGGGT | |
| mmu-miR-423-5p forward | AACACGCACTTGTGAGGAAATAAA | |
| mmu-miR-221-3p reverse | GTCGTATCCAGTGCAGGGT | |
| mmu-miR-221-3p forward | AACAAGATCCAGGTCTGGGG | |
| mmu-miR-22-3p reverse | GTCGTATCCAGTGCAGGGT | |
| mmu-miR-22-3p forward | AACAAGACCTGGCTGAGCC | |
| mmu-miR-206-3p reverse | GTCGTATCCAGTGCAGGGT | |
| mmu-miR-206-3p forward | AACAATCCAGGCCACATGC | |
| PLXNA1 forward | ACCCACCTAGTGGTGCATGA | |
| PLXNA1 reverse | CGGTTAGCGGCATAGTCCA | |
| DCAF12 forward | ATGGCCCGGAAAGTAGTTAGC | |
| DCAF12 reverse | ACTTCCCGGTTCTTCAAGTAGT | |
| JAK2 forward | TCTGGGGAGTATGTTGCAGAA | |
| JAK2 reverse | AGACATGGTTGGGTGGATACC | |
| XIAP forward | ACCGTGCGGTGCTTTAGTT | |
| XIAP reverse | TGCGTGGCACTATTTTCAAGAT | |
| PRPF38B forward | AACATCCTGTCGTCGCCTTAC | |
| PRPF38B reverse | ACGTGCGTGACCTTAAAGTAGA | |
| RP2 forward | TTCTTCTCCAAGAGACGGAAGG | |
| RP2 reverse | GGTAAGCGACCTACTGTTTCATC | |
| RSBN1 forward | CGATCTCAAGCACAAGGACAA | |
| RSBN1 reverse | 5’-ATCGGCTCTCTGGGTTTTACC | |
| RANBP10 forward | AACCTCCGCGTCCACTACA | |
| RANBP10 reverse | CCTTGAGCCGAGAGTCCTATT | |
| PURB forward | CATCCGCCAAACGGTCAAC | |
| PURB reverse | TAGTCGTCTATGAGCTTCGCC | |
| ITCH forward | TGATGATGGCTCCAGATCCAA | |
| ITCH reverse | GACTCTCCTATTTTCACCAGCTC | |
| ZC3H4 forward | AGAAGCCGGAAAGAAAAGGGG | |
| ZC3H4 reverse | GGCTGAAATCCGAGTCATCTGA | |
| HOOK1 forward | CAGACATTCAATACTGCCTCACC | |
| HOOK1 reverse | CCCCAACATCCTCTTTAATTCGG | |
| SSX2IP forward | CTGGAGGACTGGTAAAACTGAAG | |
| SSX2IP reverse | CTGCTTAGTTCCCCGGCAT | |
| GATAD2B forward | GATGCTCTTCGCTTGAATCTGT | |
| GATAD2B reverse | GAGTCGCTTTGCCAGGACAT | |
| TTC9 forward | ACGAGCGCACGAGTTCAAAA | |
| TTC9 reverse | TATTTGCCTATGGCTTCACGG | |
| ARID1A forward | CCTGAAGAACTCGAACGGGAA | |
| ARID1A reverse | TCCGCCATGTTGTTGGTGG | |
| ATF3 forward | CCTCTGCGCTGGAATCAGTC | |
| ATF3 reverse | TTCTTTCTCGTCGCCTCTTTTT | |
| GAPDH-forward | CAATGACCCCTTCATTGACC | |
| GAPDH-reverse | TTGATTTTGGAGGGATCTCG | |
| 1. miRNA inhibitor and mimics sequences (5'-3') | | |
| miR-216a-5p mimic | UAAUCUCAGCUGGCAACUGUGA |  |
| miR-216a-5p inhibitor | UCACAGUUGCCAGCUGAGAUUA |  |
| miR-122-5p mimic | UGGAGUGUGACAAUGGUGUUUG |  |
| miR-206-3p mimic | UGGAAUGUAAGGAAGUGUGUGG |  |
| miR-470-5p mimic | UUCUUGGACUGGCACUGGUGAGU |  |
| miR-196b-5p mimic | UAGGUAGUUUCCUGUUGUUGGG |  |
| miR-871-3p mimic | UGACUGGCACCAUUCUGGAUAAU |  |
| miR-99b-5p mimic | CACCCGUAGAACCGACCUUGCG |  |
| Let-7c-5p mimic | UGAGGUAGUAGGUUGUAUGGUU |  |
| Let-7b-5p mimic | UGAGGUAGUAGGUUGUGUGGUU |  |
| miR-24-3p mimic | UGGCUCAGUUCAGCAGGAACAG |  |
| miR-21a-5p mimic | UAGCUUAUCAGACUGAUGUUGA |  |
| miR-423-5p mimic | UGAGGGGCAGAGAGCGAGACUUU |  |
| miR-216a-5p mimic | UAAUCUCAGCUGGCAACUGUGA |  |
| miR-216a-5p inhibitor | UCACAGUUGCCAGCUGAGAUUA |  |
| miR-122-5p mimic | UGGAGUGUGACAAUGGUGUUUG |  |
| miR-206-3p mimic | UGGAAUGUAAGGAAGUGUGUGG |  |
| miR-470-5p mimic | UUCUUGGACUGGCACUGGUGAGU |  |
| miR-196b-5p mimic | UAGGUAGUUUCCUGUUGUUGGG |  |
| miR-871-3p mimic | UGACUGGCACCAUUCUGGAUAAU |  |
| miR-99b-5p mimic | CACCCGUAGAACCGACCUUGCG |  |
| Let-7c-5p mimic | UGAGGUAGUAGGUUGUAUGGUU |  |
| Let-7b-5p mimic | UGAGGUAGUAGGUUGUGUGGUU |  |
| miR-24-3p mimic | UGGCUCAGUUCAGCAGGAACAG |  |
| miR-21a-5p mimic | UAGCUUAUCAGACUGAUGUUGA |  |
| miR-423-5p mimic | UGAGGGGCAGAGAGCGAGACUUU |  |
| miR-221-3p mimic | AGCUACAUUGUCUGCUGGGUUUC |  |
| miR-22-3p mimic | AAGCUGCCAGUUGAAGAACUGU |  |
| miR-NC | UUUGUACUACACAAAAGUACUG |  |
| 1. ShRNA and its negative control (NC) sequences (5'-3') | | |
| sh NC | CCTAAGGTTAAGTCGCCCTCG-TTCAAGAGA CGAGGGCGACTTAACCTTAGG-TTTTTT | |
| sh ITCH | ACATGCCATCTACCGTCATTA- TTCAAGAGA- TAATGACGGTAGATGGCATGT-TTTTTT | |
| sh TXNIP | AGCATCTGTATTAGCGCATTT-TTCAAGAGA-AAATGCGCTAATACAGATGC-TTTTTTT | |
| sh Rab27 | CCGGGCTTCTGTTCGACCTGACAAACTCGAGTTTGTCAGGTCGAACAGAAGCTTTTT | |
| 1. SiRNA and its negative control (NC) sequences (5'-3') | | |
| si NC | GACGTCTGGCGATGCAAGAGT |  |
| si SF3B4 | GAAUGAAGAUCUCAGCAAAGC |  |
| si ACO1 | CAGUGAAUUGGAAGUGUAAGC |  |
| si ATF3 | GGGCGAUAAUGAUGUGUCAAA |  |
| 1. Biotin-coupled probe pull down assay probe sequences (5'-3') | | |
| miR-216a-5p | Biotin-TCACAGTTGCCAGCTGAGATTA |  |
| miR-216a-5p mut | Biotin-AGTGTGTTGCCAGCTGAGATTA |  |
| 1. Electrophoretic mobility shift assay probe sequences (5'-3') | | |
| miR-216a-5p | TAATCTCAGCTGGCAACTGTGA-Biotin |  |
| miR-216a-5p mut 1 | TAATCTCAGCTGGCAAATGTGA-Biotin |  |
| miR-216a-5p mut 2 | TAATCTCAGCTGGCAACACACT-Biotin |  |
