# Supplementary figures and images for: Exosomal transfer of pro-pyroptotic miR-216a-5p exacerbates anthracycline cardiotoxicity through breast cancer-heart pathological crosstalk
Source: Signal Transduct Target Ther. 2025 May 14;10:157. doi: 10.1038/s41392-025-02245-4 (PMC12075849; doi:10.1038/s41392-025-02245-4)

**Original blots of immunoblots**


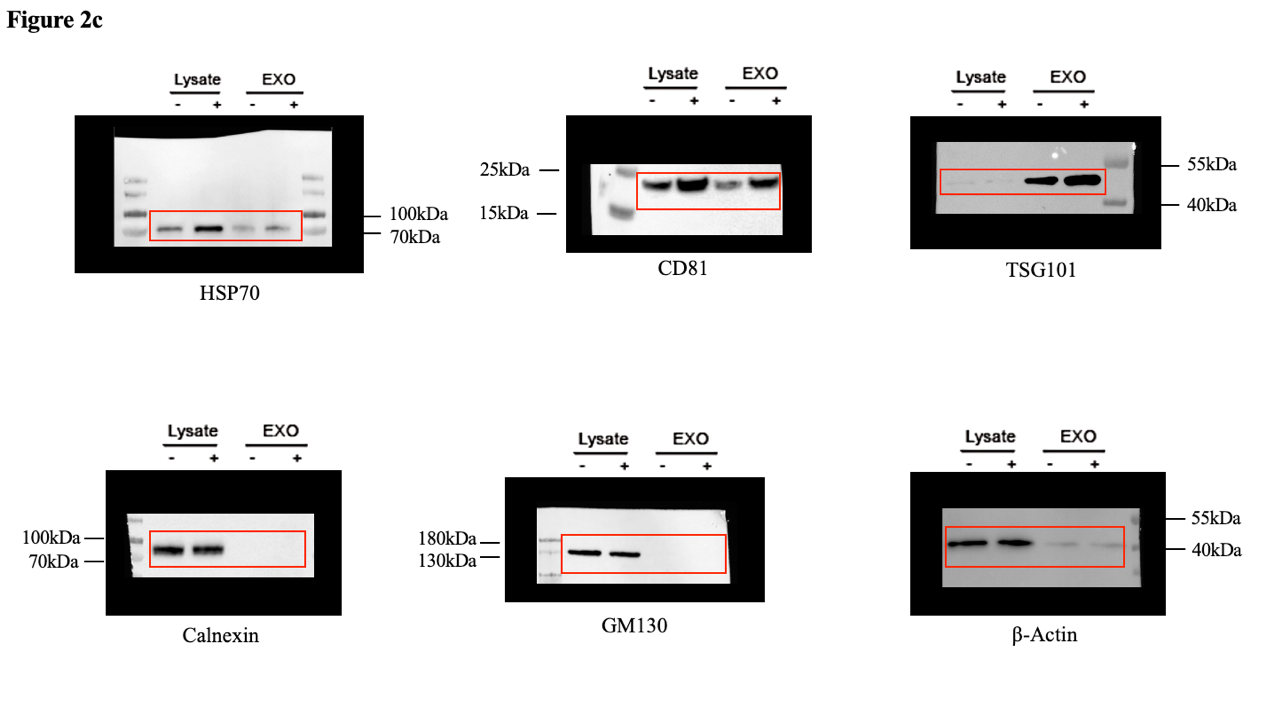


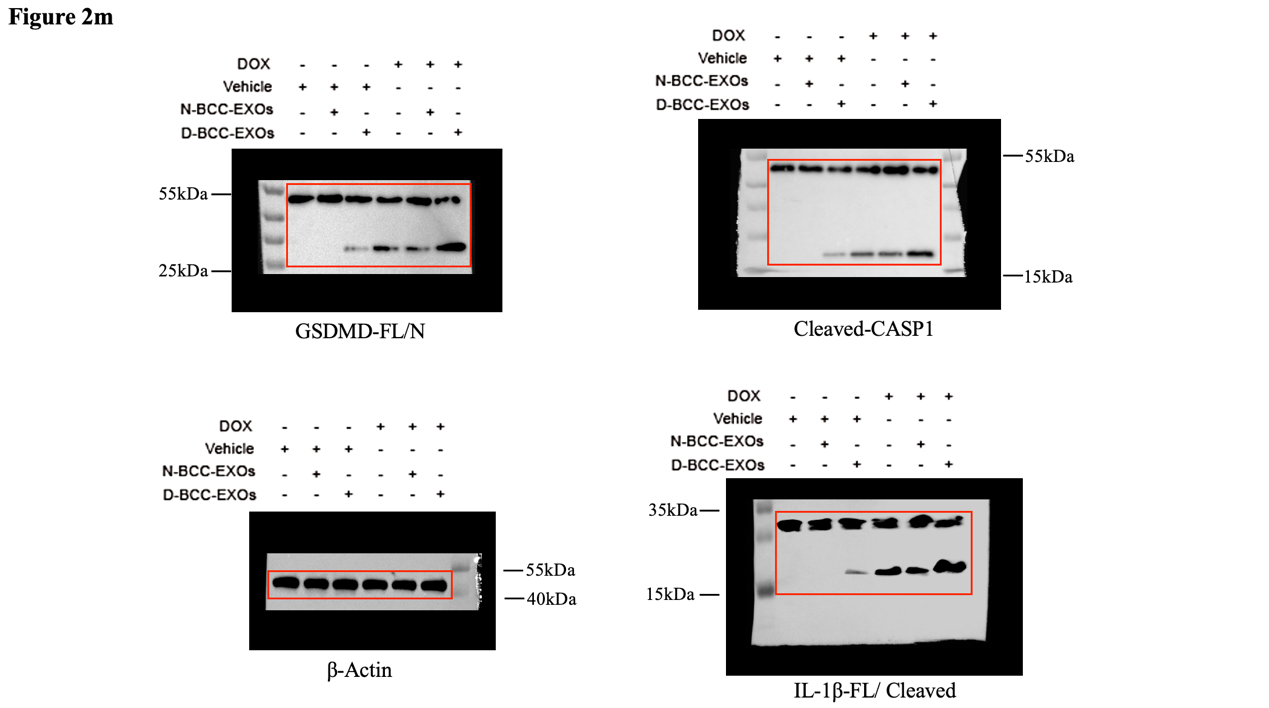


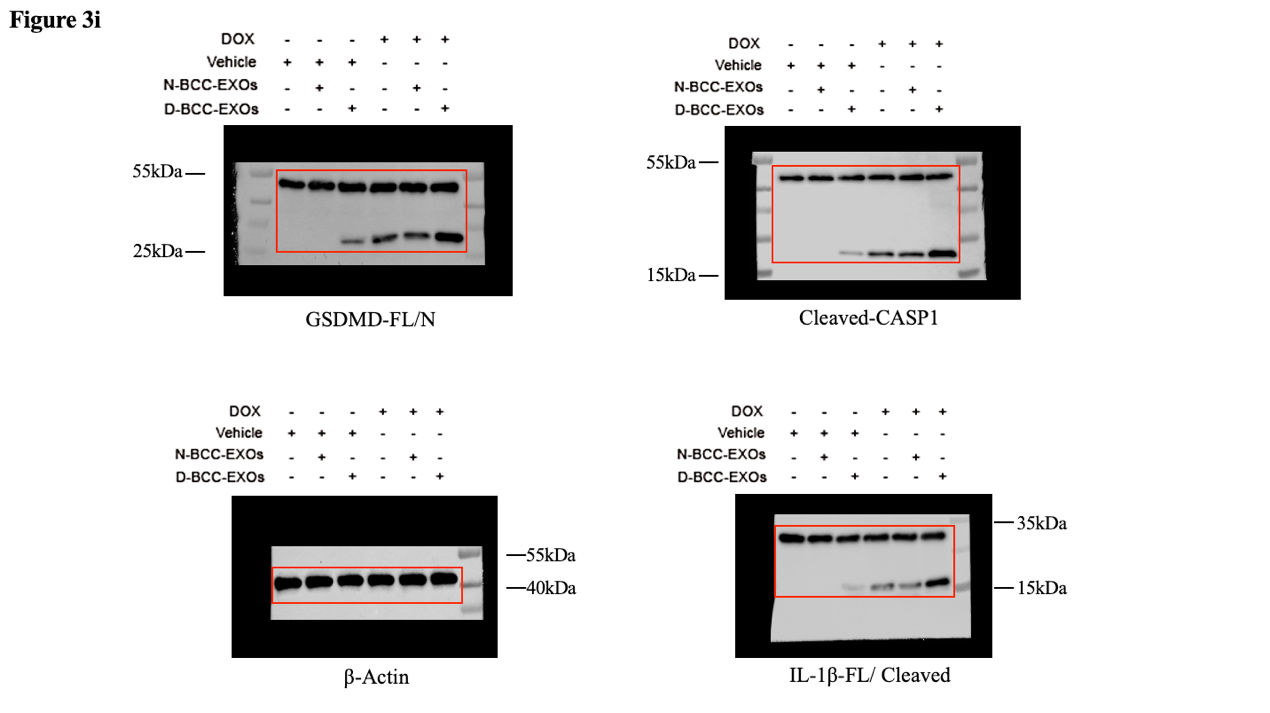


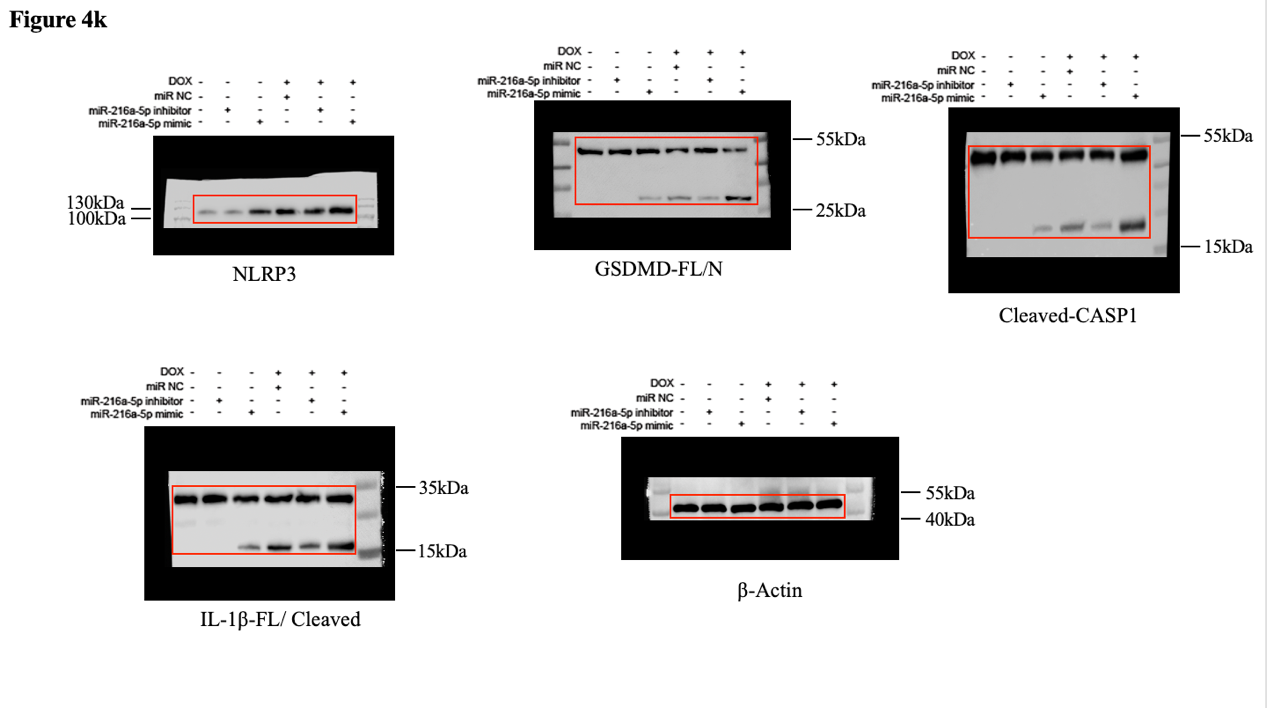

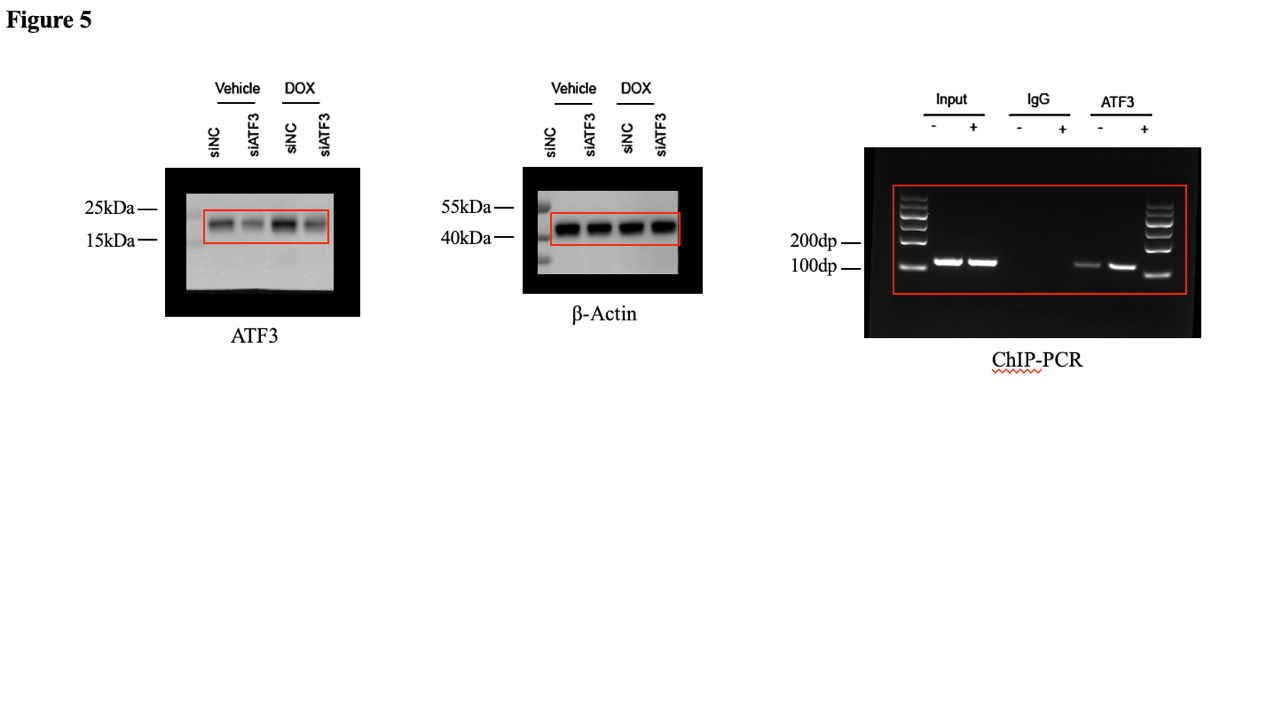


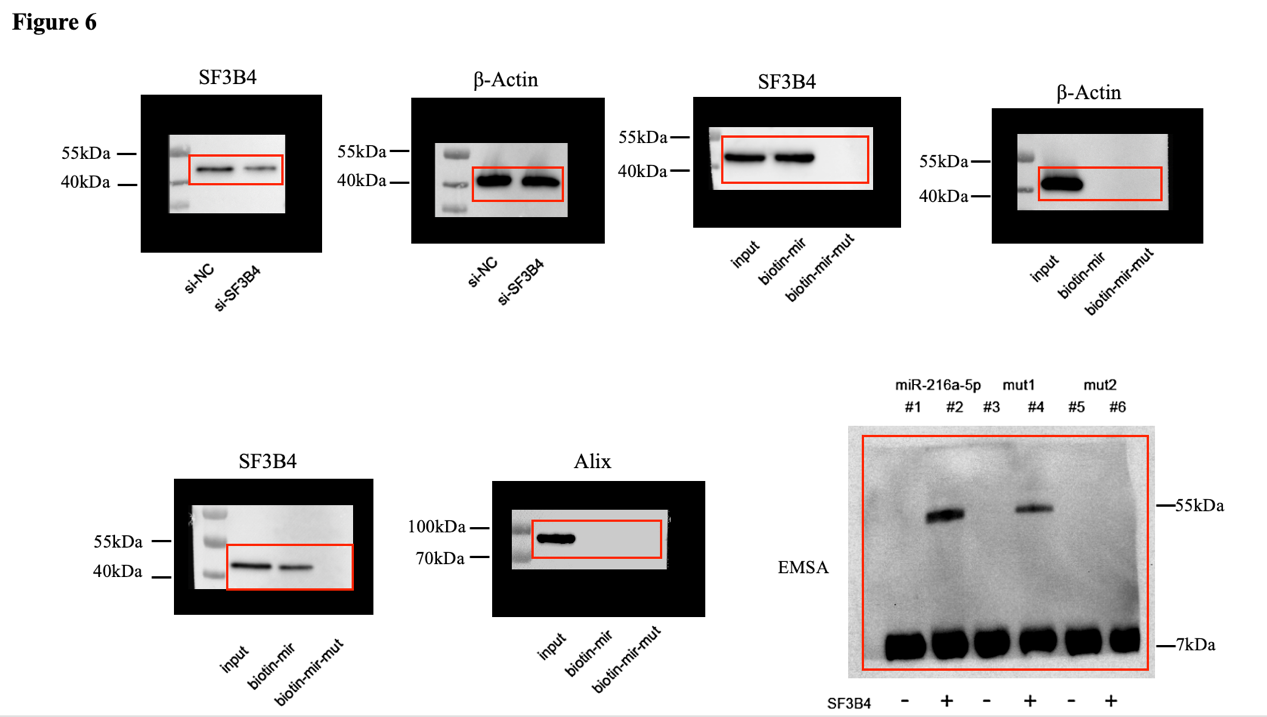


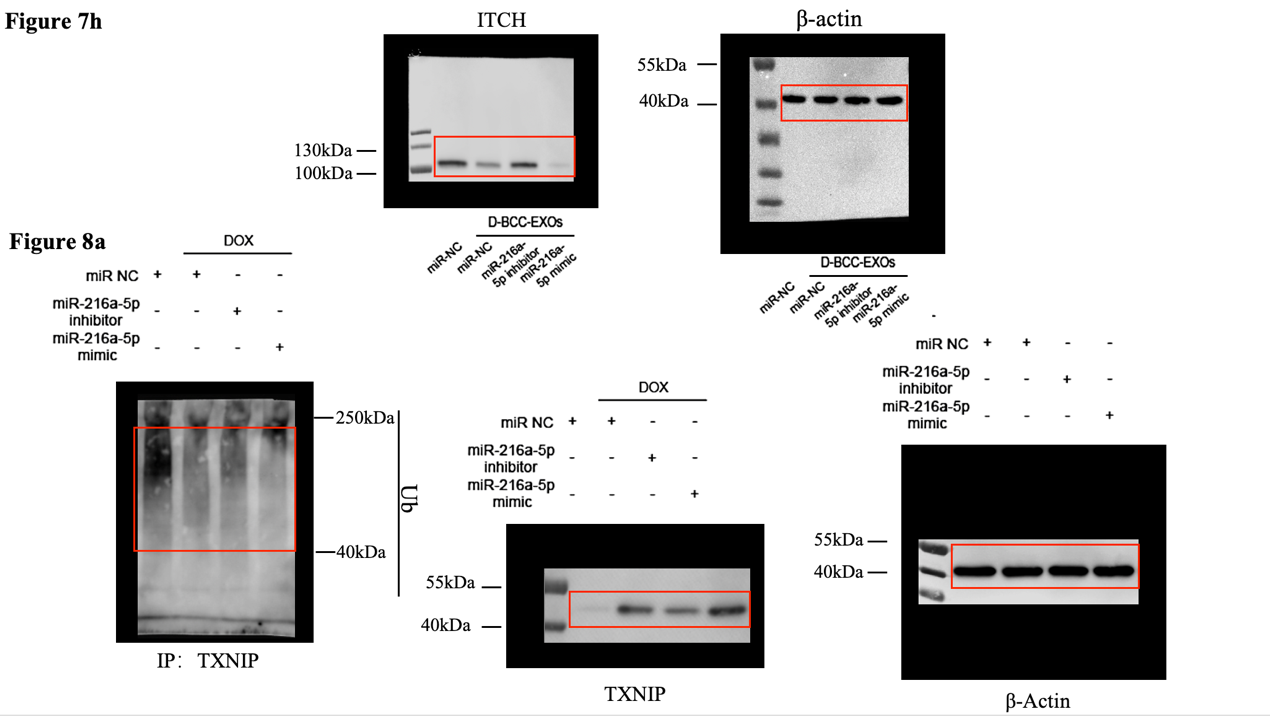


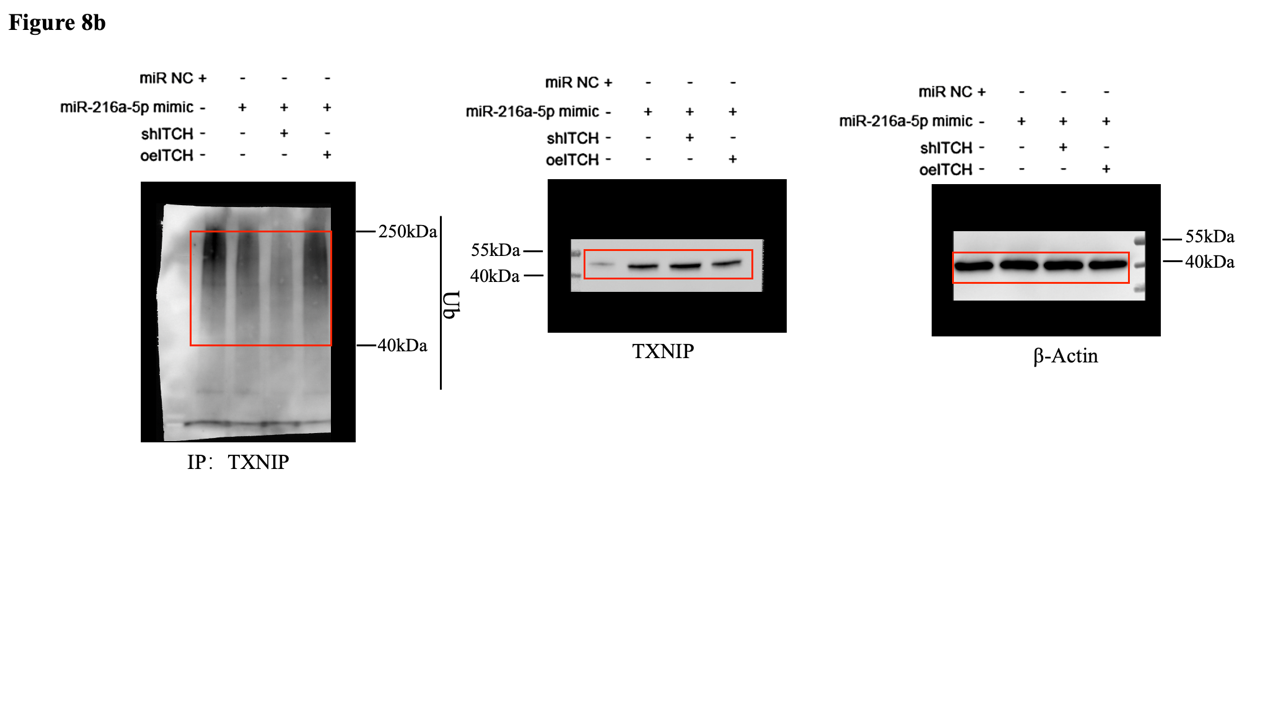


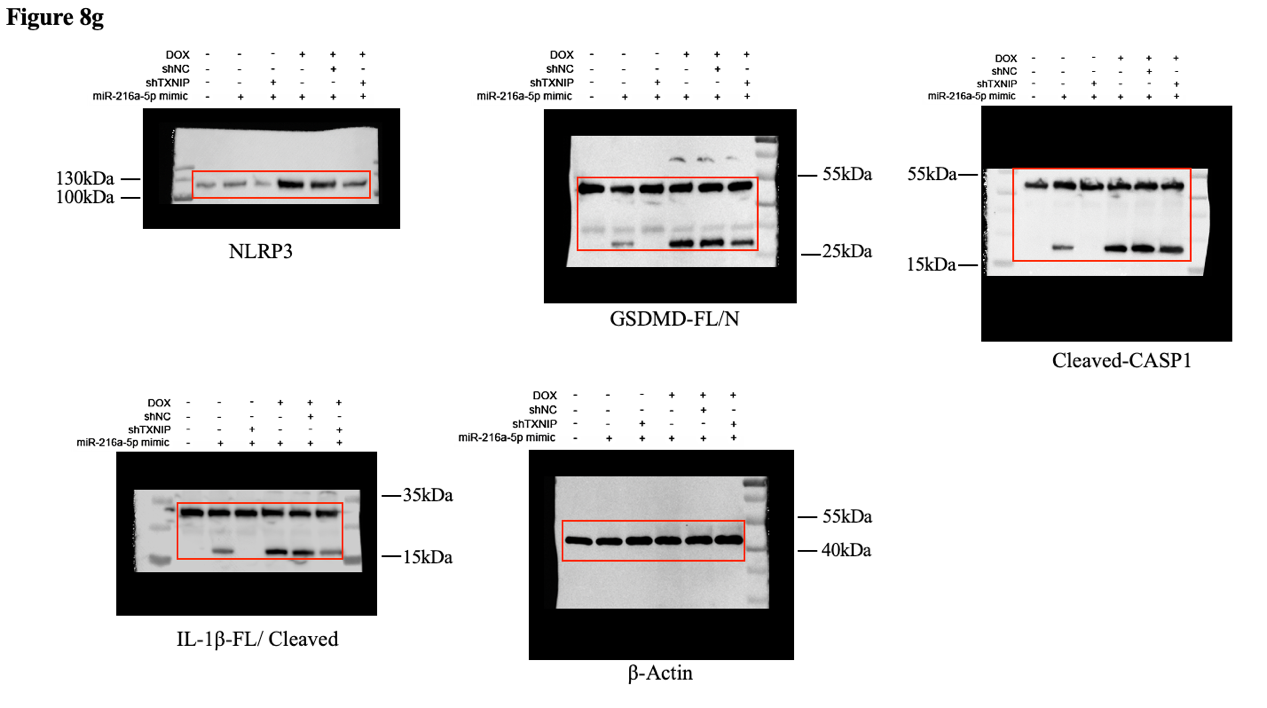


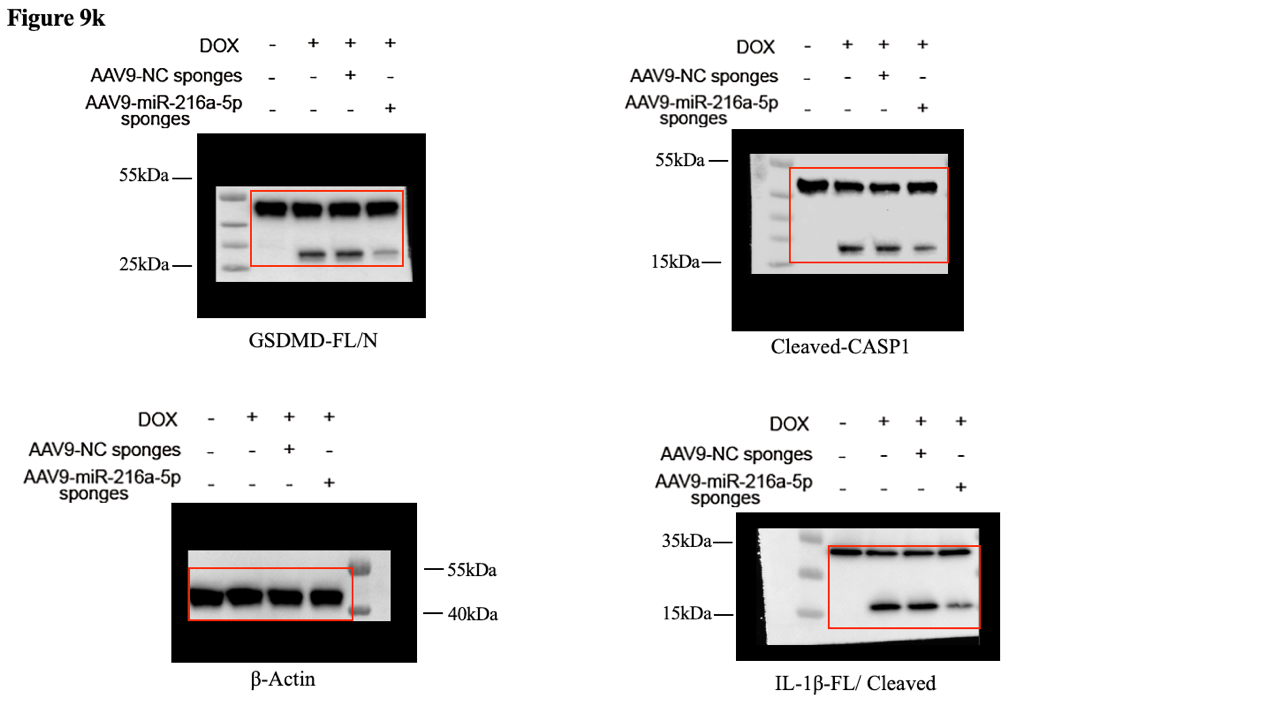


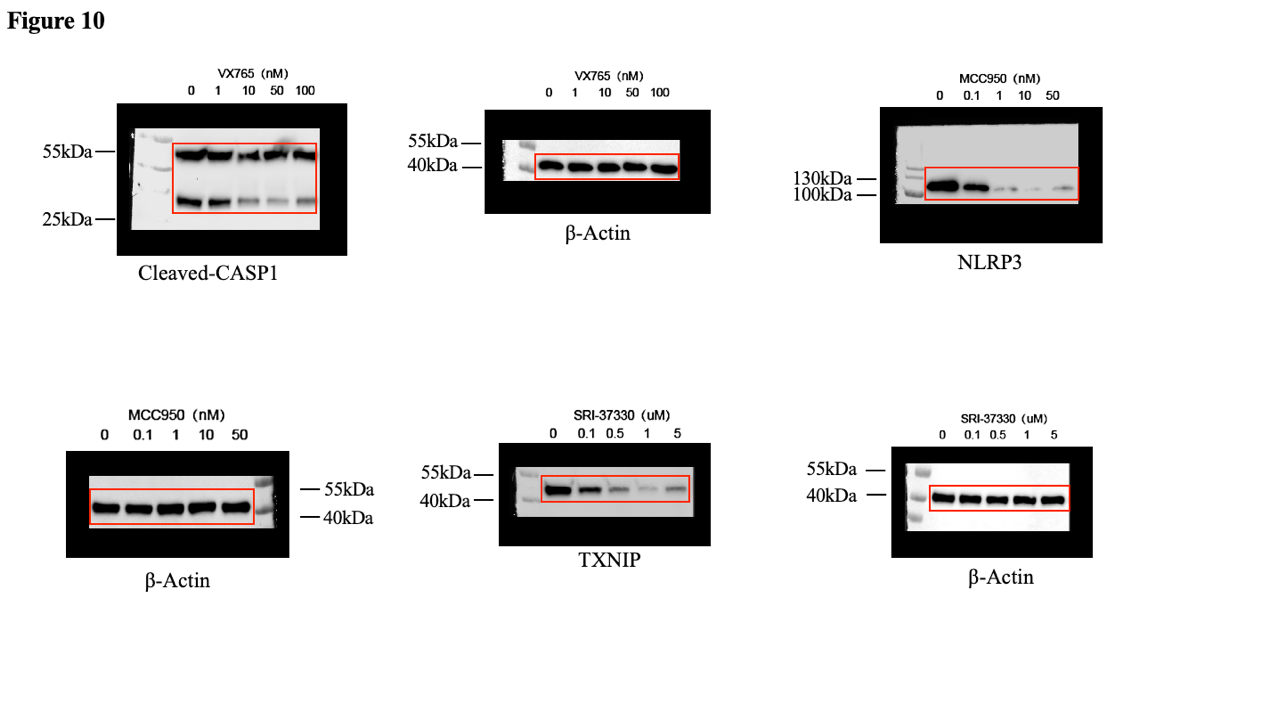


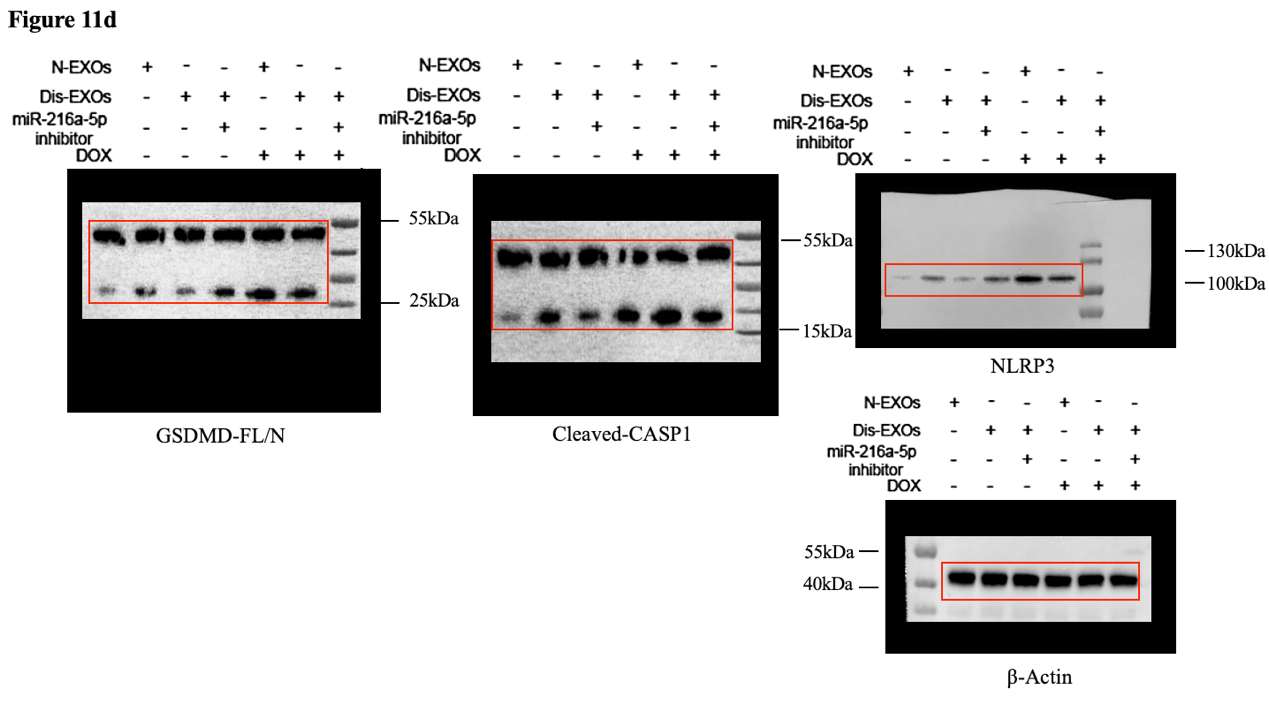


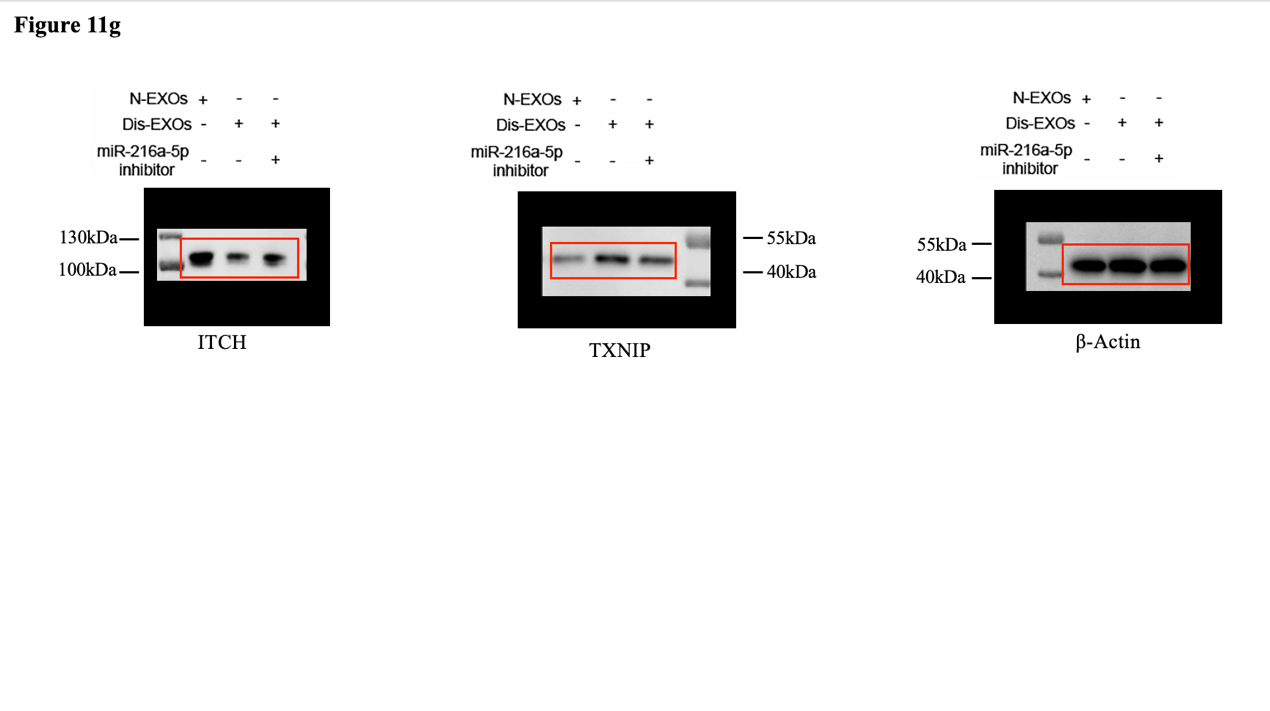


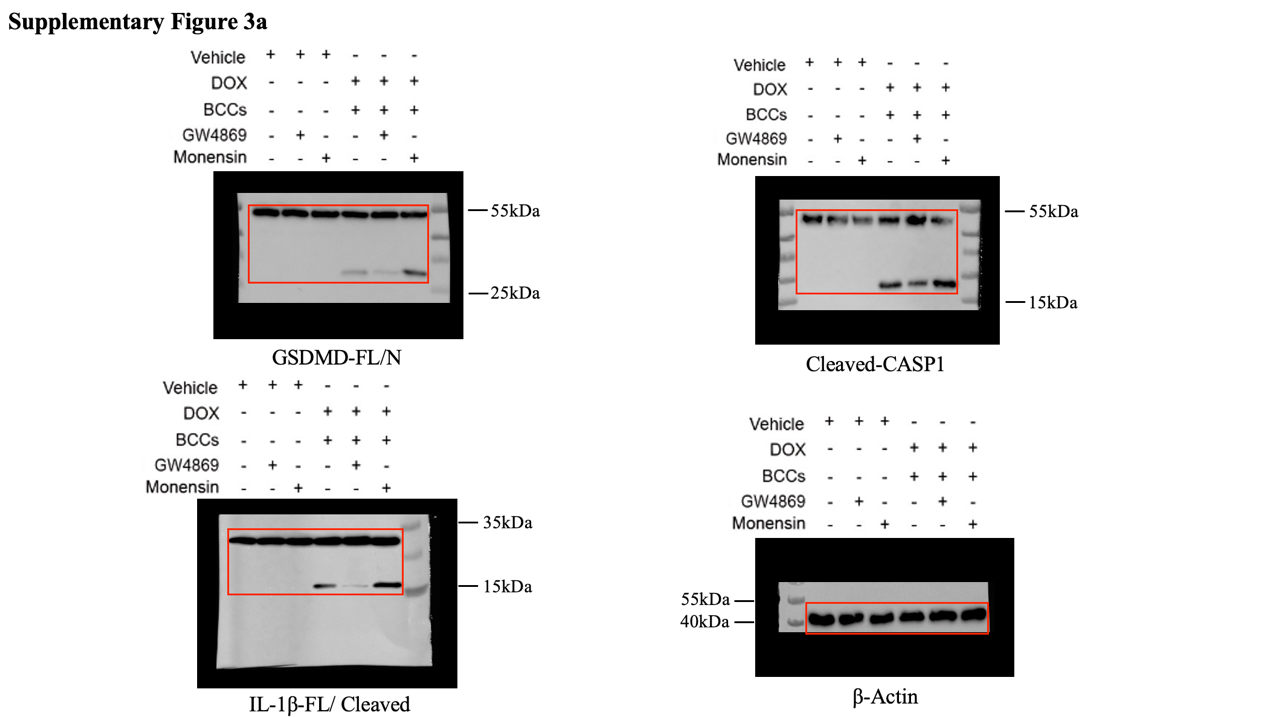


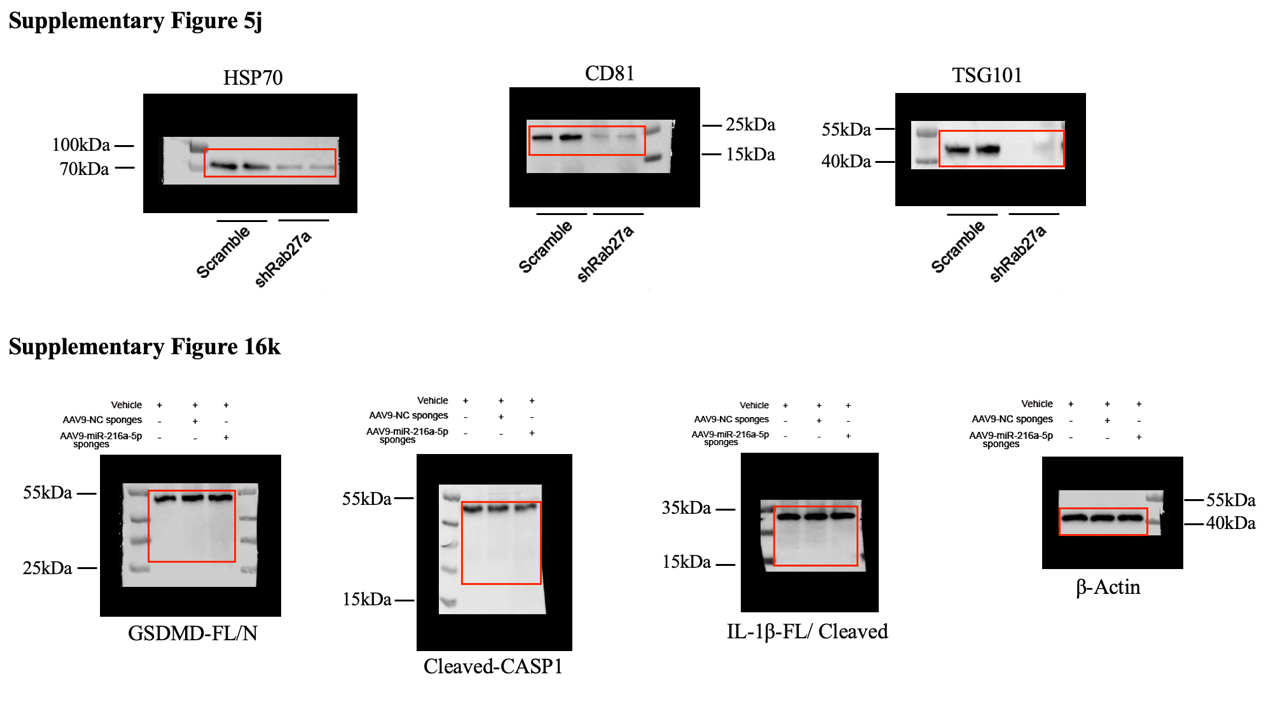


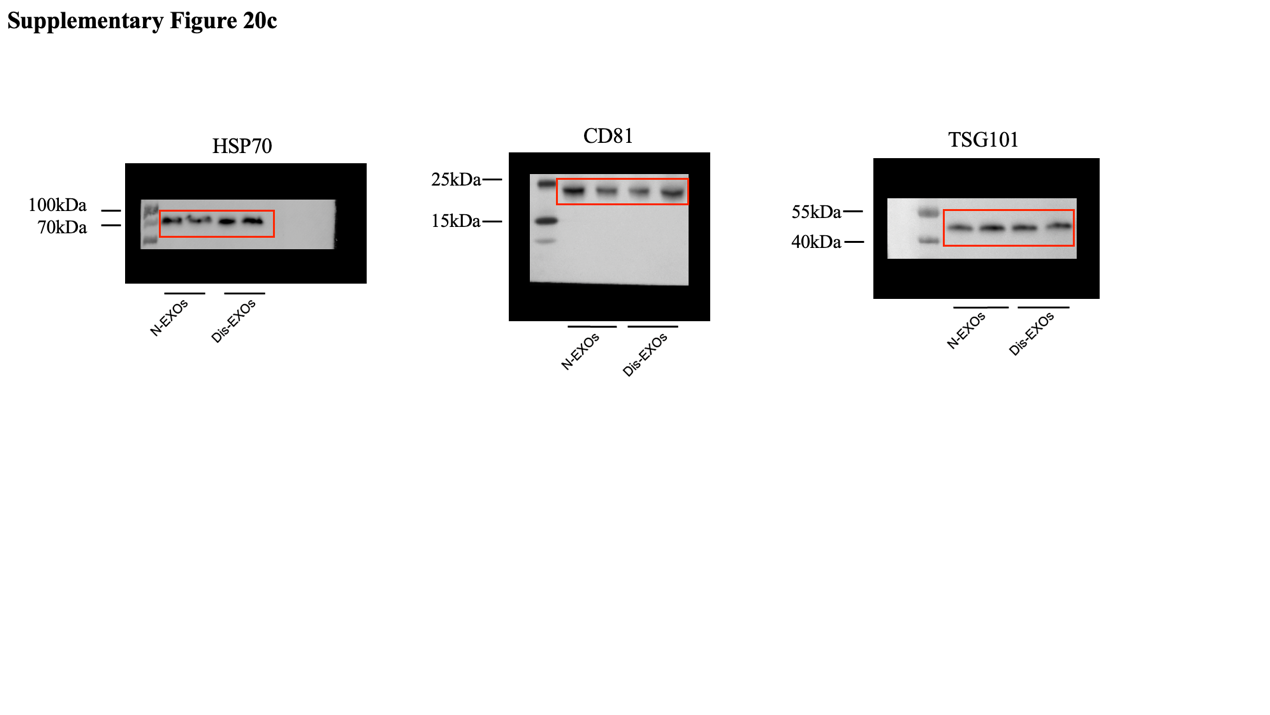

Supplement: Supplementary file 2 — Revised Supplementary data_0403 Mraked up [file 41392_2025_2245_MOESM2_ESM.docx]
